# ACGH detects distinct genomic alterations of primary intrahepatic cholangiocarcinomas and matched lymph node metastases and identifies a poor prognosis subclass

**DOI:** 10.1038/s41598-018-28941-6

**Published:** 2018-07-13

**Authors:** Ruben Jansen, Birte Moehlendick, Christoph Bartenhagen, Csaba Tóth, Nadja Lehwald, Nikolas H. Stoecklein, Wolfram T. Knoefel, Anja Lachenmayer

**Affiliations:** 1 Department of Surgery, Heinrich-Heine University and University Hospital Duesseldorf, Duesseldorf, Germany; 2 Institute of Pharmacogenetics, University Hospital Essen, Essen, Germany; 3 Department of Experimental Pediatric Oncology, University of Cologne, Cologne, Germany; 4 Institute of Pathology, University Hospital Heidelberg, Heidelberg, Germany; 5 Department of Visceral Surgery and Medicine, Inselspital, Bern University Hospital, University of Bern, Bern, Switzerland

## Abstract

Lymph node metastases (LNM) are an important prognostic factor for patients with intrahepatic cholangiocarcinoma, but underlying genetic alterations are poorly understood. Whole genome array comparative genomic hybridization (aCGH) was performed in 37 tumors and 14 matched LNM. Genomic analyses of tumors confirmed known and identified new (gains in 19q) copy number alterations (CNA). Tumors with LNM (N1) had more alterations and exclusive gains (3p, 4q, 5p, 13q) and losses (17p and 20p). LNM shared most alterations with their matched tumors (86%), but 79% acquired new isolated gains [12q14 (36%); 1p13, 2p23, 7p22, 7q11, 11q12, 13q13 and 14q12 (>20%)]. Unsupervised clustering revealed a poor prognosis subclass with increased alterations significantly associated to tumor differentiation and survival. *TP53* and *KRAS* mutations occurred in 19% of tumors and 6% of metastases. Pathway analyses revealed association to cancer-associated pathways. Advanced tumor stage, microvascular/perineural invasion, and microscopic positive resection margin (R1) were significantly correlated to metastases, while N1-status, R1-resection, and poor tumor differentiation were significantly correlated to survival. ACGH identified clear differences between N0 (no LNM) and N1 tumors, while N1 tumors and matched LNM displayed high clonality with exclusive gains in the metastases. A novel subclass with increased CNAs and poor tumor differentiation was significantly correlated to survival.

## Introduction

Cholangiocarcinoma is the second most common liver malignancy worldwide and accounts for nearly 3% of all gastrointestinal cancers^1,2^. Although intrahepatic cholangiocarcinoma (iCCA) accounts only for 10% of all primary liver malignancies, its incidence and mortality rate is increasing worldwide^3,4^. The 5-year survival rate for patients with iCCA varies between <5% and 20–40% depending on the tumor stage at diagnosis. While surgical resection remains to be the only curative therapy so far^5^, the occurrence of LNM is an independent prognostic factor for iCCA with a significant correlation to poor survival^6^. A recent consensus statement recommended to consider lymph node dissection although strong clinical data supporting this approach is still missing^7^. While some centers perform a routine lymphadenectomy in order to improve staging and to increase the amount of prospective information for the patient, others even recommend adjuvant chemotherapy for patients with nodal positive tumors. Nevertheless all clinical trials and studies conducted so far were not able to show a clear benefit for the adjuvant treatment of this disease and data of ongoing clinical trials is still urgently awaited. Although several studies recently performed comprehensive genetic analyses of primary iCCA, a clear understanding of how LNM develop and how advanced tumor stages could be treated is still not available. ACGH has been successfully used in colorectal cancer to identify molecular alterations in primary tumors and their matched metastases^8,9^. As an accepted standard for the detection of copy number alterations (CNA), aCGH yields a high throughput by low costs, providing benefits when working with a large number of samples compared to expensive next generation sequencing technologies^10–12^. Analyses of grouped pairs of primary tumors and matched metastases have recently been used in pancreatic cancer and melanoma to assess the polyclonality and genetic heterogeneity, but are still not reported for intrahepatic cholangiocarcinomas^13–15^. In addition, a CNA based algorithm using CNAs of the cancer founding clone was recently developed to identify cancer types in circulating tumor cells and cell-free DNAs^16^.

Therefore the aim of our study was (1) to analyze copy number alterations in intrahepatic cholangiocarcinomas and their corresponding lymph node metastases and (2) to analyze and correlate clinical and histopathological factors.

## Results

### Clinical and histopathological data correlated with LNM and outcome in iCCA

Twenty-three (38%) patients of our cohort had nodal positive iCCA (Table 1). Patients with LNM had significantly more lymph nodes resected than patients without (15 vs. 7, p < 0.001, Table 1). Advanced tumor stages (T3/4) (*p* = 0.007), smoking (*p* = 0.008), R1 resection (*p* = 0.024), microvascular (*p* < 0.001) and perineural (*p* < 0.001) invasion were significantly correlated with the occurrence of LNM (Table 1 and Supplementary Table 1). Cases without clinical data were eliminated from the analysis. Resection modus had no influence and no differences were seen between different surgical approaches (Supplementary Table 2).

**Table 1:** Histopathological data.

Features	Total (n = 60)	N0 (n = 31)	N1 (n = 23)	*p*-Value
Tumor stage				
T1 + 2	32 (53%)	22 (71%)	9 (39%)	
T3 + 4	22 (37%)	7 (23%)	14 (61%)	0.007
Median tumor size				
Pathology (in cm)	6.6 (1.5–16)	6.3 (0.7–14)	7.1 (2.3–13)	n.s.
Node stage	54	31 (52%)	23 (38%)	
Median number of resected lymph nodes	9 (0–39)	7 (1–13)	15 (1–39)	<0.001
Distant metastasis				
M0	48 (80%)	29 (94%)	17 (74%)	
M1	7 (12%)	1 (3%)	4 (17%)	0.063
Differentiation				
Well	1 (2%)	1 (3%)	0	n.s.
Moderate	29 (48%)	17 (55%)	11 (48%)	n.s.
Poor	25 (42%)	12 (39%)	12 (52%)	n.s.
Tumor surgery				
R0 (tumor-free margin)	48 (80%)	28 (90%)	15 (65%)	
R1	12 (20%)	3 (10%)	8 (35%)	0.024
Vascular invasion				
V0	41 (68%)	29 (94%)	11 (48%)	
V1 (microvascular)	13 (22%)	1 (3%)	11 (48%)	<0.001
Perineural invasion				
Pn0	33 (55%)	25 (81%)	6 (26%)	
Pn1	13 (22%)	4 (13%)	9 (39%)	0.001

**Figure 1:**
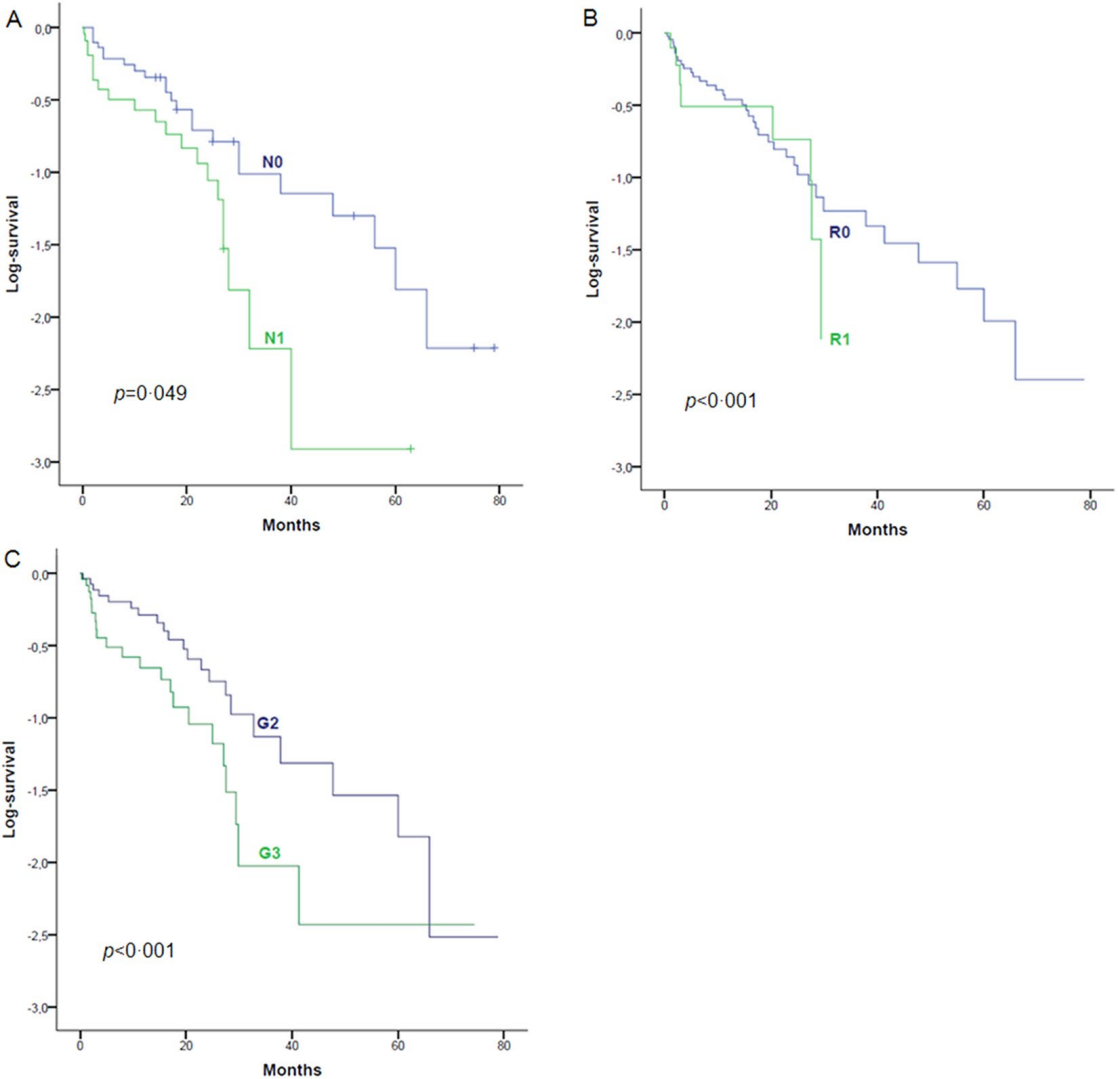
Significant factors affecting survival in iCCA. Patients with LNM showed a significant (*p* = 0.049) worse survival, than patients without LNM. (**A**) R1 resection margin (**B**) and poor tumor differentiation (**C**) were also significantly correlated to a poorer outcome (*p* < 0.001 and *p* < 0.001). Each analysis refers to 60 patients, with one dataset was used for statistical analysis, no censoring was performed.

Kaplan-Meier analysis showed significantly worse survival of patients with LNM, (*p* = 0.049). The median survival rate for patients with N1 tumors was 18 months compared to 26 months for patients with N0 tumors. While R1 (*p* < 0.001) and poor tumor differentiation (*p* < 0.001) were significantly correlated with poor outcome (Fig. 1), the influence of tumor size was not statistically significant (*p* = 0.061, Supplementary Fig. 1). Nevertheless, none of the above factors reached statistical significance in the multivariate analysis. Gender, vascular invasion, perineural invasion and distant metastases did not affect patient survival rates. Patients with LNM developed significantly more (11 vs. 5, *p* = 0.012, Supplementary Table 3) and earlier distant metastases than those with N0 tumors (8 vs. 17 months). Median survival and medium follow-up were 21 months. In our cohort the 1/3/5-year survival rate was 60%, 16% and 12%, respectively.

#### Genetic Alterations in iCCAs and their matched LNM

**Figure 2:**
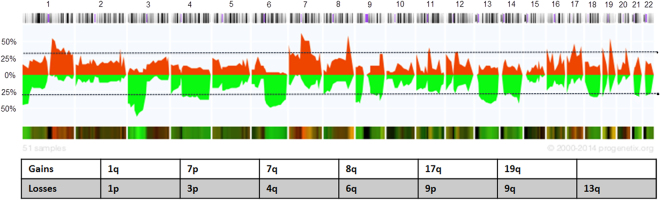
aCGH results. In this penetrance plot all iCCA samples (N1, N0 primary tumors and LNM) are considered together (n = 51). Chromosomal gains are displayed in red and chromosomal losses in green. The chart sums up the gains and losses that are present in at least 30% of the samples. Gains on chromosome 19q have not been described for iCCA so far. The horizontal line indicates the threshold of 30%, the y axis represent the % of cases with aberration.

All tumor samples (primary tumors and LNM) were analyzed by aCGH and several chromosomal gains and losses were detected. In our cohort the overall amount of detected losses was higher compared to the amount of gains. The penetrance plot in Fig. 2 illustrates all detected CNAs. N1 primary tumors harbored more CNAs than tumors without, in N1 primary tumors, a mean number of 45.13 CNAs was detected compared to 37.43 CNAs in N0 tumors (Supplementary Fig. 2). In addition, the maximum amount of CNAs was higher in N1 (3–161 CNAs) compared to N0 tumors (4–137 CNAs) (Supplementary Fig. 2). LNM also harbored more CNAs (44.22), compared to N0 tumors (Supplementary Table 4), however, this result was not statistically significant (One-Way ANOVA by Kruskal-Wallis test with Dunn’s multiple comparisons test, *p* = 0.9896).

**Figure 3:**
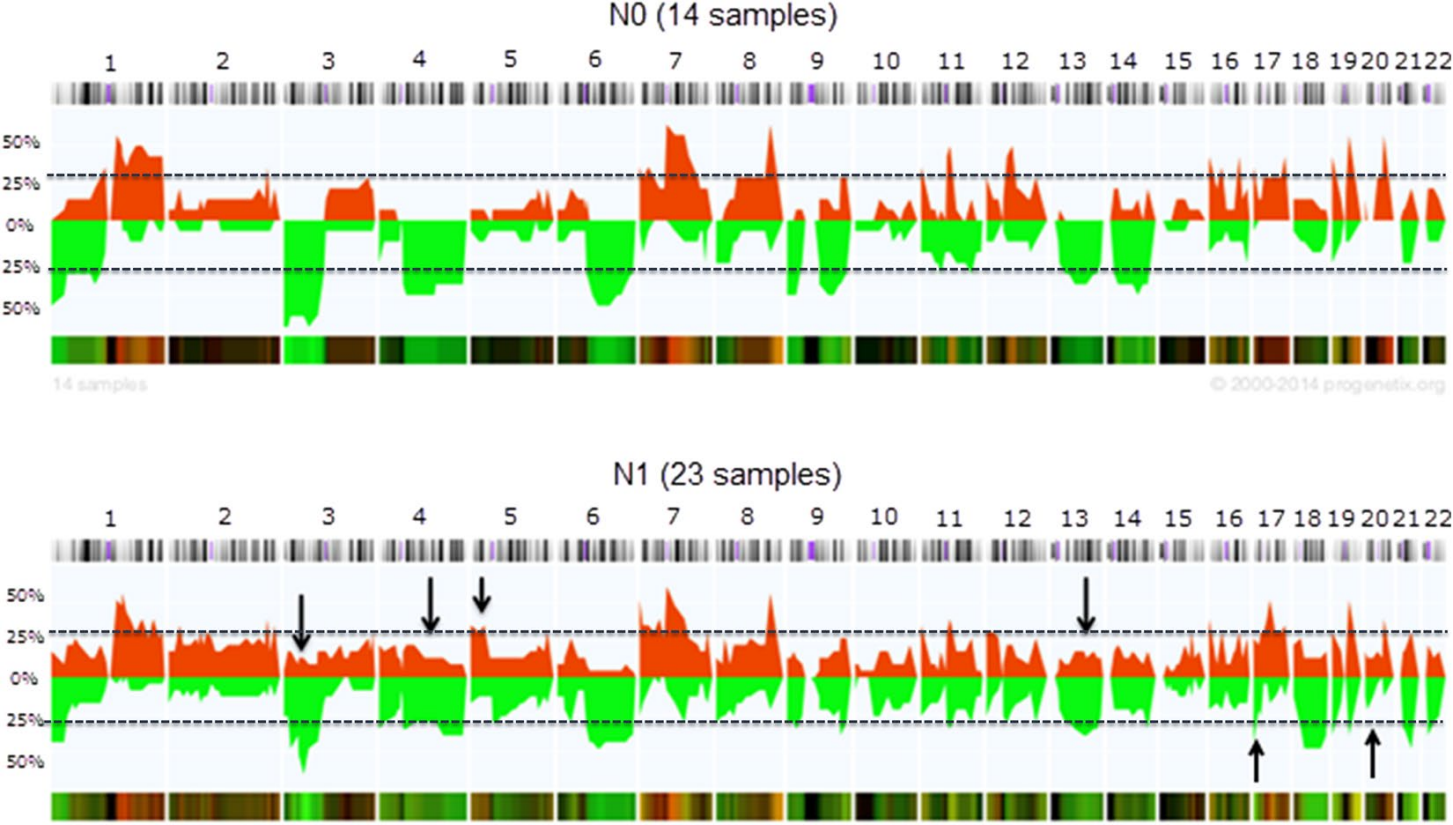
Copy number alterations of N0 and N1 primary tumors. This penetrance plot shows the differences between N0 and N1 primary tumors. Chromosomal gains are displayed in red and losses in green. Additional gains in N1 tumors are indicated (arrows). The horizontal line indicates the threshold of 30%, the y axis represent the % of cases with aberration.

Nodal positive primary tumors showed significant additional gains and losses compared to N0 tumors. Gains were found on chromosome arms 3p (*p* = 0.006), 4q (*p* = 0.016), 5p (*p* = 0.002) and 13q (*p* = 0.039, Fig. 3), losses could be detected on chromosome 17p (*p* < 0.001) and 20p (*p* = 0.006) (Fig. 3). Gains and losses that were detected in N0 tumors were also found in N1 tumors, and harbored relevant cancer-related genes (Supplementary Table 5).

**Figure 4:**
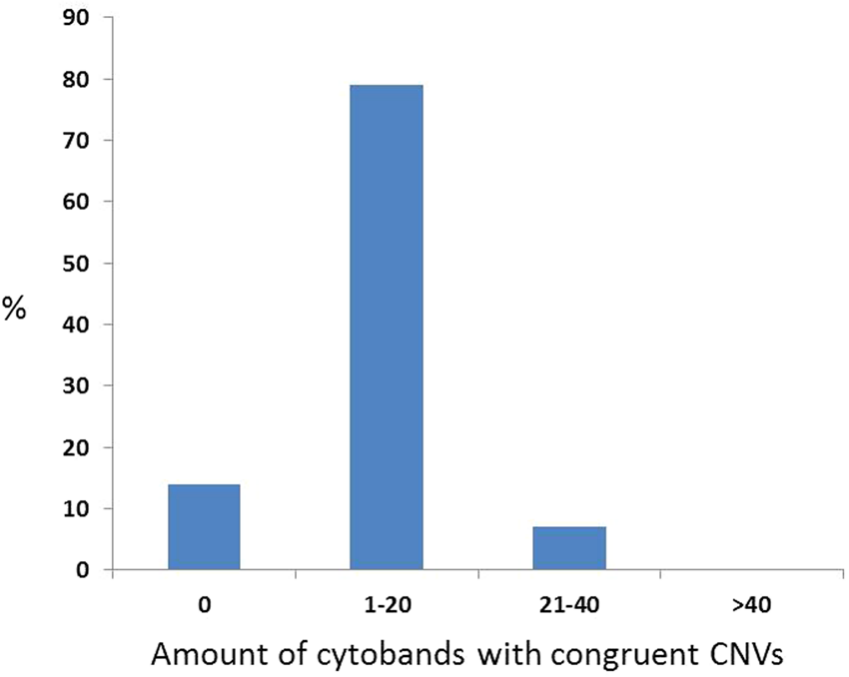
Congruent altered cytobands of matched primary tumors and LNM. This figure shows the number of primary tumor and LNM pairs (in percentage) found with concurrent altered cytobands (resolution = 320 bands per haploid set).

**Figure 5:**
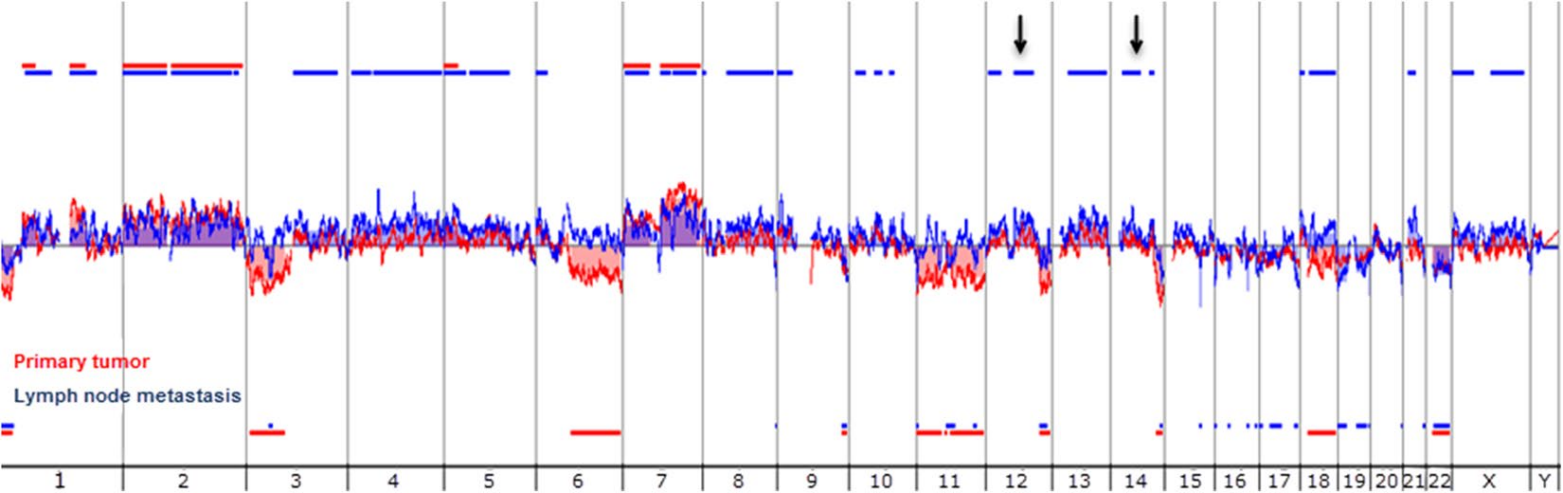
Isolated gains at 12q14 and 14q12. Example of the moving average (colored lines) and alterations (colored areas) from a matched primary tumor and LNM pair. The aCGH profile of the LNM is displayed in blue and the profile of the primary tumor in red. The overlap of these profiles clearly shows additional isolated gains in LNM on 12q14 and 14q12 (arrows) compared to its primary tumor.

When primary tumors and their matched LNM were analyzed, few but specific differences were detected. Only 14% of the pairs had incongruent CNAs, while 86% of the primary tumors and their matched LNM shared the same alterations. Seventy-nine % of these latter pairs shared up to 20 CNAs, while 7% had congruent CNAs in 21–40 cytobands (resolution = 320 bands per haploid set) (Fig. 4). Despite the great similarity between tumors and their corresponding LNM, 79% of the LNM acquired additional specific isolated gains compared to their matched primary tumors: 36% showed isolated gains on chromosome 12q14 and more than 20% displayed isolated gains on chromosome bands 1p13, 2p23, 7p22, 7q11, 11q12, 13q13 and 14q12 (Fig. 5). Supplementary Fig. 3 displays the specific CGH profile of each patient to support these findings. These gains harbor relevant cancer-related genes (Supplementary Table 6). New isolated losses in LNMs occurred infrequently in less than 20% of the samples.

**Figure 6:**
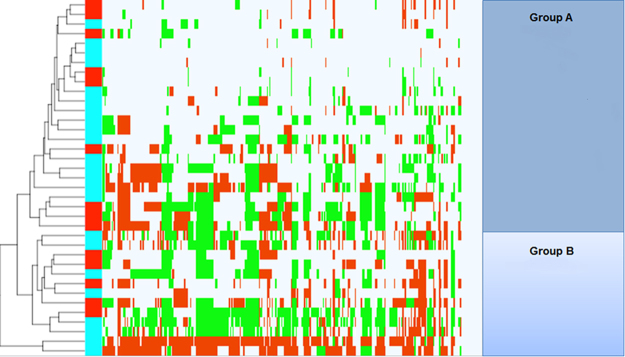
Cluster analysis of primary tumors. This cluster analysis shows two groups of primary tumors. Group B harbored significantly more poorly differentiated tumors and was associated with poor survival rates. Chromosomal losses are displayed in green and gains in red. Blue and red columns on the left side indicate the different primary tumors (N1 = blue; N0 = red).

**Figure 7:**
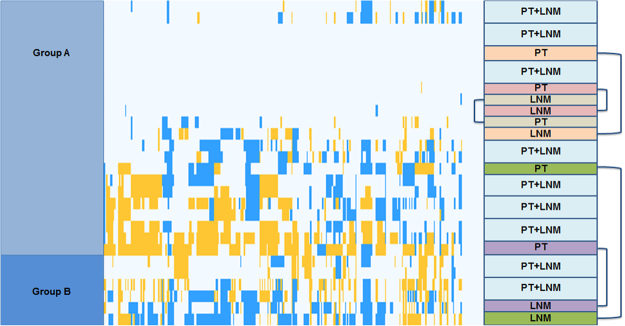
Cluster analysis of primary tumors and LNM. Due to the high genetic similarity LNM and their matched primary tumors clustered next or in great proximity to each other (boxes on the right side). Chromosomal losses are displayed in blue and gains in yellow.

Unsupervised clustering of CNAs of all primary tumors identified two distinct groups (group A and group B) (Fig. 6). Intriguingly, group B tumors were more poorly differentiated compared to group A (G3/4 vs. G1/2, *p* = 0.034) and had a significant shorter survival (13 vs. 31 months, *p* = 0.035) than group A (Supplementary Fig. 4). Subgroup B tumors also harbored significantly more CNAs (60.5 vs. 21.5, *p* = 0.0246, Mann Whitney *U* test) than subgroup A. In addition, the maximum amount of CNAs was higher in subgroup B (161 vs. 71) (Supplementary Fig. 5). Subgroup B also showed significant additional gains and losses compared to subgroup A (Supplementary Table 7) that contained relevant cancer-related genes (Supplementary Table 8). Other analyzed clinical factors (gender, smoking, tumor size, regional lymph node metastasis, distant metastasis, liver cirrhosis, perineural and vascular invasion, tumor surgery and tumor markers) showed no significant correlation to one of the subgroups. When the unsupervised clustering was performed for N1 tumors and their corresponding LNMs, LNM and primary tumors of most of the pairs clustered next or in great proximity to each other due to the high genetic similarity between the pairs (Fig. 7).

We performed mutational analyses for the common and cancer-relevant mutations in the genes *KRAS* and *TP53*. Mutational analysis (18 N1 primary tumors and 18 matched lymph node metastases) revealed mutations of *TP53* in six samples (19%) and mutations of *KRAS* in two samples (6%). Of the six *TP53* mutations, four were detected for Exon 7 (11%) and two for Exon 5 (six %) (4 mutations were found in primary tumors, 2 in LNM). The *KRAS* mutations were detected for Exon 2 (both were found in primary tumors). Mutations in the primary tumor were also detected in the corresponding lymph node metastasis, except for one sample. Since the mutation analyses were only performed in N1 tumors, no information was available for the penetrance in N0 iCCAs. While no CNAs could be detected for the regions harboring the *TP53* mutation, we found one gain on 12p12 (location of the *KRAS* mutation). Interestingly, the amount of CNAs did not differ between tumors with TP53 inactivating mutations and those without.

### Pathway analysis and Therapeutic Target Database analysis

KEGG pathway analysis of the genes located within the additional gains and losses that distinguish N1 from N0 primary tumors, and analysis of the genes located within the isolated gains of the lymph node metastases showed a significant activation of cancer-associated pathways. The genes altered in N1 primary tumors are involved in Wnt-, MAPK-, JAK-STAT-, PPAR-, mTOR-, TGF-β, p53- and VEGF-signaling pathways. An *Ecm-receptor* interaction could also be shown (Supplementary Table 7 and Supplementary Fig. 6). Genes located on the isolated LNM gains are mainly involved in *chemokine-signaling* pathways which also influence cellular growth, cell survival, cell migration and apoptosis (Supplementary Table 9 and Supplementary Fig. 7). For genes located within the additional gains and losses of subclass B, we detected a correlation to the JAK-STAT-, SCLC-, Thyroid cancer-, p53-, DNA replication-, MAPK-, Wnt-, TGF-beta-, Hedgehog- and Chemokin signaling pathways (Supplementary Table 10).

The additional gains and losses in the N1 primary tumors hold 1721 known genes, including 43 genes that are known to play important roles in cancer-associated pathways. Twenty genes located within the N1 CNAs matched as targets (Therapeutic Targets Database) for 41 potential substances, of which 26 are in phase two or a later stage of a clinical trial (Supplementary Table 11). Thirty-five genes located within the LNM CNAs were matched to 206 potential substances, of which 132 are in phase two or a later stage of clinical trial (Supplementary Table 12). The poor prognosis subgroup B also showed additional gains and losses, holding 180 genes known to play important roles in cancer-associated pathways. Fourteen genes had a match as a therapeutic target, revealing 53 potential substances targeting these genes (Supplementary Table 13). For genes located on deleted regions we looked for activating drugs, while drugs for genes on gained regions should work as inhibitors, antagonists, or antibodies. Interestingly, none of the identified drugs have been tested in metastatic iCCA patients so far.

## Discussion

Intrahepatic cholangiocarcinoma is the second most common primary liver tumor with an increasing incidence worldwide and a very poor outcome due to its aggressive tumor biology and limited curative treatment options^17^. LNM have been identified as an important prognostic factor therefore, we aimed to analyze copy number alterations in primary tumors along with their matched LNM and together with important clinical factors.

ACGH of all primary tumors confirmed known chromosomal aberrations for iCCA^18^, but also detected frequent novel gains in 19q not previously described. 19q holds the growth factor *TGFB1* and several genes involved in cancer associated pathways^19^. A recently published analysis suggested that enhanced circulating TGF-β1 is associated with an increased risk of hepatocellular carcinoma, but data for iCCA has not been reported yet^20^. However, careful interpretation is warranted and results need to be validated with other methods in the future, as chromosome 19 is often subject to labeling artifacts in aCGH studies^21,22^. Interestingly, the overall number of CNAs was much higher in N1 tumors compared to N0 tumors with distinctly different CNAs (additional gains on chromosome arms 3p, 4q, 5p and 13q and further losses in 17p and 20p in N1). This might reflect the progression from N0 to N1 with genomic alterations also responsible for metastasis. Accordingly, the *in silico* pathway analyses revealed that chromosomal gains of N1 tumors harbor genes involved in pathways important for tumor progression and metastasis, such as the MAPK-, Wnt-, JAK-STAT-, PPAR-, TGF-β-, p53-, VEGF- and mTOR-signaling pathways^18,19,23,24^. These gains include genes on 5p known to be involved in the JAK-STAT signaling pathway and the development of metastases^25^, Growth Hormone Receptor (*GHR*), which was overexpressed in metastatic breast cancer^26^ and Leukemia Inhibitory Factor Receptor Alpha (*LIFR*), which was involved in chemoresistance of cholangiocarcinoma cells^27^. In addition, gains on 13q have been reported to play an important role for esophagus and bladder cancer, gliomas and glioblastomas^23,28–31^, but not yet for iCCA. This chromosomal area also contains Transcription Factor Dp-1 (*TFDP1*), which plays a crucial role in cell cycle progression^32^ and the oncogene Fms Related Tyrosine Kinase 1 (*FLT1*), known to induce metastasis in breast cancer models^33^. Overexpression of the latter was also significantly associated with poor survival in cholangiocarcinoma^34^.

Significant losses were detected in chromosomal regions harboring important tumor suppressor genes. Chromosome region 17p contains *TP53*, which is lost or mutated in half of all human cancers^35^. It could recently be shown, that the metastatic potential of cholangiocarcinoma cells can be increased by the deletion of *TP53*, underscoring its role for the development of metastasis^35,36^. We also detected mutations for *TP53* in four N1 tumors and two matched LNM. In addition, the in our samples altered regions 20p12 and 20p13 hold the known tumor suppressor genes Phospholipase C Beta 1 and 4 (*PLCB1*, *PLCB4*) and Casein Kinase 2 Alpha 1 (*CSNK2A1*), all previously described to be associated with advanced tumor stages and bad outcome in different cancers but not yet in iCCA^37–39^. Taken together aCGH is able to detect clear differences between N1 and N0 iCCAs with several significant gains and losses in N1 tumors harboring important cancer-related genes that might play an important role for the development of LNM.

Pairwise comparison of primary tumors and corresponding LNM revealed a great similarity regarding chromosomal gains and losses and the amount of CNAs, but also detected additional, unique isolated gains on the chromosome band 12q14 (36%) and on 1p13, 2p23, 7p22, 7q11, 11q12, 13q13 and 14q12 (>20%). Matched genomic analyses of primary tumors and their corresponding metastases are rarely reported in the literature, although they have been proposed to be an ideal tool to identify “driver” molecular changes responsible for the development of metastases^40^. The specific isolated gain on chromosome band 12q14 that occurred in 36% of our LNM holds several interesting genes like NF-κB activating kinase TANK Binding Kinase 1 (*TBK1*), transcription factor High Mobility Group AT-Hook 2 (*HMGA2*), MDM2 Proto-Oncogene (*MDM2*), Glucosamine (N-Acetyl)-6-Sulfatase (*GNS*) and Cullin Associated And Neddylation Dissociated 1 (*CAND1*). The latter one is known to be important for chromosomal stability and has recently been reported to play a key role in prostate cancer^41,42^. *TBK1* was successfully targeted in oral squamous cell carcinoma and osteosarcoma leading to a decrease in tumor activity^43,44^ and *MDM2* amplifications are known to be involved in tumorigenesis of liposarcoma^45–47^. Furthermore, several studies have shown the importance of HMGA2 expression for metastasis and tumor progression in different malignancies but not yet in iCCA^48–53^. To the best of our knowledge this is the first matched primary tumor-LNM analysis in iCCA describing a high clonality between primary tumors and LNM.

Unsupervised clustering of all CNAs revealed one group of patients with a significant correlation to poor survival and poor tumor differentiation independent of the lymph node status. This poor prognosis group harbored significantly more CNAs including chromosomal alterations at 7p, 9p and 17q that have previously been described to be related to poor tumor differentiation and worse survival in iCCA. Genes located in these regions include C-X-C Motif Chemokine Receptor 4 (*CXCR4*), which inhibited tumor cell proliferation and neural invasion in cell lines of hilar cholangiocarcinoma when silenced^54^, Toll Like Receptor 2 (*TLR2*), known to promote cell migration and invasion by modulating NF-κB pathway in iCCA^55^, and Interleukin-8 (*IL8*), associated to advanced TNM stages and recurrence of hilar cholangiocarcinoma^56^. Other altered regions characterizing this poor prognosis subclass include C-X-C Motif Chemokine Ligand 5 (*CXCL5*), known to promote growth and metastasis in iCCA^57^, transcription factor FOS Like 1 (*FOSL1*) and Erb-B2 Receptor Tyrosine Kinase 2 (ErbB2), both important for tumorigenesis of cholangiocarcinoma^58,59^.

Molecular classes are increasingly reported for several different cancers including iCCA. They are often based on broad genetic analyses including whole genome RNA expression, high-density single-nucleotide polymorphism arrays and mutational analyses with correlations to important clinical and prognostic factors^60^. While their prognostic relevance and the biological significance needs to be thoroughly validated before a clinical application in iCCA might be feasible, the general advantages of using cancer hallmarks as cancer biomarkers in form of gene signatures and algorithms based on large patient cohorts and a training/validation set approach has now repeatedly been shown for several different cancers in the literature^61–63^. Finally, Therapeutic Targets Database (TTD) analyses revealed relevant drugs targeting genes located in the CNAs of our N1 primary tumors, LNMs and the samples of the poor prognosis subclass, that could be tested in preclinical models of iCCA. In addition, altered regions that did not match with known anti-tumoral compounds, should be explored as potential targets for new anti-tumoral therapies in the future. In respect to the suspected high chromosomal instability of the samples of our poor prognosis class, PARP inhibitors might be considered to overcome poor responses to classical chemotherapy^64^.

While our main study results are centered around the genomic differences and similarities of iCCA and their corresponding LNMs, we were also able to confirm several recently reported clinical and histopathological factors associated to LNM and outcome of intrahepatic cholangiocarcinoma. In addition we identified R1-resection and smoking to be significantly associated to LNM in iCCA in the univariate analysis.

ACGH of iCCA primary tumors and LNM revealed known and novel CNAs which might be responsible for tumor progression and the development of LNM. For the first time, a matched analysis of primary iCCA tumors and their corresponding LNM was performed showing highly similar CNAs with very specific novel alterations in the LNM. Multiple genes located in the altered regions of our cohort are known to be involved in cancer-associated pathways and have been reported as potential targets for new anti-tumoral treatment strategies. Lastly and most importantly, we identified a novel molecular subclass with a high amount of CNAs and a significant association to poor tumor differentiation and poor prognosis.

## Materials and Methods

### Statistical analysis

Statistical analysis was performed using SPSS software (version 22.0). Demographic data and baseline characteristics of the patients were analyzed by calculating frequencies and percentages for discrete variables and means, maximum and minimum for continuous variables. The Kolmogorov-Smirnov-Test was used to assess normality of tumor markers, nodal stage and tumor size. T-test was then used to test differences between nodal stage, tumor marker levels and tumor size (for normal distribution). Differences in etiological data, tumor characteristics and chromosomal alterations were tested with the Chi-squared test. Overall survival was assessed with the Kaplan-Meier estimator and Cox regression analysis for the multivariate analysis. A *p*-value of 0.05 or less was considered significant.

### Human Tissue Samples

A total of 78 formalin-fixed, paraffin-embedded (FFPE) samples were collected from 60 patients diagnosed with intrahepatic cholangiocarcinoma and resected between 2003 and 2013 at the Department of General-, Visceral- and Pediatric Surgery of the University Hospital Duesseldorf, Germany. During surgery, most patients received a complete lymph node dissection. Pathological diagnosis of iCCA was always confirmed by an expert pathologist and immunostaining was performed in most of the cases (44/60). Clinical factors and tumor characteristics of the patients who underwent liver surgery are described in Supplementary Tables 1 and 2. Follow-up data was available for 59 patients (one patient was lost to follow-up). The study was approved by the institutional ethics committee of the University Duesseldorf, Germany (#3821). We hereby confirm that all research was performed in accordance with relevant guidelines and regulations and informed consent was obtained from all participants and/or their legal guardians.

### Immunohistochemistry

Immunohistochemistry was performed on one tumor tissue block, representative for the whole lesion. At first 4 µm thick paraffin sections were cut and dried overnight. For immunohistochemical analysis the following antibodies were used: CK7 (Dako, Denmark, monoclonal antibody, clone OV-TL 12/30, dilution 1/6000) and a CK20 (Dako, Denmark, monoclonal antibody, clone Ks20.8, dilution 1/400), CK8 (Becton, Dickinson and Company, monoclonal antibody, clone CAM5.2, dilution 1/400), CK19 (Dako, Denmark, monoclonal antibody, dilution 1/200) and CEA (Dako, Denmark, polyclonal, dilution 1/40.000). Tumor tissue was considered as iCCA, if CK 7 and CK 19 showed positive staining and cytokeratin 20 showed a negative staining^65^. The stainings were carried out using an Avidin–Biotin method with antigen retrieval. The staining was performed on an automated Ventana IHC instrument with a Ventana basic DAB detection kit. Our immunohistochemical analysis was validated through positive and negative controls (by omitting the primary antibody).

### DNA extraction

Invasive tumorous tissue was distinguished from non-neoplastic tissue with two-µm-thick H&E sections. Tumor tissue and non-neoplastic tissue then were macrodissected and DNA extraction and sample preparation was performed from ten nine-µm-thick FFPE sections according to the manufacturers’ protocol for FFPE samples (QIAamp DNA FFPE Tissue Kit QIAGEN, Hilden, Germany). DNA concentration was estimated with the Qubit® 2.0 Fluorometer (Life Technologies, Carlsbad, California, USA) and considered as sufficient, if the DNA concentration was 100 ng/µl or higher. DNA quality was evaluated by measuring fragmentation of the DNA using Agarose gel electrophoresis.

### Array comparative genomic hybridization

ACGH analyses on oligonucleotide arrays were carried out according to the manufacturers‘ protocol (Agilent Oligonucleotide Array-Based CGH for Genomic DNA Analysis, Version 6.4, August 2011, G4410-90010). Basically, for each sample non-neoplastic tissue DNA from the same patient was used as a reference. Tumor cell and reference gDNA were both random-primed labeled with Cyanine 5-/Cyanine 3-dUTP and hybridized on the 8 × 60 k-DNA-microarray chips. The array slides were washed and subsequently scanned using the Microarray Scanner G2565CA by Agilent Technologies with 3 µm resolution and 16 bit color depth.

### Analysis of aCGH Data

The generated data from the scanned images were extracted with the Feature Extraction software (Agilent Technologies, Version 10.7.3.1, Protocol CGH_107_Sep09) and output files were imported into the Genomic Workbench 5.0.14 software. We used the aberration detection method-2 (ADM-2) algorithm with a threshold of 6.0 and the diploid peak centralization for evaluating copy number alterations (CNA). The ADM-2 algorithm, has been shown to provide reliable results in CNA detection of cancer cells^66^. This algorithm searches for intervals in which a statistical score based on the average quality weighted log ratio of the sample and reference channels exceeds a specific threshold. For our analyses we used a semi-stringent threshold of 6.0 to reduce false-positive results and enhance the detection of true-positive copy number alterations. Since cancer cells often show major aberrations and a dominant ploidy deviant to 2, the diploid peak centralization algorithm is the method of choice to normalize the aCGH fluorescence ratios, since it centers the data so that the log ratios of the probes, that are in copy number 2 regions are centered around zero, instead of making the most-common-ploidy the new zero point. An aberration filter with a minimum log2ratio of ±0.25 and a minimum number of 3 affected oligonucleotides was defined. Further analyses of group and pairwise comparisons were performed using data matrix charts with a resolution of 320 mphs. Data matrix charts were generated with the Genomic Workbench 5.0.14 software, displaying CNA as binary code (1 = gain, −1 = loss and 0 = no alteration). Significance of differences in the chromosomal alterations was tested with the Chi-squared test as described above (in der Statistical Anaylsis section). Penetrance plots of the CNA were generated using the internet based genomic copy number data curator progenetix^67^. A threshold of 30% was chosen to clearly distinguish true CNAs from background noise. Unsupervised clustering of microarray data was performed using Eisen cluster algorithm with settings to euclidean distance and pairwise maximum linkage to compare the similarity of the samples^68^. These options led to empirically good performance in analyses in comparison of DNA microarray expression and copy number data.

### Mutational analysis

Mutational analysis was performed for the oncogene *Kirsten Rat Sarcoma Viral Oncogene Homolog* (*KRAS*) (Exon 2) and the *tumor suppressor p53* (*TP53*) (Exon 5 and 7). Therefore 18 N1 primary tumors and 18 matched lymph node metastases were analyzed. Polymerase chain reaction (PCR) was performed under the following conditions: 12.5 μl DreamTaq Green PCR Master Mix (Thermo Fisher Scientific, Schwerte, Germany), 0.5 μl Primer-Mix (10×, final concentration 0.2 μM), 11.5 μl H2O and 0.5 μl Template DNA were mixed and the following program was executed in a thermal cycler: 95 °C for 3 min, 95 °C for 30 sec, melting temperature (Tm) for 30 sec, 72 °C for 20 sec, repeat 30 times and finally 72 °C for 5 min. The primer sequences and Tm can be found in Table 14. The PCR products were purified according to the manufacturers’ protocol (MinElute PCR Purification Kit, QIAGEN, Hilden, Germany). The analysis was validated through positive and negative controls. Sanger sequencing was performed at GATC Biotech (Konstanz, Germany).

### Therapeutic Targets and Pathway Analysis

Regions with isolated amplifications in lymph node metastases compared to their corresponding primary tumors were further analyzed for known genes and matched against the Therapeutic Targets Database (TTD)^69^. We used gene symbols as possible druggable targets and searched for approved drugs or promising substances in clinical trials that would target these genes. In addition genes located in the regions with chromosomal gains and losses, distinguishing N1 from N0 primary tumors, were analyzed for their involvement in cancer-associated pathways and matched against the TTD. Pathway analyses were performed using KEGG pathway analysis, with hypergeometric testing and the R function p.adjust for this purpose^70^. Significance level was adjusted to always identify the ten pathways with the most significant *p*-values and a minimum number of two genes were required for a pathway to be considered.

## Electronic supplementary material

Supplementary Information
