# Supplementary material for: ACGH detects distinct genomic alterations of primary intrahepatic cholangiocarcinomas and matched lymph node metastases and identifies a poor prognosis subclass
Source: Sci Rep. 2018 Jul 13;8:10637. doi: 10.1038/s41598-018-28941-6 (PMC6045619; doi:10.1038/s41598-018-28941-6)
Supplement: Supplementary file 1 — Supplementary Information [file 41598_2018_28941_MOESM1_ESM.pdf]

Supplementary Information

ACGH detects distinct genomic alterations of primary intrahepatic cholangiocarcinomas and matched lymph node metastases and identifies a poor prognosis subclass

Authors: Ruben Jansen[1], Birte Moehlendick[1,2], Christoph Bartenhagen[3], Csaba Tóth[4], Nadja Lehwald[1], Nikolas H. Stoecklein[1], Wolfram T. Knoefel[1], Anja Lachenmayer[1,5 *]

1. Department of Surgery, Heinrich-Heine University and University Hospital Duesseldorf, Duesseldorf, Germany
2. Institute of Pharmacogenetics, University Hospital Essen, Essen, Germany
3. Department of Experimental Pediatric Oncology, University of Cologne, Cologne, Germany
4. Institute of Pathology, University Hospital Heidelberg, Heidelberg, Germany
5. Department of Visceral Surgery and Medicine, Inselspital, Bern University Hospital, University of Bern, Switzerland.

Corresponding Author:

PD Dr. med. A. Lachenmayer

Department of Visceral Surgery and Medicine

Inselspital

Bern University Hospital

University of Bern

Switzerland

Email: anja.lachenmayer@insel.ch

Suppl. Fig. 1

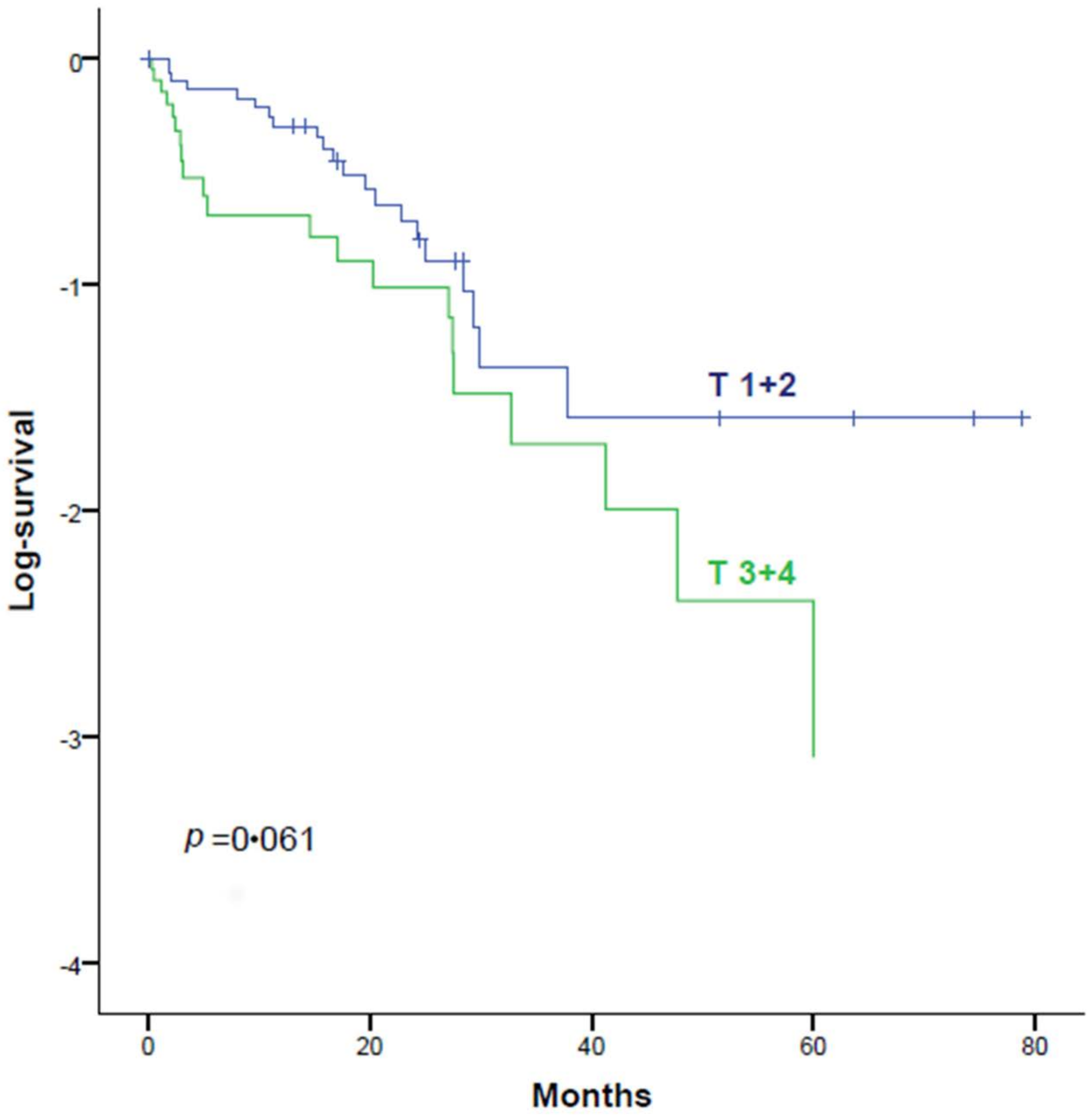

Suppl. Fig. 2

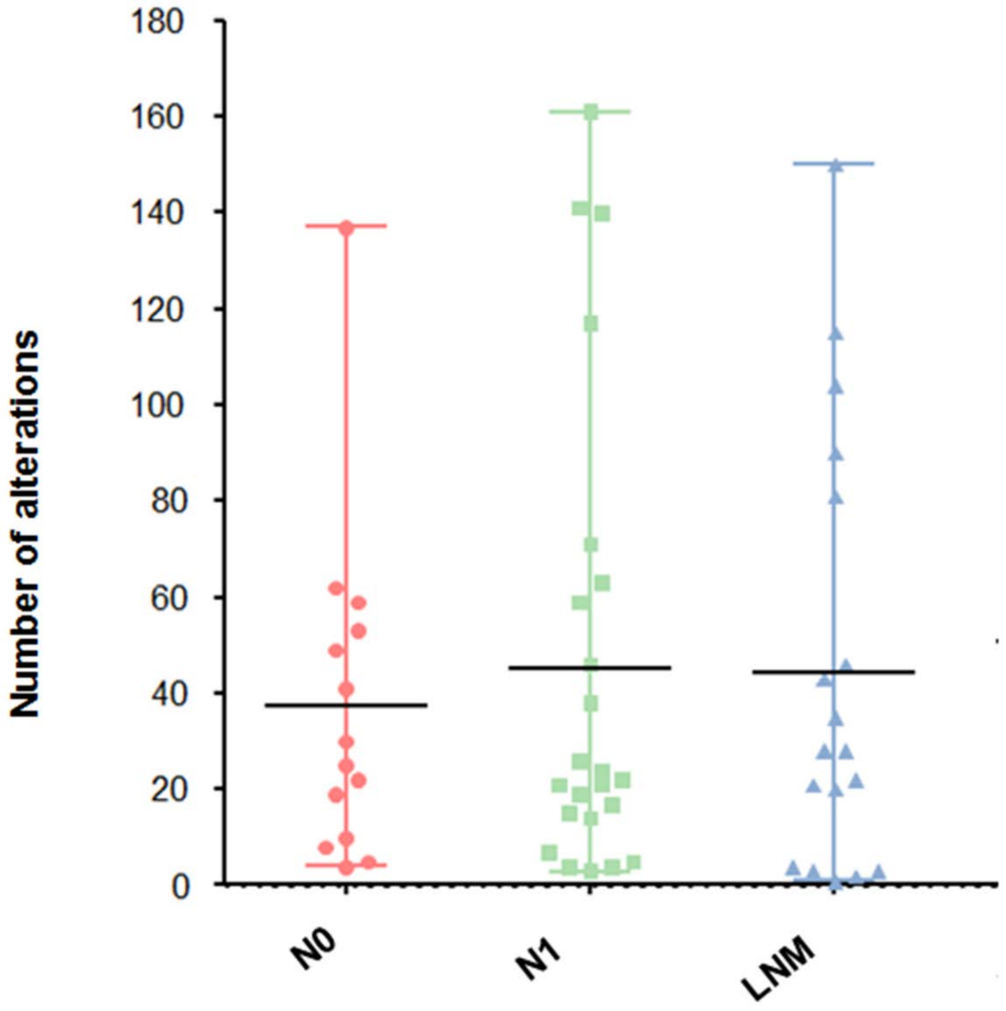

180
160
140
120
100
80
60
40
20
0
Number of alterations
N0
N1
LNM

Suppl. Fig. 3

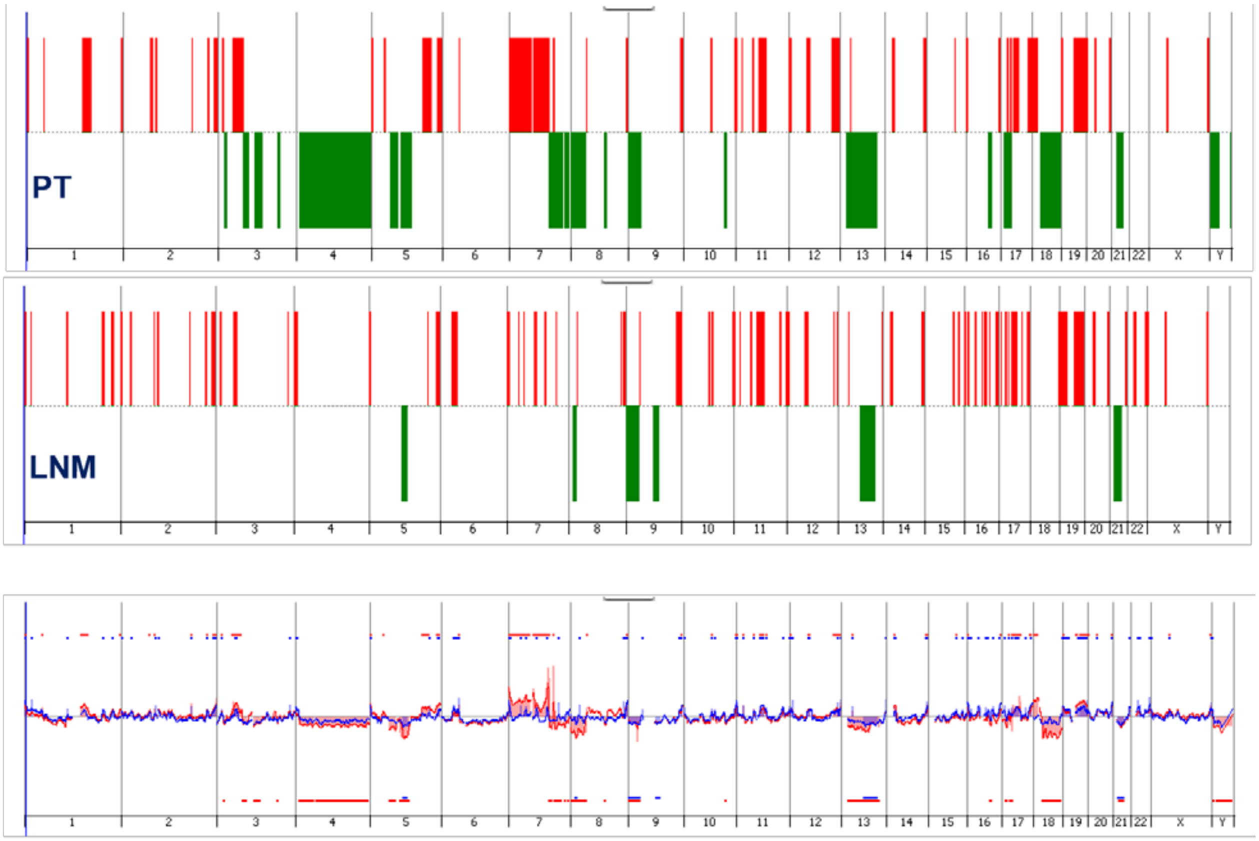

blue=LNM, red=primary tumor

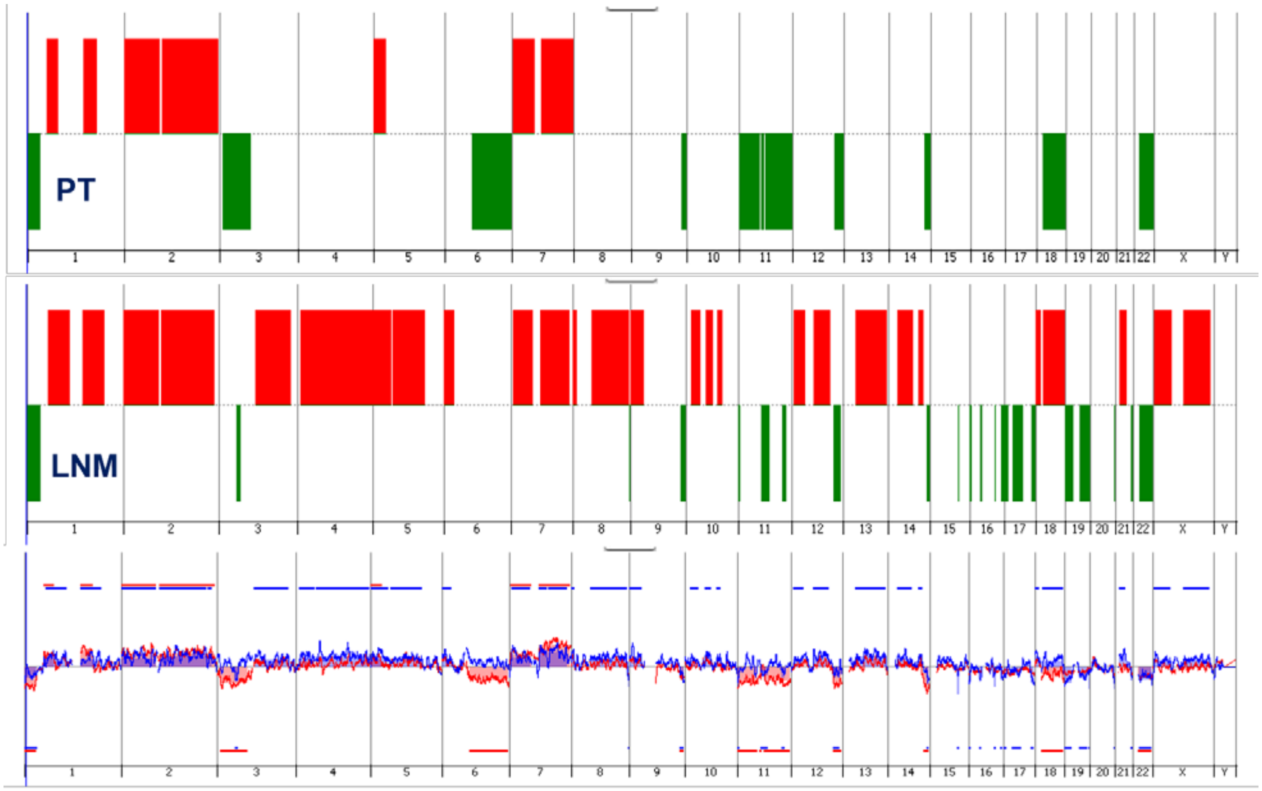

blue=LNM, red=primary tumor

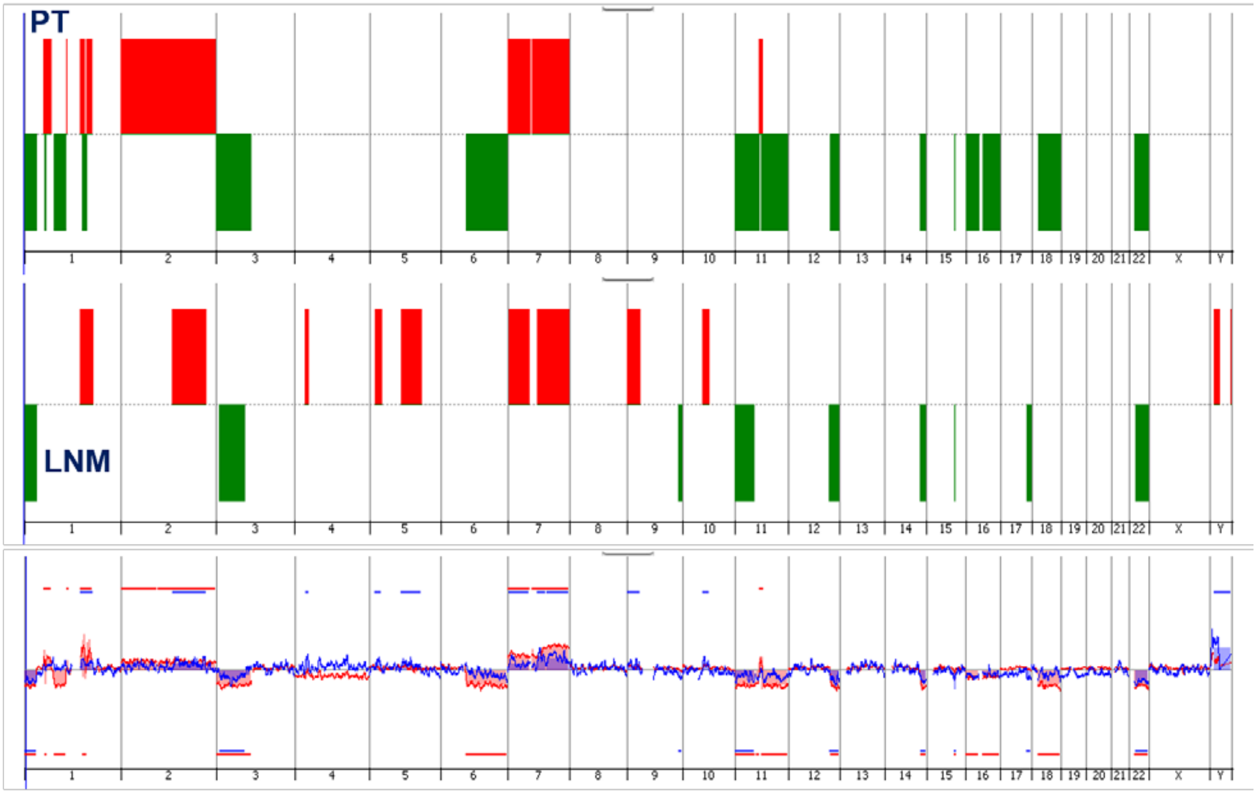

blue=LNM, red=primary tumor

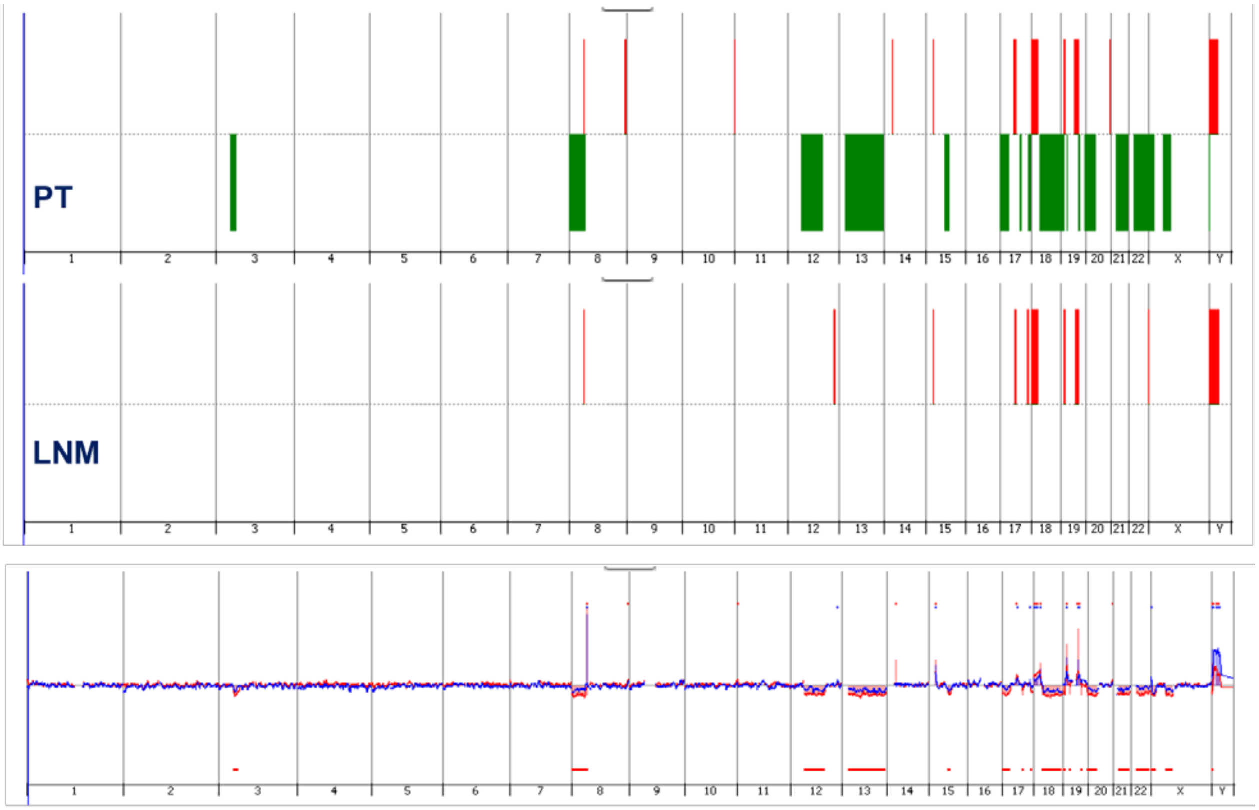

blue=LNM, red=primary tumor

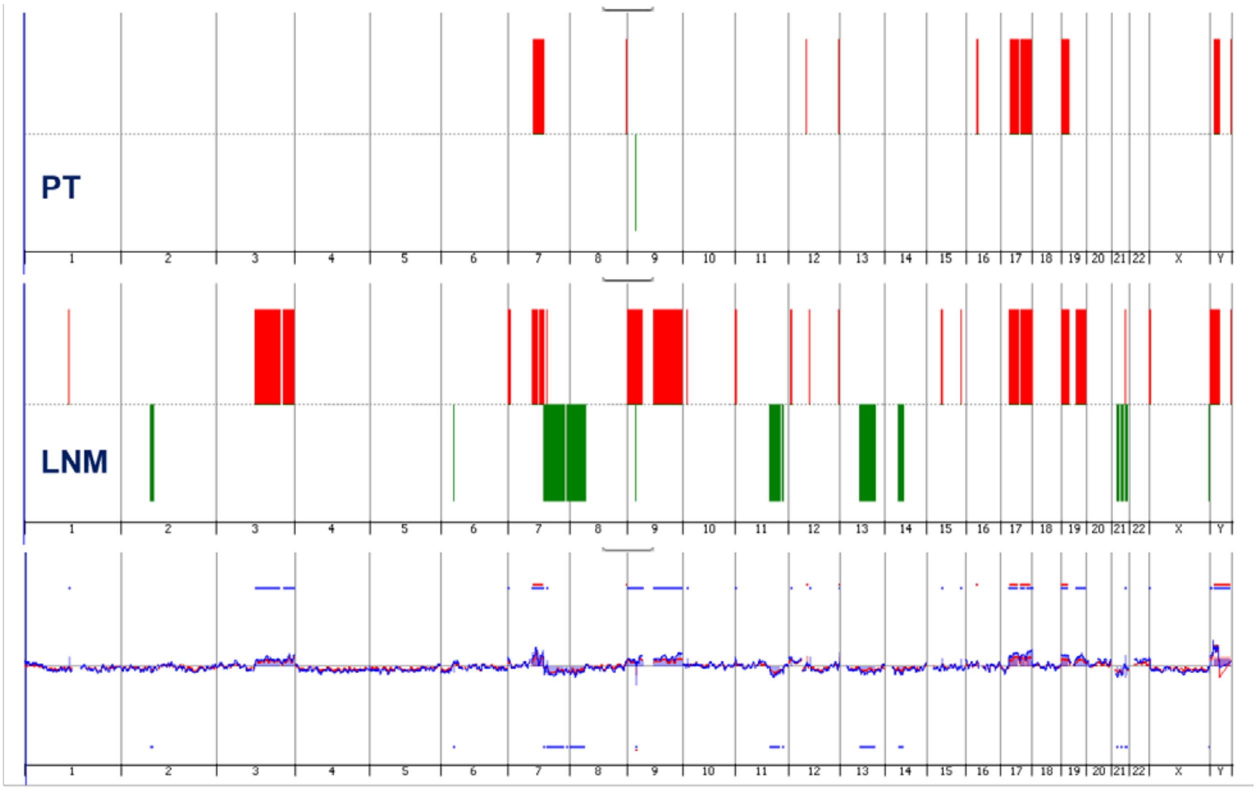

blue=LNM, red=primary tumor

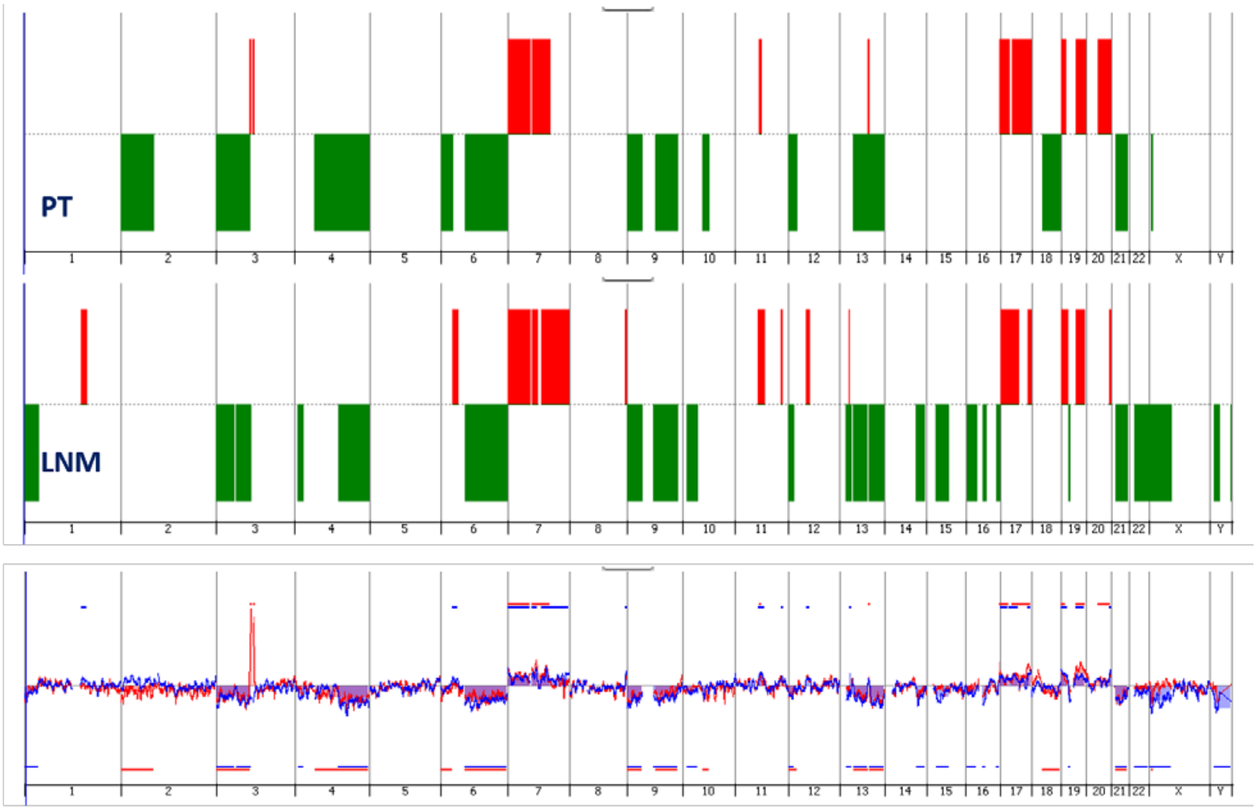

blue=LNM, red=primary tumor

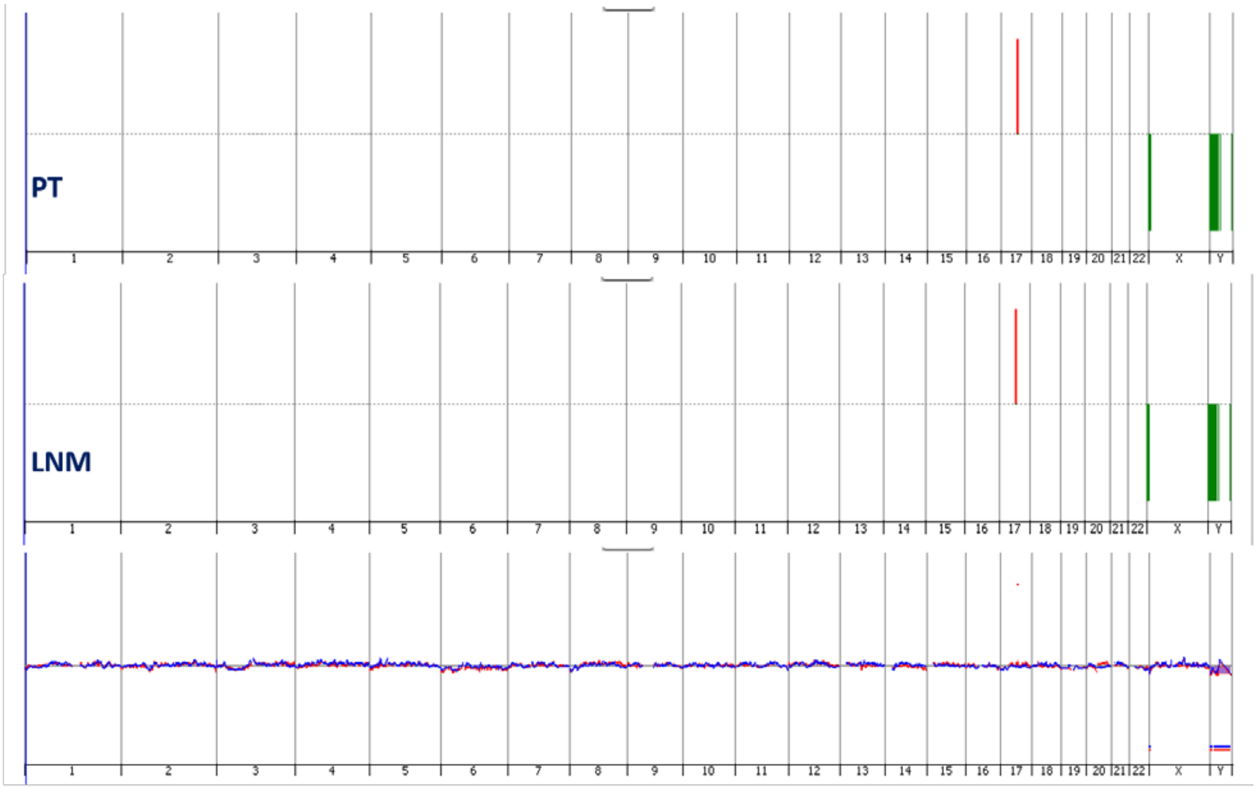

blue=LNM, red=primary tumor

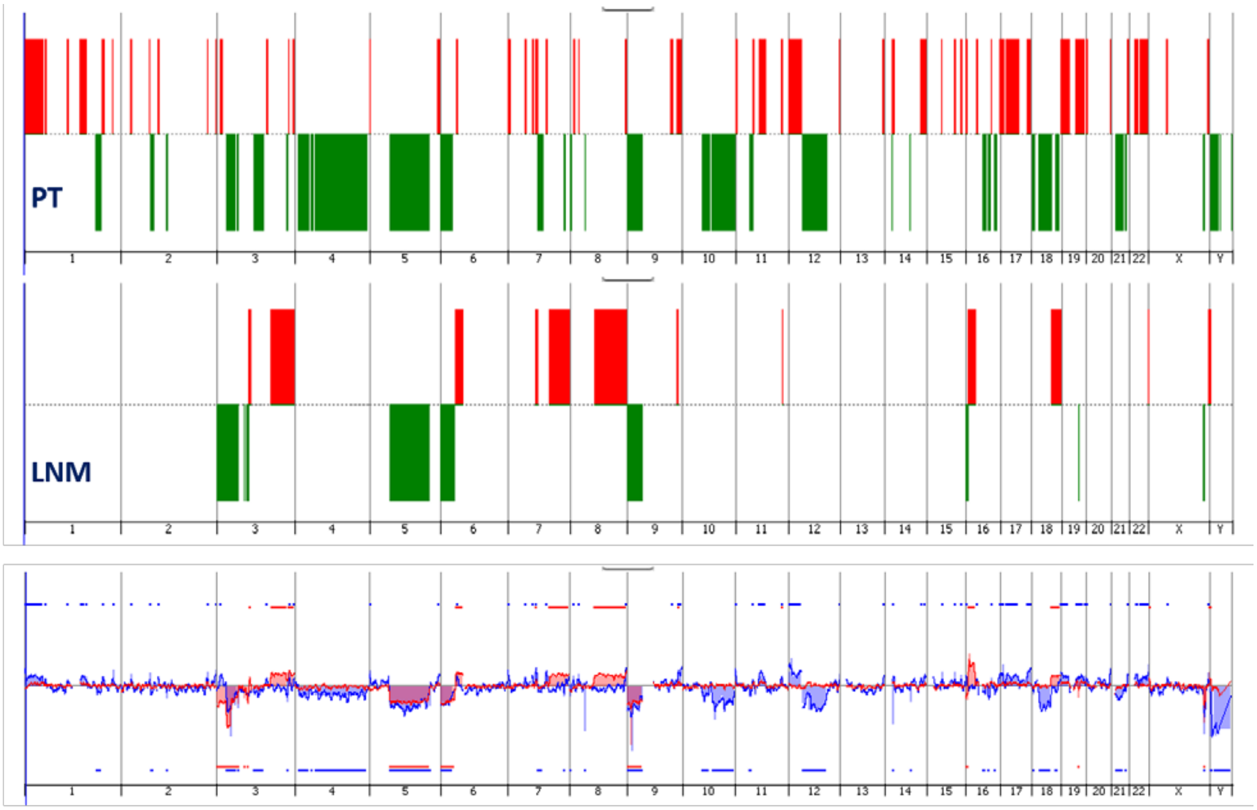

blue=LNM, red=primary tumor

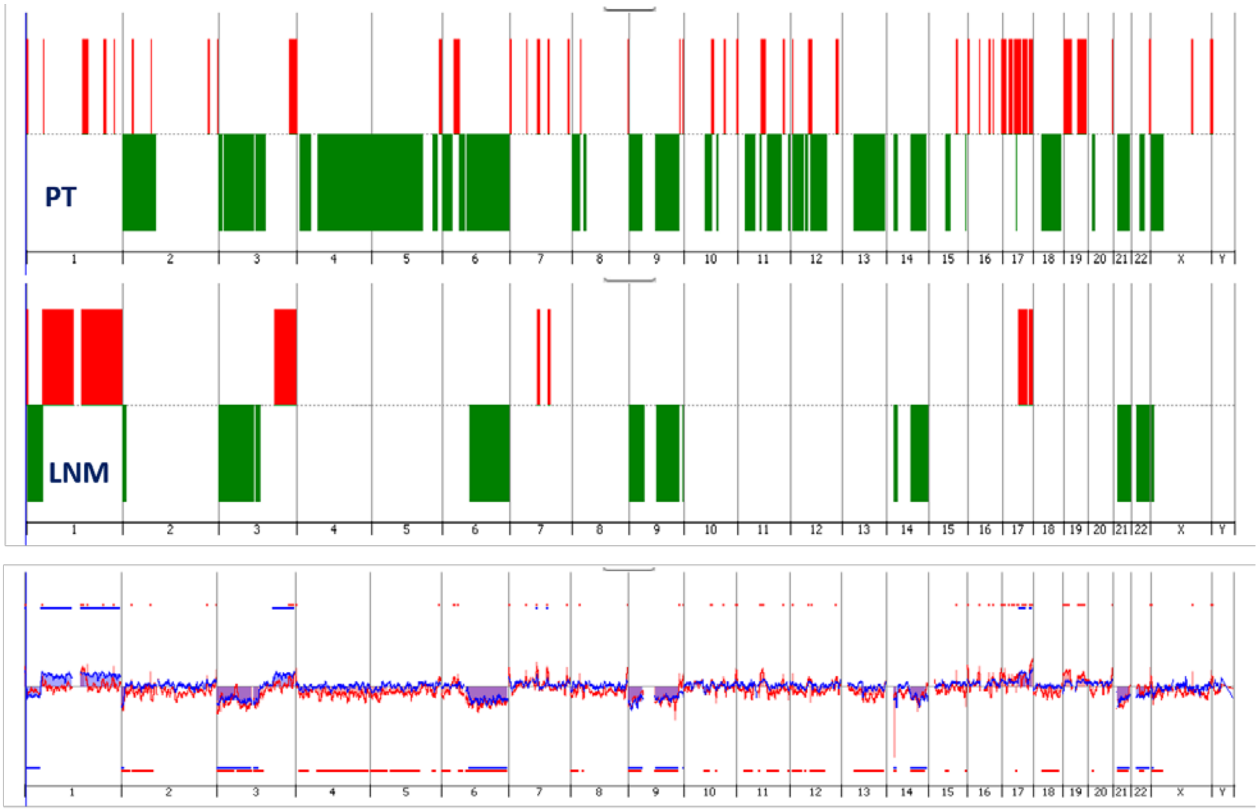

blue=LNM, red=primary tumor

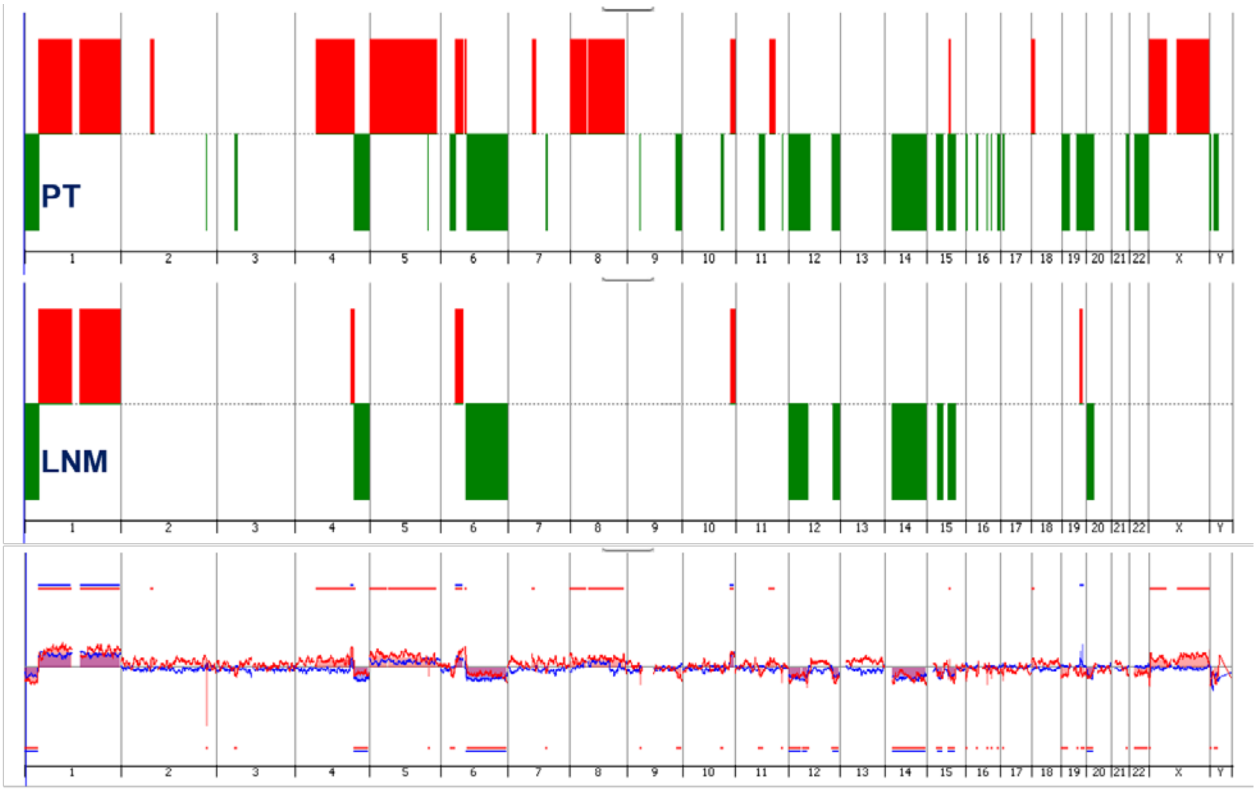

blue=LNM, red=primary tumor

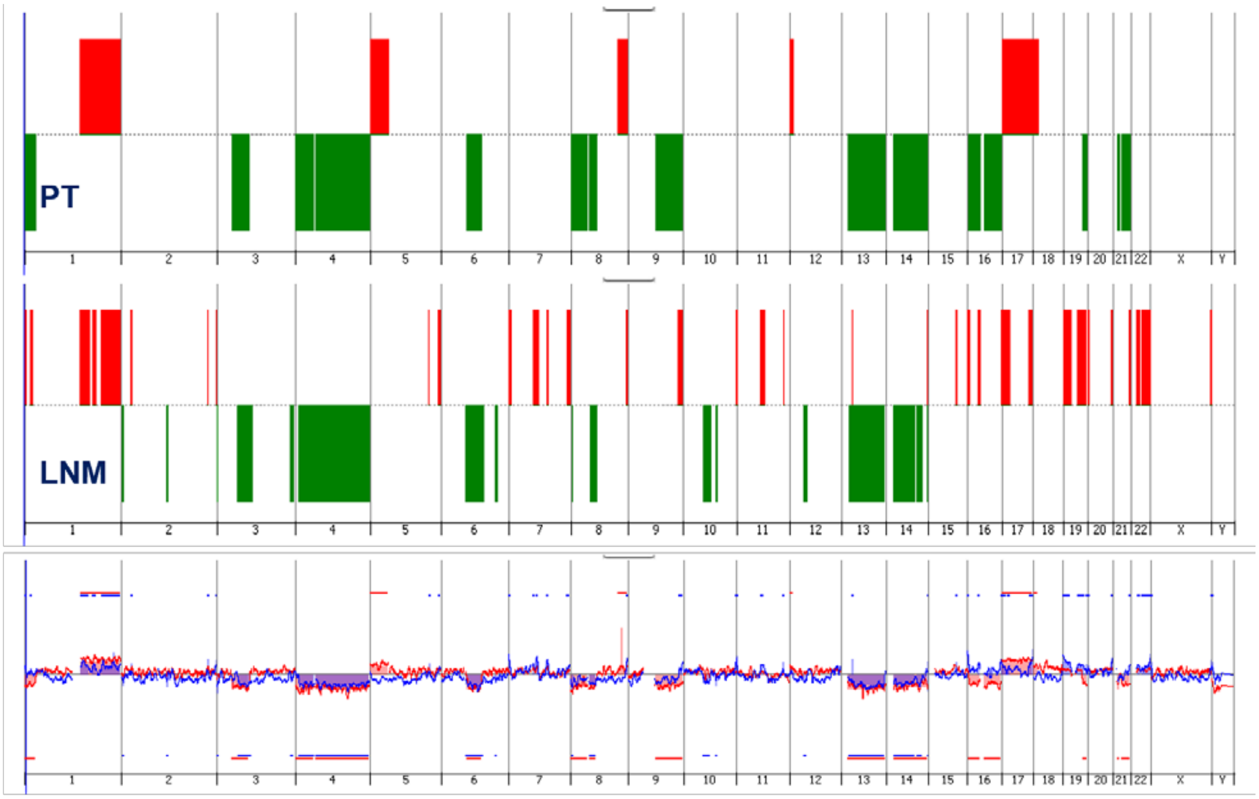

blue=LNM, red=primary tumor

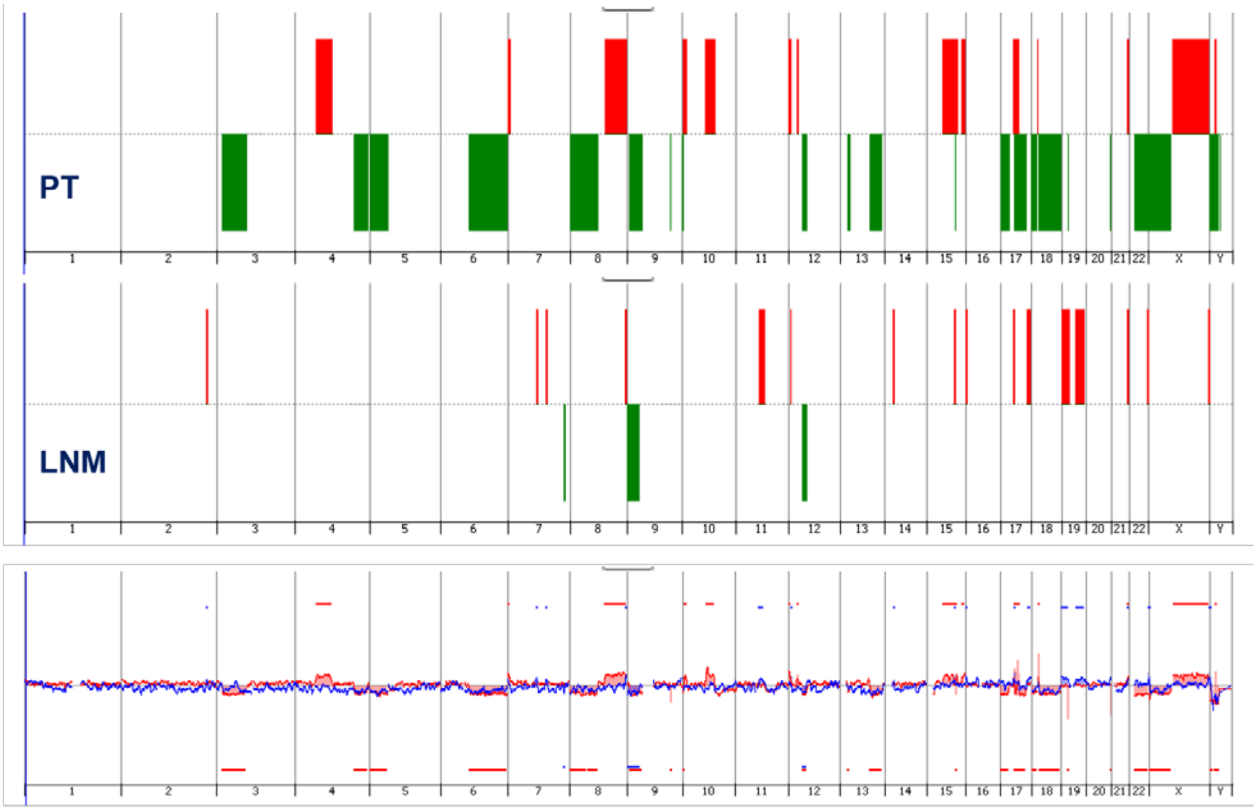

blue=LNM, red=primary tumor

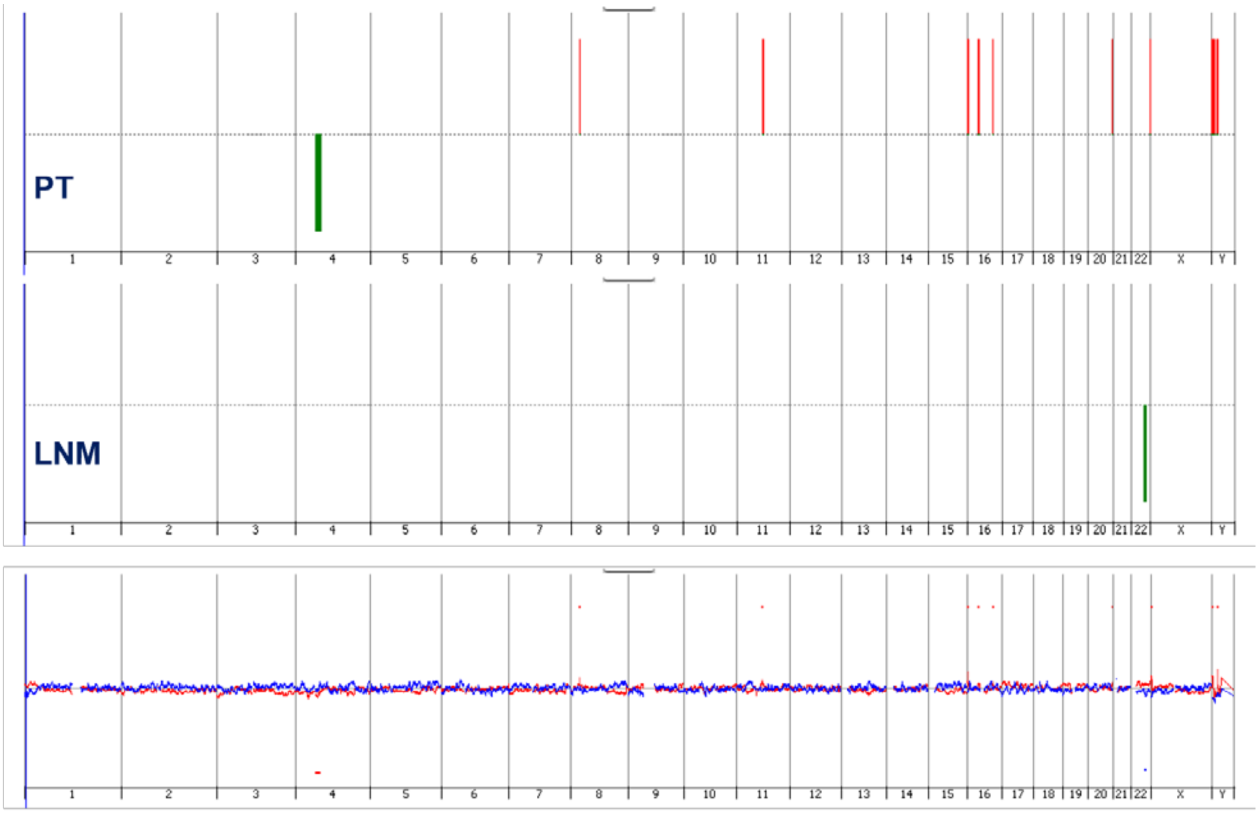

blue=LNM, red=primary tumor

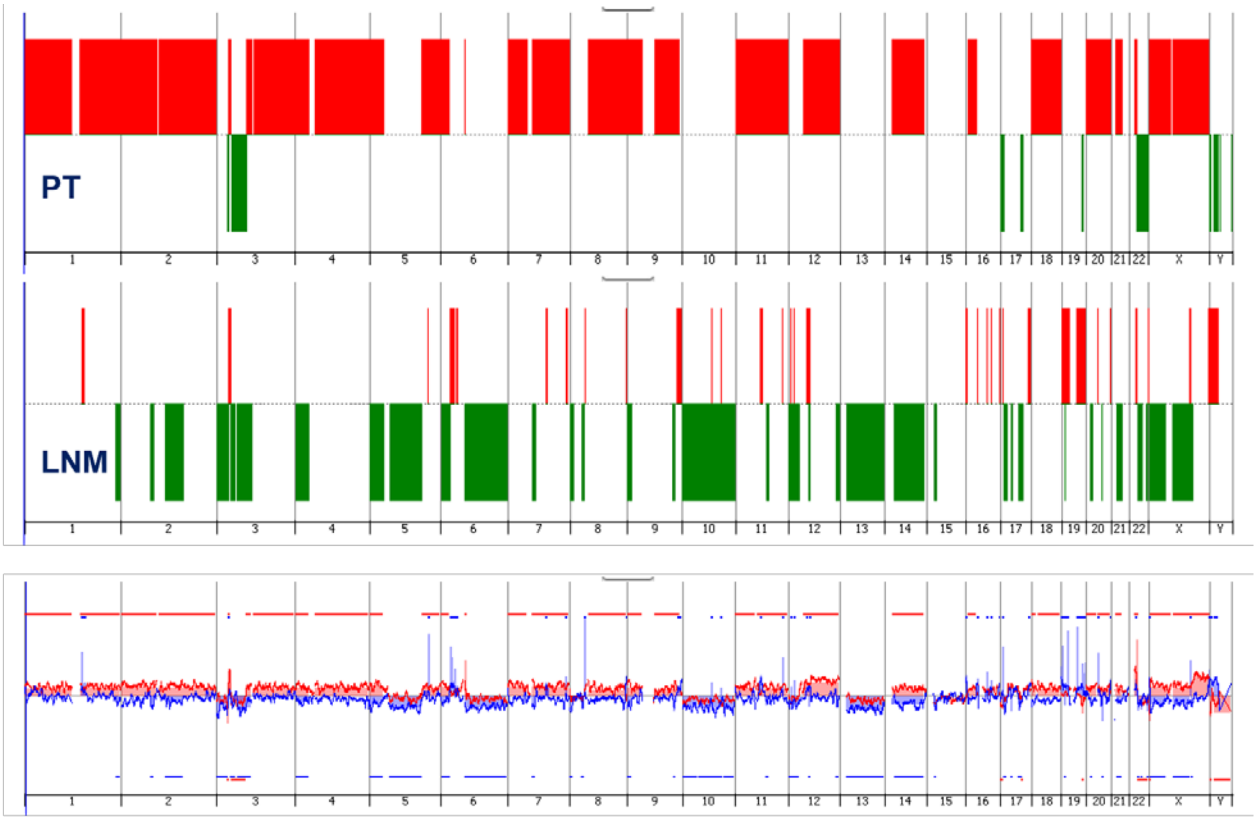

blue=primary tumor, red=LNM. **CAVE: inverted labeling**

Suppl. Fig. 4

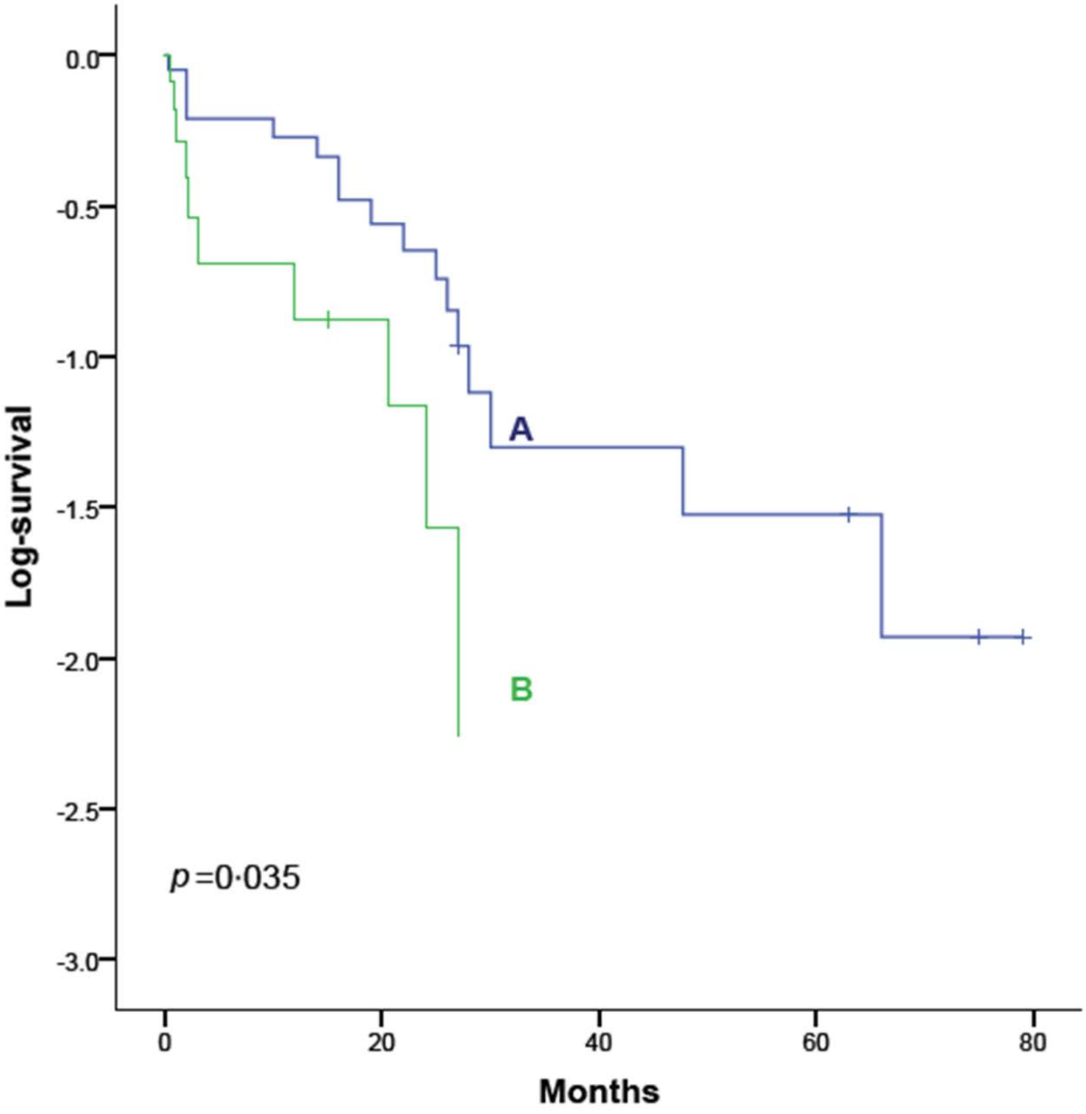

Suppl. Fig. 5

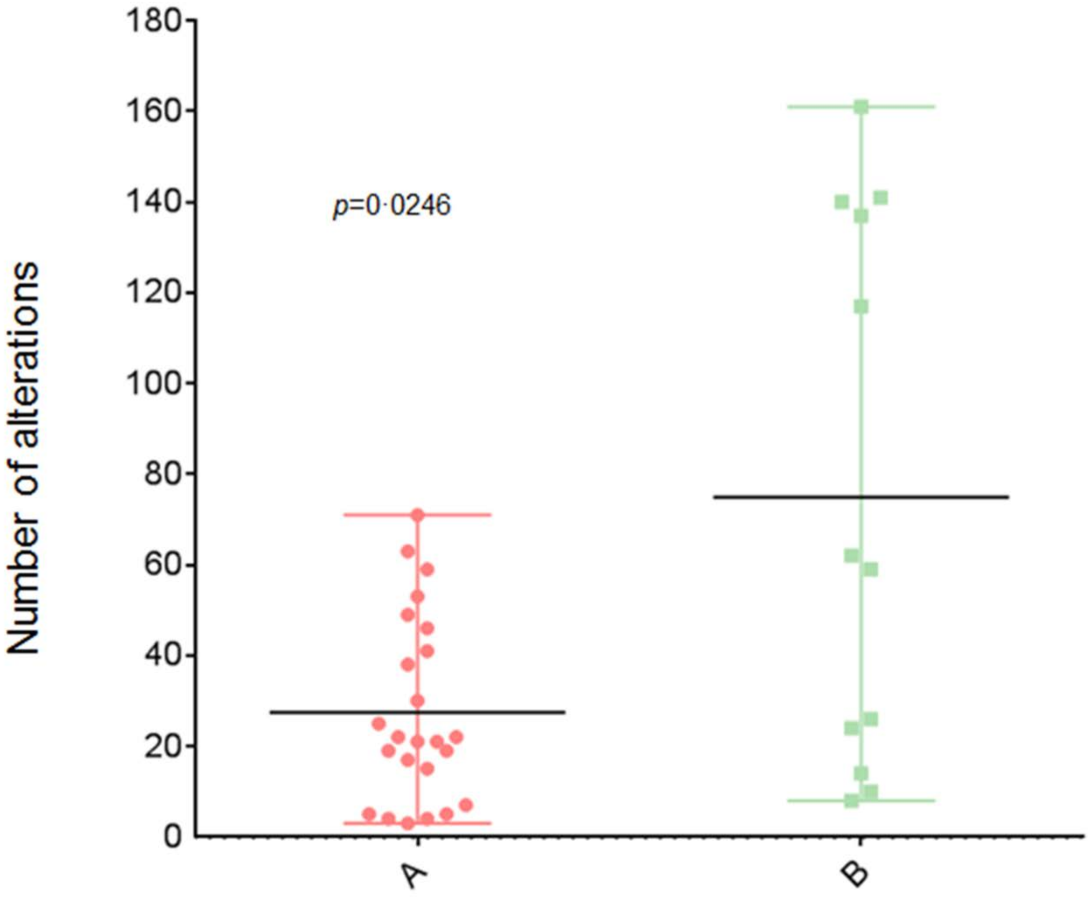

Number of alterations
180
160
140
120
100
80
60
40
20
0
p=0·0246
A
B

Suppl. Fig. 6

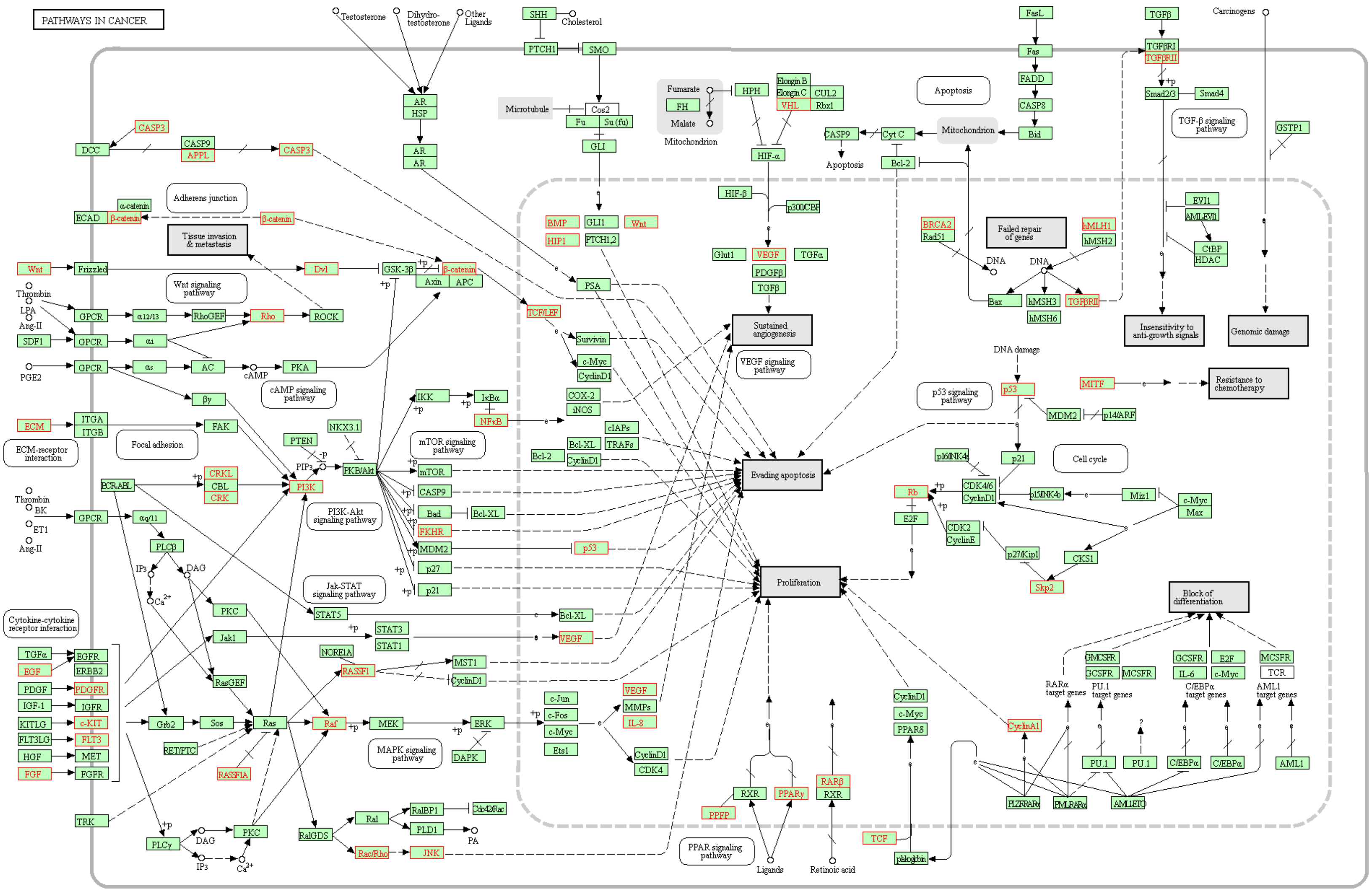

PATHWAYS IN CANCER
Testosterone
Dihydro-testosterone
Other Ligands
SHH
Cholesterol
PTCH1
SMO
AR
HSP
Microtubule
Cos2
Fu
Su (fu)
GLI
Fumarate
FH
Malate
Mitochondrion
HPH
Elongin B
Elongin C
CUL2
VHL
Rbx1
HIF-α
HIF-β
p300/CBP
Apoptosis
FasL
Fas
FADD
CASP8
Bid
Mitochondrion
CASP9
Cyt C
Bcl-2
TGFβ
TGFβRI
TGFβRII
Smad2/3
Smad4
TGF-β signaling pathway
Carcinogens
GSTP1
DCC
CASP3
CASP9
APPL
ECAD
α-catenin
β-catenin
Adherens junction
Tissue invasion & metastasis
Wnt
Frizzled
Dvl
GSK-3β
Axin
APC
Wnt signaling pathway
Thrombin
LPA
Ang-II
SDF1
GPCR
α12/13
RhoGEF
Rho
ROCK
αi
PGE2
αs
AC
cAMP
PKA
βγ
cAMP signaling pathway
ECM
ITGA
ITGB
FAK
ECM-receptor interaction
Focal adhesion
CRKL
CBL
CRK
PI3K
PTEN
PIP3
NKX3.1
PKB/Akt
IKK
IκBα
NFκB
mTOR signaling pathway
mTOR
CASP9
Bad
Bcl-XL
FKHR
MDM2
p53
p27
p21
PI3K-Akt signaling pathway
BCR-ABL
BK
ET1
αq/11
PLCβ
IP3
DAG
Ca2+
PKC
Jak1
STAT5
STAT3
STAT1
Jak-STAT signaling pathway
Cytokine-cytokine receptor interaction
TGFα
EGF
PDGF
IGF-1
KITLG
FLT3LG
HGF
FGF
EGFR
ERBB2
PDGFR
IGFR
c-KIT
FLT3
MET
FGFR
TRK
RasGEF
Grb2
Sos
Ras
RET/PTC
RASSF1A
NORE1A
RASSF1
MST1
CyclinD1
Raf
MEK
ERK
DAPK
MAPK signaling pathway
PLCγ
PKC
RalGDS
Ral
RalBP1
Cdc42/Rac
PLD1
PA
Rac/Rho
JNK
BMP
GLI1
Wnt
HIP1
PTCH1,2
PSA
TCF/LEF
Survivin
c-Myc
COX-2
iNOS
cIAPs
TRAFs
Bcl-2
Bcl-XL
Glut1
VEGF
TGFα
PDGFβ
TGFβ
Sustained angiogenesis
VEGF signaling pathway
Evading apoptosis
Proliferation
c-Jun
c-Fos
Ets1
MMPs
IL-8
CDK4
RXR
PPARγ
PPARδ
RARβ
PPFP
PPAR signaling pathway
Ligands
Retinoic acid
TCF
β-catenin
BRCA2
Rad51
Failed repair of genes
hMLH1
hMSH2
DNA
Bax
hMSH3
hMSH6
EVI1
AML-EVI1
CtBP
HDAC
Insensitivity to anti-growth signals
Genomic damage
DNA damage
MITF
Resistance to chemotherapy
p53 signaling pathway
MDM2
p14ARF
p16INK4a
p21
Cell cycle
Rb
E2F
CDK4/6
CDK2
CyclinE
p15INK4b
Miz1
Max
p27Kip1
CKS1
Skp2
Block of differentiation
GMCSFR
GCSFR
MCSFR
GCSFR
E2F
IL-6
TCR
RARα target genes
PU.1 target genes
C/EBPα target genes
AML1 target genes
PU.1
C/EBPα
AML1
CyclinA1
PLZF-RARα
PML-RARα
AML1-ETO

Suppl. Fig. 7

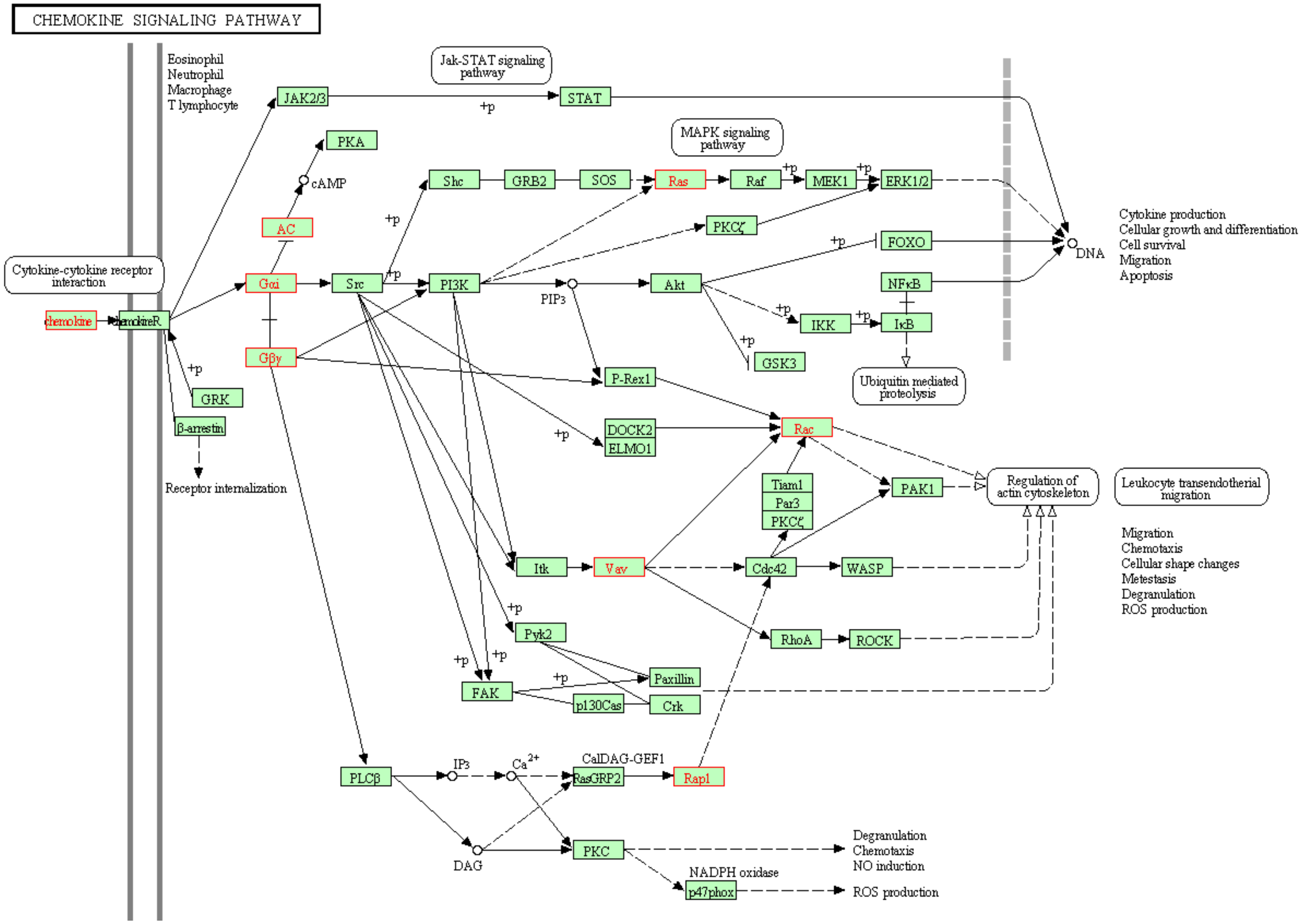

CHEMOKINE SIGNALING PATHWAY
Eosinophil
Neutrophil
Macrophage
T lymphocyte
Jak-STAT signaling pathway
JAK2/3
STAT
+p
PKA
cAMP
MAPK signaling pathway
Shc
GRB2
SOS
Ras
Raf
MEK1
ERK1/2
AC
PKCζ
FOXO
DNA
Cytokine production
Cellular growth and differentiation
Cell survival
Migration
Apoptosis
Cytokine-cytokine receptor interaction
Gαi
Src
PI3K
PIP3
Akt
NFκB
IKK
IκB
GSK3
chemokine
chemokineR
Gβγ
GRK
β-arrestin
Receptor internalization
P-Rex1
Ubiquitin mediated proteolysis
DOCK2
ELMO1
Rac
Tiam1
Par3
PKCζ
PAK1
Regulation of actin cytoskeleton
Leukocyte transendothelial migration
Migration
Chemotaxis
Cellular shape changes
Metestasis
Degranulation
ROS production
Itk
Vav
Cdc42
WASP
RhoA
ROCK
Pyk2
Paxillin
FAK
p130Cas
Crk
PLCβ
IP3
Ca2+
CalDAG-GEF1
RasGRP2
Rap1
DAG
PKC
Degranulation
Chemotaxis
NO induction
NADPH oxidase
p47phox
ROS production

Suppl. Fig. 1. Tumor size worsens survival in iCCA.

Kaplan-Meier estimator shows worse outcome for patients with a big tumor size (T3+4) compared to a smaller tumor size (T1+2). However, this result was not significant, but a trend could be shown (p=0.061).

Suppl. Fig. 2. Copy number alterations in N0 and N1 primary tumors and lymph node metastases.

N0, primary tumors without lymph node metastases; N1, primary tumors with lymph node metastases; LNM, lymph node metastases themselves. The minimal, maximal and mean amount of CNAs are displayed. Each point represents one sample.

Suppl. Fig 3. Patient-specific CGH profiles of all primary tumors and their matched LNM

Patient-specific CGH-profiles of each matched PT = primary tumor and its corresponding LNM = lymph node metastasis. (Blue = PT, red = LNM).

Suppl. Fig 4. Clustergroup B is associated with worse survival.

A significant shorter survival (13m vs. 31m, *p*=0·035) was found in the group with poorer tumor differentiation (Cluster B).

Suppl. Fig. 5. Copy number alterations cluster group A and B.

A, cluster group A; B, cluster group B (LNM independent, low grading and poor prognosis group). The amount of CNAs is significantly (p=0·0246) higher in cluster B. The minimal, maximal and mean amount of CNAs are displayed. Each point represents one sample.

Suppl. Fig. 6. Cancer associated pathways in N1 primary tumors.

Additional gains and losses of N1 primary tumors compared to N0 primary tumors are involved in cancer associated pathways. Involved genes are highlighted in red color. Pathway analyses were performed using KEGG Analysis, map05200, pathways in cancer, www.kegg.jp/kegg/kegg1.html. [1]

Suppl. Fig. 7. Chemokine signaling pathways in isolated LNM gains.

Isolated LNM gains harbor genes involved in chemokine signaling pathways. Involved genes are highlighted in red color. Pathway analyses were performed using KEGG Analysis, map04062 chemokine signaling pathway, www.kegg.jp/kegg/kegg1.html. [1]

Reference List Supplementary Information

1 Kanehisa, M., Furumichi, M., Tanabe, M., Sato, Y. & Morishima, K. KEGG: new perspectives on genomes, pathways, diseases and drugs. *Nucleic Acids Res* **45**, D353-D361, doi:10.1093/nar/gkw1092 (2017).

Supplement Table 1. Patients´ clinical characteristics.

Features	Total (n=60)	N0 (n=31)	N1 (n=23)	*p*-Value
Gender				
Male	31 (52%)	19 (61%)	9 (39%)	
Female	29 (48%)	12 (39%)	14 (61%)	n.s.
**Age (yrs)**				
Median	65 (15-81)	65	66	n.s.
**Etiology**				
Diabetes	10 (17%)	6 (19%)	1 (4%)	n.s.
Smoker	11 (18%)	2 (6%)	7 (30%)	0·008
Alcohol abuse	4 (7%)	3 (10%)	0	n.s.
Liver cirrhosis (due to)	14 (23%)	10 (32%)	1 (4%)	0·012
Alcohol	2 (14%)	1 (10%)	0	n.s.
Hepatitis A	1 (7%)	0	1 (100%)	n.s.
Hepatitis B	2 (14%)	1 (10%)	0	n.s.
Hepatitis C	3 (21%)	3 (30%)	0	n.s.
Steatosis hepatis	5 (36%)	4 (40%)	1 (100%)	n.s.
Hepatitis B	3 (5%)	1 (3%)	1 (4%)	n.s.
Hepatitis C	4 (7%)	3 (10%)	1 (4%)	n.s.
**Tumor markers**				
CA 19-9 (U/ml)	869 (0·6-10000)	136 (n=20)	2160 (n=12)	0·028

N0, primary tumors without lymph node metastases; N1, primary tumors with lymph node metastases; n.s., not significant.

Supplement Table 2. Resection modus according to nodal status.

	Total	N0	N1	*p*-Value
**Surgery**	**60**	**31**	**23**	
Right hepatectomy	17	12	3	n.s.
Left hepatectomy	20	12	7	n.s.
Extended right hepatectomy	5	2	2	n.s.
Extended left hepatectomy	4	0	3	n.s.
Central hepatectomy	4	3	2	n.s.
Trisegmentecetomy	8	2	6	n.s.

No significant correlation of the resection modus to the nodal status, survival or other analyzed factors was detected.

Suppl. Table 3. Median survival and recurrence of the cohort.

Features	Total	N0	N1	*p*-Value
Median Survival (m)	21 (0-79)	26 (2-79)	18 (0-64)	0·049
Median Recurrence (m)	12 (2-40)	14 (2-40)	13 (2-19)	n.s.
Time to Metastasis (m)	16 (0-41)	17	8	n.s.
Local Recurrence	8	4	4	n.s.
Distant Metastasis	16	5	11	0·012

The median survival for patients with N1 tumors was 18 months compared to 26 months for patients with a negative lymph node status. The medium survival was 21 months. Mean time for recurrence was 12 months after resection and no significant difference between N0 and N1 tumors were found.

Suppl. Table 4. Genes located on chromosomes with significant gains and losses in N1 primary tumors compared to N0 primary tumors.

Gains				Losses	
**3p**	**4q**	**5p**	**13q**	**17p**	**20p**
CHL1	DCUN1D4	PLEKHG4B	DKFZp686A1627	CRK	CSNK2A1
VHL	LRRC66	RICTOR	FGF9	FGF11	PLCB1
PPARG	SGCB	PRKAA1	FLT3	TP53	PLCB4
RAF1	SPATA18	PRLR	BRCA2	MAP2K4	SOX12
WNT7A	USP46	IL7R	CCNA1	MAPK7	TRIB3
RARB	RASL11B	LIFR	FOXO1	MAP2K3	TBC1D20
TGFBR2	SCFD2	OSMR	RB1	RPH3AL	ANGPT4
MLH1	FIP1L1	GHR	FGF14	C17orf97	RSPO4
CTNNB1	LNX1	CCDC127	COL4A1	VPS53	PSMF1
LAMB2	CHIC2	PDCD6	COL4A2	FAM57A	C20orf46
RHOA	GSX2	AHRR	TUBA3C	GLOD4	RAD21L1
RASSF1	PDGFRA	EXOC3	LOC100101938	NXN	SNPH
WNT5A	KIT	ZDHHC11	TPTE2	ABR	SDCBP2
APPL1	KDR	BRD9	MPHOSPH8	TUSC5	FKBP1A
MITF	SRD5A3	NKD2	PSPC1	YWHAE	NSFL1C
CNTN6	TMEM165	SLC12A7	ZMYM5	MYO1C	SIRPD
CNTN4	CLOCK	SLC6A18	ZMYM2	INPP5K	SIRPB1
IL5RA	PDCL2	TERT	GJA3	PITPNA	SIRPG
CRBN	NMU	CLPTM1L	GJB2	SLC43A2	SIRPA
LRRN1	LOC644145	SLC6A3	GJB6	SCARF1	PDYN
SETMAR	EXOC1	LPCAT1	CRYL1	RILP	STK35
SUMF1	CEP135	SDHAP3	IFT88	PRPF8	TGM3
ITPR1	KIAA1211	MRPL36	IL17D	C17orf91	TGM6
EGO	AASDH	IRX4	N6AMT2	SERPINF2	SNRPB
ARL8B	PPAT	C5orf38	XPO4	SERPINF1	ZNF343
GRM7	PAICS	LOC340094	LATS2	SMYD4	TMC2
LMCD1	SRP72	ADAMTS16	SAP18	RPA1	NOP56
LOH3CR2A	HOPX	KIAA0947	C13orf3	RTN4RL1	EBF4
C3orf32	SPINK2	NSUN2	MRP63	DPH1	CPXM1
CAV3	REST	SRD5A1	ZDHHC20	SMG6	C20orf141
RAD18	C4orf14	POLS	EFHA1	SRR	FAM113A
SRGAP3	POLR2B	ADCY2	SGCG	TSR1	VPS16
THUMPD3	IGFBP7	MTRR	SACS	SGSM2	PTPRA
SETD5	LPHN3	SEMA5A	TNFRSF19	MNT	GNRH2
LHFPL4	SRD5A2L2	LOC285692	MIPEP	METT10D	MRPS26
MTMR14	EPHA5	CMBL	SPATA13	PAFAH1B1	OXT
ARPC4	CENPC1	MARCH6	PARP4	KIAA0664	AVP

CRELD1	STAP1	ROPN1L	RNF17	GARNL4	UBOX5
TMEM111	UBA6	DAP	CENPJ	OR1D2	FASTKD5
FANCD2	GNRHR	CTNND2	LOC646405	OR1G1	DDRGK1
C3orf24	TMPRSS11D	DNAH5	FAM123A	OR3A1	ITPA
C3orf10	TMPRSS11A	TRIO	MTMR6	OR1E2	SLC4A11
IRAK2	TMPRSS11F	FAM105A	NUPL1	SPATA22	C20orf194
TATDN2	LOC100130017	FAM105B	ATP8A2	ASPA	ATRN
C3orf42	FLJ41562	ANKH	RNF6	TRPV3	GFRA4
GHRLOS	TMPRSS11B	FBXL7	CDK8	TRPV1	ADAM33
GHRL	YTHDC1	MARCH11	WASF3	SHPK	SIGLEC1
SEC13	UGT2A3	ZNF622	GPR12	CTNS	HSPA12B
ATP2B2	UGT2B11	FAM134B	USP12	TMEM93	C20orf27
SLC6A11	UGT2B4	MYO10	RPL21P28	P2RX5	SPEF1
SLC6A1	UGT2A1	LOC285696	SNORD102	GSG2	CENPB
HRH1	UGT2A2	BASP1	RPL21	ITGAE	CDC25B
ATG7	SULT1B1	CDH18	GTF3A	C17orf85	C20orf29
VGLL4	SULT1E1	CDH12	MTIF3	CAMKK1	PANK2
C3orf31	CSN1S1	PRDM9	LNX2	P2RX1	RNF24
SYN2	CSN2	CDH10	POLR1D	ATP2A3	SMOX
TIMP4	STATH	CDH9	PDX1	ZZEF1	ADRA1D
TSEN2	HTN3	CDH6	CDX2	CYB5D2	PRNP
MKRN2	HTN1	RNASEN	PAN3	ANKFY1	PRND
TMEM40	CSN1S2B	C5orf22	FLT1	UBE2G1	PRNT
CAND2	ODAM	PDZD2	POMP	SPNS3	RASSF2
IQSEC1	C4orf7	GOLPH3	SLC46A3	SPNS2	SLC23A2
NUP210	CSN3	MTMR12	KIAA0774	MYBBP1A	C20orf30
FBLN2	C4orf35	ZFR	SLC7A1	GGT6	PCNA
LOC285375	SMR3A	SUB1	UBL3	ALOX15	CDS2
TPRXL	SMR3B	NPR3	KATNAL1	PELP1	PROKR2
CHCHD4	PROL1	C5orf23	LOC100188949	ARRB2	RP5-1022P6.2
TMEM43	MUC7	TARS	HMGB1	CXCL16	C20orf196
XPC	AMBN	ADAMTS12	USPL1	TM4SF5	CHGB
LSM3	ENAM	SLC45A2	ALOX5AP	VMO1	TRMT6
SLC6A6	IGJ	AMACR	C13orf33	PSMB6	MCM8
GRIP2	UTP3	C1QTNF3	C13orf26	PLD2	CRLS1
C3orf19	RUFY3	RAI14	HSPH1	MINK1	LRRN4
C3orf20	GRSF1	TTC23L	B3GALTL	CHRNE	FERMT1
FGD5	MOBKL1A	RAD1	RXFP2	LOC100130311	BMP2
NR2C2	DCK	BXDC2	EEF1DP3	GP1BA	HAO1
MRPS25	SLC4A4	DNAJC21	FRY	SLC25A11	TMX4
ZFYVE20	GC	AGXT2	N4BP2L1	RNF167	C20orf103
CAPN7	NPFFR2	SPEF2	N4BP2L2	PFN1	PAK7

SH3BP5	ADAMTS3	CAPSL	PDS5B	ENO3	ANKRD5
METTL6	COX18	UGT3A1	KL	SPAG7	SNAP25
EAF1	ANKRD17	UGT3A2	STARD13	CAMTA2	MKKS
COLQ	ALB	LMBRD2	RFC3	INCA1	C20orf94
HACL1	AFP	SKP2	NBEA	KIF1C	JAG1
BTD	AFM	C5orf33	MAB21L1	GPR172B	BTBD3
ANKRD28	RASSF6	RANBP3L	DCLK1	ZFP3	SPTLC3
OXNAD1	IL8	SLC1A3	SOHLH2	ZNF232	ISM1
RFTN1	CXCL6	NIPBL	C13orf38	ZNF594	TASP1
DAZL	PF4	C5orf42	SPG20	C17orf87	ESF1
PLCL2	PPBP	NUP155	RFXAP	RABEP1	C20orf7
TBC1D5	CXCL5	WDR70	SMAD9	NUP88	SEL1L2
SATB1	CXCL3	GDNF	ALG5	RPAIN	MACROD2
KCNH8	PPBPL2	EGFLAM	EXOSC8	C1QBP	FLRT3
EFHB	CXCL2	FYB	FAM48A	DHX33	KIF16B
RAB5A	MTHFD2L	C9	CSNK1A1L	DERL2	SNRPB2
KAT2B	EREG	DAB2	POSTN	MIS12	OTOR
SGOL1	BTC	PTGER4	TRPC4	NLRP1	PCSK2
ZNF385D	DKFZP564O0823	TTC33	UFM1	WSCD1	BFSP1
UBE2E2	RCHY1	RPL37	FREM2	AIPL1	DSTN
UBE2E1	THAP6	CARD6	STOML3	FAM64A	RRBP1
NKIRAS1	C4orf26	C7	C13orf23	PITPNM3	BANF2
RPL15	CDKL2	HEATR7B2	LHFP	KIAA0753	C20orf72
NR1D2	G3BP2	C6	COG6	TXNDC17	OVOL2
THRB	USO1	PLCXD3	LOC646982	MED31	CSRP2BP
TOP2B	PPEF2	OXCT1	MRPS31	SLC13A5	ZNF133
NGLY1	NAAA	C5orf51	SLC25A15	XAF1	FOXA2
OXSM	SDAD1	FBXO4	SUGT1L1	FBXO39	THBD
LRRC3B	CXCL9	SEPP1	ELF1	TEKT1	CD93
NEK10	CXCL10	ZNF131	WBP4	ALOX12	NXT1
SLC4A7	CXCL11	MGC42105	KBTBD6	C17orf49	GZF1
EOMES	ART3	HMGCS1	KBTBD7	BCL6B	NAPB
CMC1	NUP54	CCL28	MTRF1	SLC16A11	CSTL1
AZI2	SCARB2	C5orf28	NARG1L	CLEC10A	CST11
ZCWPW2	FAM47E	PAIP1	C13orf15	ASGR2	CST8
RBMS3	STBD1	NNT	KIAA0564	ASGR1	LOC164380
GADL1	CCDC158	FGF10	DGKH	DLG4	CST9L
STT3B	SHROOM3	MRPS30	AKAP11	ACADVL	CST3
OSBPL10	CCNI	HCN1	TNFSF11	DVL2	CST5
GPD1L	CCNG2		C13orf30	PHF23	C20orf39
CMTM8	CXCL13		EPSTI1	GABARAP	CST7
CMTM7	CNOT6L		DNAJC15	DULLARD	C20orf3

CMTM6	MRPL1		ENOX1	C17orf81	ACSS1
DYNC1LI1	FRAS1		CCDC122	SLC2A4	VSX1
CNOT10	ANXA3		C13orf31	EIF5A	LOC284798
TRIM71	BMP2K		LOC121838	GPS2	ENTPD6
CCR4	PAQR3		SERP2	NEURL4	PYGB
GLB1	ARD1B		TSC22D1	ACAP1	ABHD12
TMPPE	GK2		NUFIP1	TNK1	GINS1
CRTAP	GDEP		KIAA1704	PLSCR3	NLP
SUSD5	ANTXR2		GTF2F2	C17orf61	NANP
FBXL2	PRDM8		TPT1	NLGN2	ZNF337
UBP1	FGF5		LOC100190939	SPEM1	
CLASP2	C4orf22		SLC25A30	C17orf74	
PDCD6IP	BMP3		COG3	TMEM102	
ARPP-21	PRKG2		FLJ32682	CHRNB1	
STAC	RASGEF1B		SPERT	ZBTB4	
DCLK3	HNRNPD		SIAH3	POLR2A	
LBA1	HNRPDL		ZC3H13	TNFSF12	
EPM2AIP1	ENOPH1		CPB2	TNFSF12-TNFSF13	
LRRFIP2	FLJ12993		LCP1	SENP3	
GOLGA4	SCD5		C13orf18	EIF4A1	
ITGA9	SEC31A		LRCH1	MPDU1	
CTDSPL	THAP9		ESD	SOX15	
VILL	LIN54		HTR2A	FXR2	
PLCD1	COPS4		SUCLA2	SAT2	
DLEC1	PLAC8		NUDT15	SHBG	
ACAA1	COQ2		MED4	ATP1B2	
MYD88	HPSE		ITM2B	WRAP53	
OXSR1	HELQ		RB1	EFNB3	
SLC22A13	MRPS18C		RCBTB2	DNAH2	
SLC22A14	FAM175A		CYSLTR2	LSMD1	
XYLB	AGPAT9		FNDC3A	CYB5D1	
ACVR2B	NKX6-1		MLNR	CHD3	
EXOG	CDS1		CDADC1	KCNAB3	
SCN5A	WDFY3		CAB39L	TRAPPC1	
SCN10A	ARHGAP24		SETDB2	CNTROB	
SCN11A	MAPK10		PHF11	GUCY2D	
WDR48	PTPN13		RCBTB1	ALOX15B	
GORASP1	SLC10A6		ARL11	ALOX12B	
TTC21A	C4orf36		EBPL	HES7	
CSRNP1	AFF1		KPNA3	PER1	
XIRP1	KLHL8		C13orf1	VAMP2	
CX3CR1	HSD17B13		TRIM13	TMEM107	

CCR8	HSD17B11		KCNRG	C17orf59	
SLC25A38	NUDT9		DLEU2	AURKB	
RPSA	SPARCL1		DLEU1	C17orf44	
MOBP	DSPP		DLEU7	C17orf68	
MYRIP	DMP1		RNASEH2B	PFAS	
EIF1B	IBSP		GUCY1B2	SLC25A35	
ENTPD3	MEPE		FAM124A	RANGRF	
RPL14	SPP1		SERPINE3	ARHGEF15	
ZNF619	PKD2		INTS6	ODF4	
ZNF621	ABCG2		WDFY2	LOC100128288	
ULK4	PPM1K		DHRS12	KRBA2	
TRAK1	HERC6		FLJ37307	RPL26	
CCK	HERC5		CCDC70	NDEL1	
LYZL4	HERC3		ATP7B	MYH10	
SEC22C	NAP1L5		ALG11	CCDC42	
SS18L2	FAM13AOS		NEK5	SPDYE4	
NKTR	FAM13A		NEK3	MFSD6L	
ZBTB47	TIGD2		THSD1P	PIK3R6	
CCDC13	GPRIN3		THSD1	PIK3R5	
HIGD1A	SNCA		VPS36	NTN1	
CCBP2	MMRN1		CKAP2	STX8	
CYP8B1	KIAA1680		LOC220115	WDR16	
LOC729085	TMSL3		SUGT1	USP43	
C3orf39	GRID2		LECT1	DHRS7C	
SNRK	ATOH1		PCDH8	GLP2R	
ANO10	SMARCAD1		OLFM4	RCVRN	
ABHD5	PGDS		PCDH17	GAS7	
LOC375337	PDLIM5		DIAPH3	MYH13	
C3orf23	BMPR1B		TDRD3	MYH8	
ZNF445	UNC5C		PCDH20	MYH4	
ZNF167	PDHA2		PCDH9	MYH2	
ZNF660	C4orf37		KLHL1	MYH3	
ZNF197	RAP1GDS1		ATXN8OS	C17orf48	
ZNF35	TSPAN5		DACH1	FLJ45455	
ZNF502	EIF4E		C13orf37	DNAH9	
ZNF501	METAP1		C13orf34	ZNF18	
KIAA1143	ADH4		C13orf34	MYOCD	
KIF15	ADH6		DIS3	RICH2	
TMEM42	ADH1B		PIBF1	ELAC2	
TGM4	ADH1C		KLF5	HS3ST3A1	
ZDHHC3	ADH7		KLF12	COX10	
EXOSC7	C4orf17		TBC1D4	HS3ST3B1	

CLEC3B	RG9MTD2		COMMD6	PMP22	
CDCP1	MTTP		UCHL3	TEKT3	
TMEM158	DAPP1		LMO7	CDRT4	
LARS2	DNAJB14		KCTD12	FAM18B2	
LIMD1	H2AFZ		CLN5	CDRT1	
SACM1L	DDIT4L		FBXL3	TRIM16	
SLC6A20	EMCN		MYCBP2	ZNF286A	
LZTFL1	PPP3CA		SCEL	ADORA2B	
CCR9	BANK1		SLAIN1	TTC19	
FYCO1	SLC39A8		EDNRB	NCOR1	
CXCR6	NFKB1		POU4F1	PIGL	
XCR1	MANBA		RNF219	CENPV	
CCR1	UBE2D3		RBM26	TRPV2	
CCR3	CISD2		NDFIP2	C17orf45	
LTF	NHEDC1		SPRY2	C17orf76	
RTP3	NHEDC2		SLITRK6	ZNF287	
LRRC2	BDH2		GPC5	ZNF624	
TDGF1	CENPE		GPC6	TNFRSF13B	
ALS2CL	TACR3		DCT	MPRIP	
TMIE	CXXC4		TGDS	PLD6	
TSP50	TET2		GPR180	FLCN	
TESSP2	PPA2		SOX21	COPS3	
MYL3	FLJ20184		ABCC4	NT5M	
CCDC12	INTS12		CLDN10	MED9	
LOC100129354	GSTCD		DZIP1	RASD1	
SETD2	NPNT		DNAJC3	PEMT	
KIF9	TBCKL		UGCGL2	RAI1	
KLHL18	SCYE1		HS6ST3	SMCR5	
PTPN23	DKK2		OXGR1	SREBF1	
SCAP	PAPSS1		MBNL2	TOM1L2	
C3orf75	SGMS2		RAP2A	LRRC48	
CSPG5	CYP2U1		IPO5	ATPAF2	
SMARCC1	HADH		FARP1	C17orf39	
DHX30	LEF1		RNF113B	DRG2	
MAP4	LOC285456		STK24	MYO15A	
CDC25A	RPL34		SLC15A1	ALKBH5	
CAMP	OSTC		DOCK9	LLGL1	
ZNF589	AGXT2L1		UBAC2	FLII	
NME6	COL25A1		GPR18	SMCR7	
FBXW12	SEC24B		GPR183	TOP3A	
PLXNB1	CCDC109B		FKSG29	SHMT1	
CCDC51	CASP6		TM9SF2	FBXW10	

CCDC72	PLA2G12A		CLYBL	PRPSAP2	
ATRIP	CFI		ZIC5	SLC5A10	
SHISA5	GAR1		ZIC2	FAM83G	
PFKFB4	RRH		PCCA	GRAP	
UCN2	EGF		A2LD1	EPN2	
COL7A1	ELOVL6		TMTC4	B9D1	
UQCRC1	ENPEP		NALCN	MFAP4	
SLC26A6	PITX2		ITGBL1	RNF112	
CELSR3	C4orf32		TPP2	SLC47A1	
NCKIPSD	C4orf16		C13orf27	ALDH3A2	
IP6K2	TIFA		BIVM	SLC47A2	
PRKAR2A	ALPK1		ERCC5	ALDH3A1	
SLC25A20	NEUROG2		LOC121952	ULK2	
ARIH2	LOC91431		SLC10A2	AKAP10	
P4HTM	C4orf21		DAOA	CYTSB	
WDR6	LARP7		EFNB2	CCDC144NL	
DALRD3	ANK2		ARGLU1	USP22	
IMPDH2	CAMK2D		FAM155A	DHRS7B	
QRICH1	ARSJ		LIG4	TMEM11	
QARS	UGT8		ABHD13	C17orf103	
CCDC71	NDST4		TNFSF13B	KCNJ12	
KLHDC8B	TRAM1L1		MYO16	C17orf51	
CCDC36	NDST3		IRS2		
USP4	PRSS12		RAB20		
GPX1	CEP170L		CARKD		
TCTA	METTL14		CARS2		
AMT	SEC24D		ING1		
NICN1	SYNPO2		ANKRD10		
DAG1	MYOZ2		ARHGEF7		
BSN	USP53		C13orf16		
APEH	FABP2		SOX1		
RNF123	PDE5A		C13orf28		
GMPPB	MAD2L1		TUBGCP3		
IP6K1	PRDM5		C13orf35		
UBA7	C4orf31		ATP11A		
TRAIP	TNIP3		MCF2L		
CAMKV	QRFPR		F7		
MST1R	ANXA5		F10		
MON1A	TMEM155		PROZ		
RBM6	EXOSC9		PCID2		
RBM5	CCNA2		CUL4A		
SEMA3F	BBS7		LAMP1		

GNAT1	TRPC3		GRTP1		
SEMA3B	KIAA1109		ADPRHL1		
C3orf45	ADAD1		DCUN1D2		
HYAL1	IL2		TMCO3		
HYAL2	IL21		TFDP1		
TUSC2	BBS12		ATP4B		
ZMYND10	FGF2		GRK1		
TUSC4	NUDT6		GAS6		
CYB561D2	SPATA5		FAM70B		
TMEM115	LOC285419		RASA3		
CACNA2D2	ANKRD50		CDC16		
C3orf18	FAT4		UPF3A		
HEMK1	INTU				
CISH	SLC25A31				
MAPKAPK3	HSPA4L				
DOCK3	PLK4				
RBM15B	MFSD8				
VPRBP	C4orf29				
RAD54L2	LARP2				
TEX264	PGRMC2				
GRM2	PHF17				
IQCF2	SCLT1				
IQCF1	C4orf33				
RRP9	PCDH10				
PARP3	PCDH18				
GPR62	SLC7A11				
PCBP4	CCRN4L				
ABHD14B	ELF2				
ACY1	C4orf49				
RPL29	NDUFC1				
WDR51A	NARG1				
ALAS1	RAB33B				
TLR9	SETD7				
TWF2	MGST2				
PPM1M	MAML3				
WDR82	SCOC				
GLYCTK	CLGN				
DNAH1	ELMOD2				
BAP1	UCP1				
PHF7	TBC1D9				
SEMA3G	RNF150				
TNNC1	ZNF330				

NISCH	IL15				
STAB1	INPP4B				
NT5DC2	USP38				
PBRM1	GAB1				
GNL3	SMARCA5				
GLT8D1	GYPA				
SPCS1	HHIP				
NEK4	ANAPC10				
ITIH1	ABCE1				
ITIH3	OTUD4				
ITIH4	SMAD1				
TMEM110	MMAA				
SFMBT1	LOC646603				
RFT1	ZNF827				
PRKCD	SLC10A7				
TKT	POU4F2				
DCP1A	TTC29				
CACNA1D	EDNRA				
CHDH	TMEM184C				
IL17RB	LOC90826				
ACTR8	ARHGAP10				
SELK	NR3C2				
CACNA2D3	DCLK2				
LRTM1	LRBA				
ERC2	MAB21L2				
C3orf51	RPS3A				
CCDC66	SH3D19				
C3orf63	ESSPL				
ARHGEF3	FAM160A1				
SPATA12	PET112L				
IL17RD	FBXW7				
HESX1	TMEM154				
ASB14	TIGD4				
DNAH12	ARFIP1				
PDE12	FHDC1				
ARF4	TRIM2				
FAM116A	MND1				
SLMAP	KIAA0922				
FLNB	TLR2				
DNASE1L3	RNF175				
ABHD6	DCHS2				
RPP14	PLRG1				

PXK	FGB				
PDHB	FGA				
KCTD6	FGG				
ACOX2	LRAT				
FAM107A	RBM46				
FAM3D	NPY2R				
C3orf67	MAP9				
FHIT	GUCY1A3				
PTPRG	GUCY1B3				
C3orf14	ACCN5				
FEZF2	TDO2				
CADPS	CTSO				
SYNPR	PDGFC				
C3orf49	GLRB				
THOC7	GRIA2				
ATXN7	C4orf18				
PSMD6	TMEM144				
PRICKLE2	RXFP1				
ADAMTS9	ETFDH				
MAGI1	PPID				
SLC25A26	FNIP2				
LRIG1	C4orf45				
KBTBD8	RAPGEF2				
SUCLG2	FSTL5				
FAM19A1	NAF1				
FAM19A4	NPY1R				
C3orf64	NPY5R				
TMF1	TKTL2				
UBA3	MARCH1				
ARL6IP5	TRIM61				
LMOD3	TRIM60				
FRMD4B	TMEM192				
FOXP1	KLHL2				
EIF4E3	SC4MOL				
PROK2	CPE				
RYBP	TLL1				
SHQ1	SPOCK3				
GLT8D4	ANXA10				
PPP4R2	DDX60				
FLJ10213	DDX60L				
PDZRN3	PALLD				
CNTN3	CBR4				

ROBO2	SH3RF1				
ROBO1	NEK1				
GBE1	CLCN3				
CADM2	C4orf27				
VGLL3	MFAP3L				
CHMP2B	AADAT				
POU1F1	GALNTL6				
HTR1F	GALNT7				
CGGBP1	HMGB2				
C3orf38	SAP30				
EPHA3	SCRG1				
	HAND2				
	NBLA00301				
	FBXO8				
	KIAA1712				
	HPGD				
	GLRA3				
	ADAM29				
	GPM6A				
	WDR17				
	SPATA4				
	ASB5				
	VEGFC				
	NEIL3				
	AGA				
	LOC285501				
	MGC45800				
	ODZ3				
	DCTD				
	C4orf38				
	WWC2				
	CDKN2AIP				
	ING2				
	RWDD4A				
	C4orf41				
	STOX2				
	ENPP6				
	IRF2				
	CASP3				
	CCDC111				
	MLF1IP				
	ACSL1				

	SLC25A4				
	KIAA1430				
	SNX25				
	LRP2BP				
	ANKRD37				
	UFSP2				
	C4orf47				
	CCDC110				
	PDLIM3				
	SORBS2				
	TLR3				
	FAM149A				
	CYP4V2				
	KLKB1				
	F11				
	FAT1				
	ZFP42				
	TRIML2				
	TRIML1				

Suppl. Table 5. Genes located on chromosomes that showed isolated gains in the LNM of iCCA.

1p13	2p23	7p22	7q11	11q12	12q14	13q13	14q12
PRMT6	ATAD2B	FAM20C	ZNF679	APLNR	SLC26A10	RXFP2	LRRC16B
NTNG1	UBXN2A	PDGFA	ZNF680	TNKS1BP1	B4GALNT1	EEF1DP3	CPNE6
VAV3	C2orf44	PRKAR1B	ZNF107	SSRP1	OS9	FRY	NRL
SLC25A24	FKBP1B	HEATR2	ZNF138	P2RX3	AGAP2	BRCA2	PCK2
FAM102B	SF3B14	UNC84A	ZNF117	PRG3	LOC100130776	N4BP2L1	WDR23
C1orf59	TP53I3	C7orf20	INTS4L1	PRG2	TSPAN31	N4BP2L2	FAM158A
PRPF38B	LOC375190	C7orf50	CCT6P1	SLC43A3	CDK4	PDS5B	PSME2
FNDC7	ITSN2	GPER	VKORC1L1	RTN4RL2	MARCH9	KL	RNF31
STXBP3	NCOA1	ZFAND2A	GUSB	SLC43A1	METTL1	STARD13	IRF9
C1orf62	CENPO	INTS1	CRCP	TIMM10	FAM119B	RFC3	REC8
GPSM2	ADCY3	TMEM184A	TPST1	UBE2L6	TSFM	NBEA	IPO4
CLCC1	DNAJC27	MAFK	NCRNA00174	SERPING1	AVIL	MAB21L1	TM9SF1
WDR47	EFR3B	KIAA1908	LOC493754	YPEL4	CTDSP2	DCLK1	TSSK4
KIAA1324	POMC	MAD1L1	KCTD7	CLP1	XRCC6BP1	SOHLH2	CHMP4A
SARS	DNMT3A	FTSJ2	RABGEF1	ZDHHC5	LRIG3	C13orf38	MDP-1
CELSR2	DTNB	NUDT1	C7orf42	MED19	SLC16A7	SPG20	NEDD8
PSRC1	ASXL2	EIF3B	TYW1	TMX2	FAM19A2	CCNA1	GMPR2
SORT1	KIF3C	CHST12	AUTS2	C11orf31	USP15	RFXAP	TINF2
PSMA5	RAB10	IQCE	WBSCR17	CTNND1	MON2	SMAD9	TGM1
SYPL2	HADHA	TTYH3	CALN1	OR9Q1	C12orf61	ALG5	RABGGTA
CYB561D1	HADHB	GNA12	TYW1B	OR10W1	PPM1H	EXOSC8	DHRS1
GPR61	GPR113	CARD11	POM121	LPXN	AVPR1A	FAM48A	C14orf21
GNAI3	SELI	SDK1	TRIM50	ZFP91	DPY19L2	CSNK1A1L	LTB4R2
GNAT2	C2orf39	FOXK1	FKBP6	CNTF	TMEM5	POSTN	CIDEB
AMPD2	OTOF	RADIL	BAZ1B	ZFP91-CNTF	SRGAP1	TRPC4	LTB4R
GSTM4	CIB4	PAPOLB	TBL2	GLYAT	C12orf66	UFM1	ADCY4

GSTM5	KCNK3	MMD2	MLXIPL	GLYATL1	C12orf56	FREM2	NFATC4
GSTM3	C2orf18	RBAK	DNAJC30	FAM111B	XPOT	STOML3	KIAA1305
EPS8L3	CENPA	WIPI2	WBSCR22	FAM111A	TBK1	C13orf23	SDR39U1
CSF1	DPYSL5	TNRC18	STX1A	DTX4	RASSF3	LHFP	KIAA0323
AHCYL1	MAPRE3	FBXL18	ABHD11	OSBP	GNS	COG6	CMA1
ALX3	TMEM214	RNF216	CLDN3	PATL1	WIF1		CTSG
SLC6A17	AGBL5	ZNF815	CLDN4	STX3	LEMD3		GZMH
KCNC4	KHK	OCM	WBSCR27	MRPL16	MSRB3		GZMB
RBM15	CGREF1	PMS2	WBSCR28	GIF	RPSAP52		STXBP6
SLC16A4	ABHD1	EIF2AK1	ELN	TCN1	HMGA2		NOVA1
HBXIP	PREB	USP42	LIMK1	PLAC1L	LLPH		FOXG1
PROK1	C2orf53	CYTH3	EIF4H	MS4A3	TMBIM4		C14orf23
KCNA10	SLC5A6	MGC12966	LAT2	MS4A2	IRAK3		PRKD1
KCNA2	C2orf28	RAC1	RFC2	MS4A6A	HELB		SCFD1
KCNA3	CAD	KDELR2	CLIP2	MS4A7	GRIP1		COCH
CD53	DNAJC5G	GRID2IP	GTF2IRD1	MS4A14	CAND1		STRN3
C1orf103	TRIM54	ZDHHC4	GTF2I	MS4A5	MDM2		AP4S1
TMEM77	MPV17	ZNF853	WBSCR16	MS4A1	TMBIM4		HECTD1
CEPT1	GTF3C2	PMS2CL	POM121C	MS4A12	IRAK3		HEATR5A
DENND2D	EIF2B4	PMS2CL	HIP1	C11orf64	HELB		C14orf126
CHI3L2	SNX17	PMS2CL	CCL26	MS4A8B	GRIP1		NUBPL
CHIA	ZNF513	PMS2CL	CCL24	MS4A15	CAND1		C14orf128
C1orf88	PPM1G		RHBDD2	MS4A10	MDM2		ARHGAP5
OVGP1	NRBP1		POR	CCDC86			C14orf126
WDR77	FNDC4		TMEM120A	GPR44			NUBPL
ATP5F1	GCKR		STYXL1	PRPF19			C14orf128
C1orf162	C2orf16		MDH2	TMEM109			ARHGAP5
ADORA3	ZNF512		FLJ37078	TMEM132A			
RAP1A	GPN1		HSPB1	SLC15A3			

C1orf183	SUPT7L		YWHAG	CD6
DDX20	SLC4A1AP		SRCRB4D	CD5
KCND3	MRPL33		ZP3	VPS37C
CTTNBP2NL	RBKS		UPK3B	PGA5
WNT2B	BRE		CCDC146	VWCE
ST7L	FOSL2		FGL2	DDB1
CAPZA1	PLB1		PION	DAK
MOV10	PPP1CB		PTPN12	CYBASC3
RHOC	SPDYA		RSBN1L	TMEM138
FAM19A3	TRMT61B		TMEM60	TMEM216
SLC16A1	WDR43		PHTF2	FLJ12529
LRIG2	FAM179A		PHTF2	C11orf79
MAGI3	CLIP4		PHTF2	SYT7
PHTF1	ALK		PHTF2	DAGLA
RSBN1	YPEL5		C11orf9	
PTPN22	LBH		C11orf10	
AP4B1	LCLAT1		FEN1	
DCLRE1B	CAPN13		FADS1	
HIPK1	GALNT14		FADS2	
OLFML3	CAPN14		FADS3	
SYT6	EHD3		BEST1	
TRIM33	XDH		FTH1	
BCAS2	SRD5A2		INCENP	
DENND2C			SCGB1D1	
AMPD1			SCGB2A1	
NRAS			SCGB1D2	
CSDE1			SCGB2A2	
SIKE			ASRGL1	
SYCP1			SCGB1A1	

TSHB			AHNAK
TSPAN2			EEF1G
NGF			TUT1
VANGL1			MTA2
CASQ2			EML3
NHLH2			ROM1
SLC22A15			B3GAT3
C1orf161			GANAB
ATP1A1			C11orf48
C1orf203			UBXN1
CD58			BSCL2
IGSF3			GNG3
CD2			TTC9C
PTGFRN			ZBTB3
IGSF2			POLR2G
TTF2			TAF6L
TRIM45			NXF1
VTCN1			STX5
NOTCH2			SLC3A2
			CHRM1
			SLC22A6
			SLC22A24
			SLC22A25
			SLC22A10
			SLC22A9
			HRASLS5
			LGALS12
			RARRES3
			HRASLS2

			PLA2G16
			HRASLS2
			PLA2G16

Suppl. Table 6. Genes located on chromosomes with significant gains and losses in cluster B primary tumors compared to cluster A primary tumors.

Loss						Gain	Loss	Gain
**2p25-2p11**	**2q11-2q35**	**4q12-4q35**	**5q11-5q35**	**7q35**	**9p24-9p13**	**9q33-9q34**	**10q11-10q23**	**11p11**
FAM110C	MAL	DCUN1D4	PARP8	EPHA1	DOCK8	TNFSF8	LOC84856	TTC17
ACP1	MRPS5	LRRC66	ISL1	TAS2R60	KANK1	TNC	BMS1	HSD17B12
SNTG2	ZNF514	SGCB	PELO	TAS2R41	DMRT1	DEC1	RET	ALKBH3
TPO	ZNF2	SPATA18	ITGA1	ARHGEF5	SMARCA2	C9orf27	CSGALNACT2	ACCS
PXDN	PROM2	USP46	ITGA2	TPK1	FLJ35024	PAPPA	RASGEF1A	EXT2
MYT1L	KCNIP3	RASL11B	MOCS2	CNTNAP2	VLDLR	ASTN2	FXYD4	ALX4
TSSC1	FAHD2A	SCFD2	FST		KIAA0020	TRIM32	HNRNPF	CD82
TTC15	ADRA2B	FIP1L1	NDUFS4		RFX3	TLR4	ZNF487	TSPAN18
ADI1	STARD7	LNX1	ARL15		GLIS3	DBC1	ZNF239	TP53I11
RPS7	TMEM127	CHIC2	HSPB3		SLC1A1	CDK5RAP2	ZNF485	PRDM11
COLEC11	SNRNP200	GSX2	SNX18		C9orf68	MEGF9	ZNF32	SYT13
ALLC	ITPRIPL1	PDGFRA	ESM1		CDC37L1	FBXW2	HNRNPA3P1	CHST1
SOX11	NCAPH	KIT	GZMK		AK3	PSMD5	CXCL12	SLC35C1
CMPK2	NEURL3	KDR	GZMA		RCL1	PHF19	RASSF4	CRY2
RNF144A	KIAA1310	SRD5A3	CDC20B		JAK2	TRAF1	C10orf10	MAPK8IP1
LOC339788	FER1L5	TMEM165	CCNO		INSL6	C5	C10orf25	GYLTL1B
KIDINS220	LMAN2L	CLOCK	DHX29		C9orf46	CEP110	ZNF22	PHF21A
MBOAT2	CNNM4	PDCL2	SKIV2L2		PDCD1LG2	RAB14	LOC100133308	CREB3L1
ASAP2	CNNM3	NMU	SKIV2L2		KIAA1432	GSN	OR13A1	DGKZ
ITGB1BP1	ANKRD23	LOC644145	PPAP2A		ERMP1	STOM	ALOX5	CHRM4
CPSF3	ANKRD39	EXOC1	SLC38A9		MLANA	GGTA1	MARCH8	AMBRA1
ADAM17	SEMA4C	CEP135	DDX4		KIAA2026	DAB2IP	ANUBL1	HARBI1
YWHAQ	FAM178B	KIAA1211	IL31RA		TPD52L3	TTLL11	ZNF488	KIAA0652
TAF1B	COX5B	AASDH	IL6ST		UHRF2	NDUFA8	RBP3	ZNF408

GRHL1	ACTR1B	PPAT	ANKRD55		GLDC	MORN5	GDF2	F2
KLF11	ZAP70	PAICS	MAP3K1		KDM4C	LHX6	GDF10	CKAP5
RRM2	TMEM131	SRP72	C5orf35		PTPRD	RBM18	FRMPD2	LRP4
C2orf48	VWA3B	HOPX	MIER3		TYRP1	MRRF	MAPK8	C11orf49
HPCAL1	CNGA3	SPINK2	GPBP1		MPDZ	PTGS1	ARHGAP22	ARFGAP2
ODC1	INPP4A	REST	PLK2		NFIB	PDCL	WDFY4	PACSIN3
NOL10	UNC50	C4orf14	GAPT		ZDHHC21	RC3H2	C10orf72	DDB2
ATP6V1C2	MGAT4A	POLR2B	RAB3C		CER1	ZBTB6	DRGX	ACP2
PDIA6	C2orf55	IGFBP7	PDE4D		FREM1	ZBTB26	ERCC6	NR1H3
C2orf50	TSGA10	LPHN3	PART1		TTC39B	RABGAP1	PGBD3	MADD
PQLC3	C2orf15	SRD5A2L2	DEPDC1B		SNAPC3	GPR21	CHAT	MYBPC3
ROCK2	LIPT1	EPHA5	ELOVL7		PSIP1	C9orf45	C10orf53	SPI1
E2F6	MITD1	CENPC1	ERCC8		C9orf93	STRBP	OGDHL	PSMC3
GREB1	MRPL30	STAP1	NDUFAF2		BNC2	CRB2	PARG	RAPSN
NTSR2	LYG2	UBA6	ZSWIM6		CNTLN	DENND1A	MSMB	CUGBP1
LPIN1	LYG1	GNRHR	FLJ37543		SH3GL2	LHX2	NCOA4	PTPMT1
TRIB2	TXNDC9	TMPRSS11D	KIF2A		ADAMTSL1	NEK6	TIMM23	KBTBD4
FAM84A	EIF5B	TMPRSS11A	DIMT1L		FAM154A	PSMB7	ASAH2	NDUFS3
NBAS	REV1	TMPRSS11F	IPO11		RRAGA	NR5A1	SGMS1	C1QTNF4
DDX1	AFF3	FLJ41562	LRRC70		HAUS6	NR6A1	A1CF	AGBL2
MYCN	LONRF2	TMPRSS11B	HTR1A		ADFP	OLFML2A	PRKG1	FNBP4
FAM49A	CHST10	YTHDC1	RNF180		DENND4C	RPL35	CSTF2T	NUP160
VSNL1	PDCL3	UGT2A3	RGS7BP		ACER2	ARPC5L		PTPRJ
SMC6	NPAS2	UGT2B11	SFRS12IP1		SLC24A2	GOLGA1		LOC440040
GEN1	RPL31	UGT2B4	SDCCAG10		MLLT3	C9orf126		LOC646813
KCNS3	TBC1D8	UGT2A1	ADAMTS6		KIAA1797	PPP6C		
RDH14	C2orf29	SULT1B1	CENPK		IFNB1	RABEPK		
NT5C1B	RNF149	SULT1E1	PPWD1		IFNW1	HSPA5		
OSR1	CREG2	CSN1S1	TRIM23		KLHL9	GAPVD1		

WDR35	LOC731220	CSN2	C5orf44		IFNA2	MAPKAP1		
MATN3	MAP4K4	STATH	SGTB		IFNA8	PBX3		
LAPTM4A	IL1R2	HTN3	NLN		LOC554202	FAM125B		
SDC1	IL1R1	HTN1	ERBB2IP		IFNE	LMX1B		
PUM2	IL1RL2	CSN1S2B	SFRS12		MTAP	ZBTB43		
RHOB	IL1RL1	ODAM	MAST4		C9orf53	ZBTB34		
HS1BP3	IL18R1	C4orf7	CD180		CDKN2A	RALGPS1		
GDF7	IL18RAP	CSN3	PIK3R1		CDKN2BAS	ANGPTL2		
C2orf43	SLC9A4	C4orf35	SLC30A5		ELAVL2	GARNL3		
APOB	SLC9A2	SMR3A	CCNB1		C9orf82	SLC2A8		
ATAD2B	MFSD9	SMR3B	CENPH		PLAA	ZNF79		
UBXN2A	TMEM182	PROL1	MRPS36		LRRC19	RPL12		
C2orf44	LOC150568	MUC7	CDK7		IFT74	LRSAM1		
FKBP1B	MRPS9	AMBN	CCDC125		TEK	FAM129B		
SF3B14	GPR45	ENAM	TAF9		NCRNA00032	STXBP1		
TP53I3	TGFBRAP1	IGJ	RAD17		MOBKL2B	TTC16		
LOC375190	C2orf49	UTP3	MARVELD2		IFNK	TOR2A		
ITSN2	FHL2	RUFY3	OCLN		C9orf72	SH2D3C		
NCOA1	NCK2	GRSF1	BDP1		LINGO2	CDK9		
CENPO	C2orf40	MOBKL1A	MCCC2		ACO1	FPGS		
ADCY3	UXS1	DCK	CARTPT		DDX58	ENG		
DNAJC27	ST6GAL2	SLC4A4	MAP1B		TOPORS	AK1		
EFR3B	SLC5A7	GC	MRPS27		NDUFB6	ST6GALNAC6		
POMC	SULT1C3	NPFFR2	PTCD2		APTX	ST6GALNAC4		
DNMT3A	SULT1C2	ADAMTS3	ZNF366		DNAJA1	PIP5KL1		
DTNB	SULT1C4	COX18	TNPO1		SMU1	DPM2		
ASXL2	GCC2	ANKRD17	FCHO2		B4GALT1	FAM102A		
KIF3C	LIMS1	ALB	TMEM171		SPINK4	NAIF1		
RAB10	RANBP2	AFP	TMEM174		BAG1	PTGES2		

HADHA	CCDC138	AFM	FOXD1		NFX1	C9orf16			
HADHB	EDAR	RASSF6	BTF3		SUGT1P	DNM1			
GPR113	SH3RF3	IL8	ANKRA2		AQP3	GOLGA2			
SELI	Sep 10	CXCL6	UTP15		NOL6	TRUB2			
C2orf39	MALL	PF4	RGNEF		PRSS3	COQ4			
OTOF	NPHP1	PPBP	ENC1		UBE2R2	SLC27A4			
CIB4	NCRNA00116	CXCL5	HEXB		UBAP2	URM1			
KCNK3	BUB1	CXCL3	GFM2		WDR40A	CERCAM			
C2orf18	ACOXL	PPBPL2	TINP1		UBAP1	ODF2			
CENPA	BCL2L11	CXCL2	FAM169A		KIF24	GLE1			
DPYSL5	ANAPC1	MTHFD2L	GCNT4		NUDT2	SPTAN1			
MAPRE3	MERTK	EREG	HMGCR		C9orf24	WDR34			
TMEM214	TMEM87B	BTC	COL4A3BP		C9orf25	SET			
AGBL5	FBLN7	DKFZP564O0823	POLK		DNAI1	PKN3			
KHK	ZC3H8	RCHY1	C5orf37		CNTFR	ZDHHC12			
CGREF1	ZC3H6	THAP6	SV2C		C9orf23	ZER1			
ABHD1	TTL	C4orf26	IQGAP2		DCTN3	TBC1D13			
PREB	POLR1B	CDKL2	F2RL2		SIGMAR1	ENDOG			
C2orf53	CHCHD5	G3BP2	F2R		GALT	C9orf114			
SLC5A6	SLC20A1	USO1	F2RL1		IL11RA	CCBL1			
C2orf28	CKAP2L	PPEF2	S100Z		CCL27	PHYHD1			
CAD	IL1A	NAAA	CRHBP		CCL19	DOLK			
DNAJC5G	IL1B	SDAD1	AGGF1		DNAJB5	SH3GLB2			
TRIM54	IL1F7	CXCL9	ZBED3		VCP	FAM73B			
MPV17	IL1F9	CXCL10	PDE8B		FANCG	DOLPP1			
GTF3C2	IL1F6	CXCL11	WDR41		PIGO	CRAT			
EIF2B4	IL1F8	ART3	OTP		STOML2	PPP2R4			
SNX17	IL1F5	NUP54	TBCA		KIAA1539	METTL11A			
ZNF513	IL1F10	SCARB2	AP3B1		UNC13B	ASB6			

PPM1G	IL1RN	FAM47E	SCAMP1		LOC158381	PRRX2		
NRBP1	PSD4	STBD1	LHFPL2		RUSC2	PTGES		
FNDC4	PAX8	CCDC158	ARSB		TESK1	TOR1B		
GCKR	LOC654433	SHROOM3	DMGDH		CD72	TOR1A		
C2orf16	RPL23AP7	Sep 11	BHMT2		C9orf100	C9orf78		
ZNF512	SLC35F5	CCNI	BHMT		CA9	USP20		
GPN1	ACTR3	CCNG2	JMY		TPM2	FNBP1		
SUPT7L	DPP10	CXCL13	HOMER1		TLN1	GPR107		
SLC4A1AP	DDX18	CNOT6L	PAPD4		CREB3	FREQ		
MRPL33	CCDC93	MRPL1	CMYA5		GBA2	ASS1		
RBKS	INSIG2	FRAS1	THBS4		NPR2	FUBP3		
BRE	EN1	ANXA3	SERINC5		SPAG8	PRDM12		
FOSL2	MARCO	BMP2K	SPZ1		HINT2	EXOSC2		
PLB1	C1QL2	PAQR3	ZFYVE16		C9orf127	ABL1		
PPP1CB	STEAP3	ARD1B	FAM151B		RECK	FIBCD1		
SPDYA	DBI	GK2	DHFR		GLIPR2	LAMC3		
TRMT61B	TMEM37	GDEP	MSH3		CCIN	AIF1L		
WDR43	SCTR	ANTXR2	RASGRF2		CLTA	NUP214		
FAM179A	PCDP1	PRDM8	CKMT2		GNE	FAM78A		
CLIP4	TMEM177	FGF5	ZCCHC9		RNF38	PPAPDC3		
ALK	PTPN4	C4orf22	ACOT12		MELK	BAT2L		
YPEL5	EPB41L5	BMP3	SSBP2		PAX5	POMT1		
LBH	RALB	PRKG2	ATG10		ZCCHC7	UCK1		
LCLAT1	INHBB	RASGEF1B	RPS23		GRHPR	RAPGEF1		
CAPN13	GLI2	HNRNPD	ATP6AP1L		ZBTB5	MED27		
GALNT14	TFCP2L1	HNRPDL	TMEM167A		POLR1E	NTNG2		
CAPN14	CLASP1	ENOPH1	XRCC4		FBXO10	SETX		
EHD3	MKI67IP	FLJ12993	VCAN		FRMPD1	TTF1		
XDH	TSN	SCD5	HAPLN1		RG9MTD3	C9orf171		

SRD5A2	CNTNAP5	SEC31A	EDIL3		EXOSC3	BARHL1		
MEMO1	GYPC	THAP9	COX7C		WDR32	DDX31		
DPY30	BIN1	LIN54	RASA1		MCART1	GTF3C4		
SPAST	ERCC3	COPS4	CCNH		SHB	C9orf98		
SLC30A6	MAP3K2	PLAC8	TMEM161B		ALDH1B1	C9orf9		
NLRC4	PROC	COQ2	LOC645323		CNTNAP3	TSC1		
YIPF4	IWS1	HPSE	MEF2C			GFI1B		
BIRC6	MYO7B	HELQ	CETN3			GTF3C5		
TTC27	LIMS2	MRPS18C	POLR3G			CEL		
LOC285045	GPR17	FAM175A	GPR98			RALGDS		
LTBP1	SFT2D3	AGPAT9	ARRDC3			GBGT1		
RASGRP3	WDR33	NKX6-1	LOC100129716			ABO		
CRIM1	AMMECR1L	CDS1	FLJ42709			SURF6		
FEZ2	SAP130	WDFY3	NR2F1			SURF4		
VIT	UGCGL1	ARHGAP24	FAM172A			REXO4		
STRN	HS6ST1	MAPK10	POU5F2			ADAMTSL2		
HEATR5B	LOC440905	PTPN13	C5orf36			DBH		
CCDC75	CCDC115	SLC10A6	ANKRD32			SARDH		
EIF2AK2	IMP4	C4orf36	MCTP1			VAV2		
CEBPZ	PTPN18	AFF1	FAM81B			BRD3		
C2orf56	FAM123C	KLHL8	TTC37			RXRA		
PRKD3	ARHGEF4	HSD17B13	ARSK			COL5A1		
QPCT	PLEKHB2	HSD17B11	RFESD			FCN2		
CDC42EP3	C2orf27A	NUDT9	RHOBTB3			FCN1		
FAM82A1	GPR39	SPARCL1	GLRX			OLFM1		
CYP1B1	LYPD1	DSPP	C5orf27			KIAA0649		
C2orf58	NAP5	DMP1	ELL2			MRPS2		
ATL2	MGAT5	IBSP	PCSK1			LCN1		
HNRPLL	TMEM163	MEPE	CAST			PAEP		

GALM	ACMSD	SPP1	ERAP1			GLT6D1		
SFRS7	CCNT2	PKD2	ERAP2			KCNT1		
GEMIN6	YSK4	ABCG2	LNPEP			CAMSAP1		
DHX57	RAB3GAP1	PPM1K	LIX1			UBAC1		
MORN2	ZRANB3	HERC6	RIOK2			NACC2		
LOC100271715	R3HDM1	HERC5	RGMB			LHX3		
SOS1	UBXN4	HERC3	CHD1			QSOX2		
CDKL4	LCT	NAP1L5	FAM174A			LOC26102		
MAP4K3	MCM6	FAM13AOS	ST8SIA4			GPSM1		
TMEM178	DARS	FAM13A	SLCO4C1			CARD9		
THUMPD2	CXCR4	TIGD2	SLCO6A1			SDCCAG3		
SLC8A1	THSD7B	GPRIN3	PAM			PMPCA		
EML4	HNMT	SNCA	GIN1			INPP5E		
COX7A2L	SPOPL	MMRN1	HISPPD1			SEC16A		
KCNG3	NXPH2	KIAA1680	C5orf30			C9orf163		
MTA3	LRP1B	TMSL3	NUDT12			NOTCH1		
HAAO	KYNU	GRID2	EFNA5			EGFL7		
LOC100129726	ARHGAP15	ATOH1	FBXL17			AGPAT2		
THADA	GTDC1	SMARCAD1	FER			FAM69B		
PLEKHH2	ZEB2	PGDS	PJA2			LCN6		
DYNC2LI1	PABPCP2	PDLIM5	MAN2A1			LCN8		
ABCG5	ACVR2A	BMPR1B	FLJ43080			TMEM141		
ABCG8	ORC4L	UNC5C	SLC25A46			KIAA1984		
LRPPRC	MBD5	PDHA2	TSLP			C9orf86		
PPM1B	EPC2	C4orf37	WDR36			EDF1		
SLC3A1	KIF5C	RAP1GDS1	CAMK4			TRAF2		
PREPL	LYPD6B	TSPAN5	STARD4			PTGDS		
C2orf34	LYPD6	EIF4E	C5orf13			ABCA2		
SIX3	MMADHC	METAP1	C5orf26			MAN1B1		

SIX2	RND3	ADH4	EPB41L4A			GRIN1		
SRBD1	TNFAIP6	ADH6	APC			SSNA1		
PRKCE	RIF1	ADH1B	SRP19			C9orf75		
EPAS1	NEB	ADH1C	REEP5			NDOR1		
ATP6V1E2	ARL5A	ADH7	DCP2			COBRA1		
RHOQ	CACNB4	C4orf17	MCC			NRARP		
PIGF	STAM2	RG9MTD2	TSSK1B			EXD3		
CRIPT	FMNL2	MTTP	YTHDC2			PNPLA7		
SOCS5	PRPF40A	DAPP1	KCNN2			ZMYND19		
MCFD2	ARL6IP6	DNAJB14	TRIM36			ARRDC1		
TTC7A	RPRM	H2AFZ	PGGT1B			C9orf37		
C2orf61	GALNT13	DDIT4L	CCDC112			EHMT1		
CALM2	KCNJ3	EMCN	FEM1C			FLJ40292		
EPCAM	NR4A2	PPP3CA	TICAM2			CACNA1B		
MSH2	GPD2	BANK1	TMED7					
KCNK12	GALNT5	SLC39A8	CDO1					
MSH6	ERMN	NFKB1	ATG12					
FBXO11	CYTIP	MANBA	AP3S1					
FOXN2	ACVR1C	UBE2D3	LVRN					
KLRAQ1	ACVR1	CISD2	COMMD10					
STON1	UPP2	NHEDC1	SEMA6A					
STON1-GTF2A1L	CCDC148	NHEDC2	DTWD2					
GTF2A1L	PKP4	BDH2	DMXL1					
LHCGR	DAPL1	CENPE	TNFAIP8					
FSHR	TANC1	TACR3	HSD17B4					
NRXN1	WDSUB1	CXXC4	FAM170A					
ASB3	BAZ2B	TET2	PRR16					
C2orf30	MARCH7	PPA2	FTMT					
GPR75	CD302	FLJ20184	SRFBP1					

PSME4	LY75	INTS12	LOX					
ACYP2	PLA2R1	GSTCD	SNCAIP					
C2orf73	ITGB6	NPNT	SNX2					
SPTBN1	RBMS1	TBCKL	SNX24					
EML6	TANK	SCYE1	PPIC					
RTN4	PSMD14	DKK2	PRDM6					
C2orf63	TBR1	PAPSS1	CEP120					
RPS27A	SLC4A10	SGMS2	CSNK1G3					
MTIF2	DPP4	CYP2U1	ZNF608					
CCDC88A	GCG	HADH	GRAMD3					
CCDC104	FAP	LEF1	ALDH7A1					
SMEK2	IFIH1	LOC285456	PHAX					
PNPT1	GCA	RPL34	LMNB1					
EFEMP1	KCNH7	OSTC	MARCH3					
CCDC85A	FIGN	AGXT2L1	MEGF10					
VRK2	GRB14	COL25A1	PRRC1					
FANCL	COBLL1	SEC24B	FLJ33630					
BCL11A	SLC38A11	CCDC109B	SLC12A2					
PAPOLG	SCN3A	CASP6	FBN2					
REL	SCN2A	PLA2G12A	SLC27A6					
PUS10	CSRNP3	CFI	ISOC1					
PEX13	GALNT3	GAR1	ADAMTS19					
KIAA1841	TTC21B	RRH	CHSY3					
AHSA2	SCN1A	EGF	LYRM7					
USP34	SCN9A	ELOVL6	CDC42SE2					
XPO1	SCN7A	ENPEP	RAPGEF6					
CCT4	XIRP2	PITX2	FNIP1					
COMMD1	B3GALT1	C4orf32	ACSL6					
B3GNT2	STK39	C4orf16	IL3					

TMEM17	LASS6	TIFA	CSF2					
EHBP1	NOSTRIN	ALPK1	P4HA2					
OTX1	SPC25	NEUROG2	PDLIM4					
C2orf86	G6PC2	LOC91431	SLC22A4					
MDH1	ABCB11	C4orf21	SLC22A5					
UGP2	DHRS9	LARP7	LOC441108					
VPS54	LRP2	ANK2	IRF1					
PELI1	BBS5	CAMK2D	IL5					
HSPC159	KBTBD10	ARSJ	RAD50					
AFTPH	FASTKD1	UGT8	IL13					
SERTAD2	PPIG	NDST4	IL4					
SLC1A4	C2orf77	TRAM1L1	KIF3A					
CEP68	PHOSPHO2	NDST3	Sep 08					
RAB1A	KLHL23	PRSS12	ANKRD43					
ACTR2	SSB	CEP170L	SHROOM1					
SPRED2	METTL5	METTL14	GDF9					
MEIS1	UBR3	SEC24D	UQCRQ					
ETAA1	MYO3B	SYNPO2	LEAP2					
C1D	GAD1	MYOZ2	AFF4					
WDR92	GORASP2	USP53	ZCCHC10					
PNO1	TLK1	FABP2	HSPA4					
PPP3R1	METTL8	PDE5A	FSTL4					
CNRIP1	C2orf37	MAD2L1	C5orf15					
PLEK	CYBRD1	PRDM5	VDAC1					
APLF	DYNC1I2	C4orf31	CDKN2AIPNL					
PROKR1	SLC25A12	TNIP3	TCF7					
ARHGAP25	HAT1	QRFPR	SKP1					
BMP10	MAP1D	ANXA5	PPP2CA					
GKN2	DLX1	TMEM155	CDKL3					

GKN1	DLX2	EXOSC9	UBE2B					
ANTXR1	ITGA6	CCNA2	PHF15					
GFPT1	PDK1	BBS7	SAR1B					
NFU1	RAPGEF4	TRPC3	SEC24A					
AAK1	ZAK	KIAA1109	CAMLG					
ANXA4	CDCA7	ADAD1	DDX46					
GMCL1	SP3	IL2	C5orf24					
SNRNP27	OLA1	IL21	TXNDC15					
MXD1	CIR	BBS12	PCBD2					
ASPRV1	SCRN3	FGF2	CATSPER3					
C2orf42	GPR155	NUDT6	H2AFY					
TIA1	WIPF1	SPATA5	C5orf20					
PCYOX1	CHRNA1	LOC285419	NEUROG1					
SNRPG	CHN1	ANKRD50	CXCL14					
TGFA	ATF2	FAT4	LOC153328					
ADD2	ATP5G3	INTU	IL9					
CLEC4F	KIAA1715	SLC25A31	FBXL21					
CD207	HOXD13	HSPA4L	LECT2					
VAX2	HOXD12	PLK4	TGFBI					
ATP6V1B1	HOXD11	MFSD8	SMAD5OS					
ANKRD53	HOXD10	C4orf29	SMAD5					
TEX261	HOXD8	LARP2	TRPC7					
NAGK	HOXD4	PGRMC2	SPOCK1					
MCEE	HOXD3	PHF17	KLHL3					
MPHOSPH10	HOXD1	SCLT1	HNRNPA0					
PAIP2B	MTX2	C4orf33	MYOT					
ZNF638	HNRNPA3	PCDH10	PKD2L2					
DYSF	NFE2L2	PCDH18	FAM13B					
CYP26B1	LOC100130691	SLC7A11	WNT8A					

EXOC6B	AGPS	CCRN4L	NME5					
SPR	TTC30B	ELF2	BRD8					
EMX1	TTC30A	C4orf49	KIF20A					
SFXN5	PDE11A	NDUFC1	CDC23					
RAB11FIP5	RBM45	NARG1	GFRA3					
C2orf7	OSBPL6	RAB33B	CDC25C					
CCT7	PRKRA	SETD7	KDM3B					
EGR4	FKBP7	MGST2	REEP2					
ALMS1	PLEKHA3	MAML3	EGR1					
ALMS1P	TTN	SCOC	ETF1					
TPRKB	CCDC141	CLGN	HSPA9					
DUSP11	SESTD1	ELMOD2	CTNNA1					
C2orf78	ZNF385B	UCP1	LRRTM2					
STAMBP	CWC22	TBC1D9	SIL1					
ACTG2	UBE2E3	RNF150	MATR3					
DGUOK	ITGA4	ZNF330	PAIP2					
TET3	CERKL	IL15	SLC23A1					
MOBKL1B	NEUROD1	INPP4B	MGC29506					
MTHFD2	SSFA2	USP38	DNAJC18					
SLC4A5	PPP1R1C	GAB1	TMEM173					
DCTN1	PDE1A	SMARCA5	UBE2D2					
WDR54	DNAJC10	GYPA	CXXC5					
RTKN	FRZB	HHIP	PSD2					
INO80B	NCKAP1	ANAPC10	NRG2					
WBP1	DUSP19	ABCE1	PURA					
MOGS	NUP35	OTUD4	C5orf32					
MRPL53	ZNF804A	SMAD1	PFDN1					
CCDC142	ZC3H15	MMAA	HBEGF					
TTC31	ITGAV	LOC646603	SLC4A9					

LOC151534	FAM171B	ZNF827	ANKHD1					
PCGF1	ZSWIM2	SLC10A7	APBB3					
DQX1	CALCRL	POU4F2	SLC35A4					
HTRA2	TFPI	TTC29	CD14					
LOXL3	GULP1	EDNRA	TMCO6					
DOK1	DIRC1	TMEM184C	NDUFA2					
C2orf65	COL3A1	LOC90826	IK					
SEMA4F	COL5A2	ARHGAP10	WDR55					
HK2	WDR75	NR3C2	HARS					
POLE4	SLC40A1	DCLK2	HARS2					
TACR1	ASNSD1	LRBA	ZMAT2					
FAM176A	ANKAR	MAB21L2	PCDHA1					
MRPL19	OSGEPL1	RPS3A	PCDHA2					
C2orf3	ORMDL1	SH3D19	PCDHA3					
LRRTM4	PMS1	ESSPL	PCDHA4					
REG1B	MSTN	FAM160A1	PCDHA5					
REG1P	C2orf88	PET112L	PCDHB1					
REG3A	HIBCH	FBXW7	PCDHB2					
CTNNA2	INPP1	TMEM154	PCDHB3					
LRRTM1	MFSD6	TIGD4	PCDHB4					
SUCLG1	TMEM194B	ARFIP1	PCDHB5					
DNAH6	NAB1	FHDC1	PCDHB6					
LOC129293	GLS	TRIM2	PCDHB7					
KCMF1	STAT1	MND1	PCDHB16					
TCF7L1	STAT4	KIAA0922	PCDHB9					
TGOLN2	MYO1B	TLR2	PCDHB10					
RETSAT	OBFC2A	RNF175	PCDHB11					
ELMOD3	SDPR	DCHS2	PCDHB12					
CAPG	TMEFF2	PLRG1	PCDHB14					

MAT2A	SLC39A10	FGB	PCDHB15					
GGCX	DNAH7	FGA	SLC25A2					
VAMP5	STK17B	FGG	TAF7					
RNF181	HECW2	LRAT	PCDHGA1					
TMEM150	CCDC150	RBM46	PCDHGA2					
USP39	GTF3C3	NPY2R	PCDHGA3					
GNLY	PGAP1	MAP9						
ATOH8	ANKRD44	GUCY1A3	DIAPH1					
ST3GAL5	SF3B1	GUCY1B3	HDAC3					
POLR1A	COQ10B	ACCN5	RELL2					
PTCD3	HSPD1	TDO2	FCHSD1					
IMMT	HSPE1	CTSO	ARAP3					
MRPL35	MOBKL3	PDGFC	PCDH1					
REEP1	RFTN2	GLRB	KIAA0141					
KDM3A	MARS2	GRIA2	PCDH12					
VPS24	BOLL	C4orf18	RNF14					
RNF103	PLCL1	TMEM144	GNPDA1					
RMND5A	SATB2	RXFP1	NDFIP1					
CD8A	C2orf69	ETFDH	SPRY4					
CD8B	C2orf60	PPID	FGF1					
LOC285074	C2orf47	FNIP2	ARHGAP26					
NCRNA00152	LOC26010	C4orf45	NR3C1					
KRCC1	KCTD18	RAPGEF2	HMHB1					
SMYD1	SGOL2	FSTL5	YIPF5					
FABP1	AOX1	NAF1	KCTD16					
THNSL2	CLK1	NPY1R	PRELID2					
C2orf51	PPIL3	NPY5R	GRXCR2					
EIF2AK3	NIF3L1	TKTL2	SH3RF2					
RPIA	ORC2L	MARCH1	LARS					

	FAM126B	TRIM61	RBM27					
	NDUFB3	TRIM60	TCERG1					
	CFLAR	TMEM192	GPR151					
	CASP10	KLHL2	PPP2R2B					
	CASP8	SC4MOL	STK32A					
	ALS2CR12	CPE	DPYSL3					
	TRAK2	TLL1	JAKMIP2					
	ALS2CR11	SPOCK3	SPINK1					
	ALS2CR4	ANXA10	SCGB3A2					
	MPP4	DDX60	SPINK5					
	ALS2	DDX60L	SPINK5L2					
	PFTK2	PALLD	SPINK7					
	FZD7	CBR4	FBXO38					
	SUMO1	SH3RF1	HTR4					
	NOP58	NEK1	ADRB2					
	BMPR2	CLCN3	SH3TC2					
	FAM117B	C4orf27	ABLIM3					
	ICA1L	MFAP3L	AFAP1L1					
	WDR12	AADAT	PCYOX1L					
	ALS2CR8	GALNTL6	IL17B					
	NBEAL1	GALNT7	LOC728264					
	CYP20A1	HMGB2	CSNK1A1					
	ABI2	SAP30	FLJ41603					
	RAPH1	SCRG1	PPARGC1B					
	CD28	HAND2	PDE6A					
	CTLA4	NBLA00301	SLC26A2					
	ICOS	FBXO8	TIGD6					
	PARD3B	KIAA1712	CSF1R					
	NRP2	HPGD	PDGFRB					

	INO80D	GLRA3	CDX1					
	NDUFS1	ADAM29	SLC6A7					
	GPR1	GPM6A	CAMK2A					
	ZDBF2	WDR17	TCOF1					
	ADAM23	SPATA4	CD74					
	DYTN	ASB5	RPS14					
	FASTKD2	VEGFC	NDST1					
	CPO	NEIL3	SYNPO					
	KLF7	AGA	MYOZ3					
	CREB1	LOC285501	RBM22					
	FAM119A	MGC45800	DCTN4					
	CCNYL1	ODZ3	MST150					
	FZD5	DCTD	ZNF300					
	PLEKHM3	C4orf38	LOC134466					
	CRYGD	WWC2	GPX3					
	CRYGC	CDKN2AIP	TNIP1					
	CRYGB	ING2	ANXA6					
	CRYGA	RWDD4A	CCDC69					
	IDH1	C4orf41	GM2A					
	PIP5K3	STOX2	SLC36A3					
	PTH2R	ENPP6	SLC36A2					
	MAP2	IRF2	SLC36A1					
	C2orf21	CASP3	FAT2					
	RPE	CCDC111	SPARC					
	C2orf67	MLF1IP	ATOX1					
	ACADL	ACSL1	G3BP1					
	MYL1	SLC25A4	GLRA1					
	LANCL1	KIAA1430	NMUR2					
	CPS1	SNX25	GRIA1					

	LOC29034	LRP2BP	FAM114A2					
	ERBB4	ANKRD37	MFAP3					
	IKZF2	UFSP2	GALNT10					
	SPAG16	C4orf47	SAP30L					
	VWC2L	CCDC110	HAND1					
	BARD1	PDLIM3	LARP1					
	ABCA12	SORBS2	C5orf4					
	ATIC	TLR3	CNOT8					
	FN1	FAM149A	GEMIN5					
	MREG	CYP4V2	MRPL22					
	PECR	KLKB1	SGCD					
	TMEM169	F11	TIMD4					
	XRCC5	FAT1	HAVCR1					
	MARCH4	ZFP42	HAVCR2					
	SMARCAL1	TRIML2	MED7					
	RPL37A	TRIML1	ITK					
	IGFBP2		CYFIP2					
	IGFBP5		ADAM19					
	TNP1		SOX30					
	DIRC3		C5orf52					
	TNS1		LSM11					
	RUFY4		CLINT1					
	IL8RA		EBF1					
	ARPC2		RNF145					
	AAMP		UBLCP1					
	TMBIM1		IL12B					
	PNKD		ADRA1B					
	SLC11A1		TTC1					
	CTDSP1		PWWP2A					

	VIL1		FABP6					
	USP37		CCNJL					
	RQCD1		C1QTNF2					
	PLCD4		C5orf54					
	ZNF142		SLU7					
	BCS1L		PTTG1					
	RNF25		ATP10B					
	TTLL4		GABRB2					
	CYP27A1		GABRA6					
	PRKAG3		GABRA1					
	WNT6		GABRG2					
	WNT10A		CCNG1					
	FEV		NUDCD2					
	CCDC108		HMMR					
	NHEJ1		MAT2B					
	SLC23A3		ODZ2					
	C2orf24		WWC1					
	FAM134A		RARS					
	ZFAND2B		PANK3					
	ABCB6		SLIT3					
	ANKZF1		CCDC99					
	GLB1L		DOCK2					
	STK16		LOC100131897					
	TUBA4A		FOXI1					
	TUBA4B		LCP2					
	DNAJB2		KCNMB1					
	PTPRN		KCNIP1					
	DNPEP		GABRP					
	DES		RANBP17					

	SPEG		TLX3						
	GMPPA		NPM1						
	ACCN4		FGF18						
	CHPF		FBXW11						
	INHA		STK10						
	STK11IP		UBTD2						
	SLC4A3		SH3PXD2B						
			NEURL1B						
			DUSP1						
			ERGIC1						
			RPL26L1						
			ATP6V0E1						
			C5orf41						
			BNIP1						
			NKX2-5						
			STC2						
			BOD1						
			CPEB4						
			HMP19						
			MSX2						
			DRD1						
			SFXN1						
			HRH2						
			CPLX2						
			C5orf25						
			KIAA1191						
			ARL10						
			HIGD2A						
			CLTB						

			FAF2					
			RNF44					
			PCDH24					
			GPRIN1					
			SNCB					
			UNC5A					
			HK3					
			UIMC1					
			ZNF346					
			FGFR4					
			NSD1					
			RAB24					
			MXD3					
			LMAN2					
			RGS14					
			SLC34A1					
			F12					
			GRK6					
			PRR7					
			DBN1					
			FLJ10404					
			LOC202181					
			PROP1					
			RMND5B					
			GMCL1L					
			HNRNPAB					
			AGXT2L2					
			COL23A1					
			CLK4					

			ZNF354A					
			ZNF354B					
			ZFP2					
			ZNF454					
			GRM6					
			DKFZp686E2433					
			ZNF354C					
			ADAMTS2					
			RUFY1					
			HNRNPH1					
			CANX					
			MAML1					
			LTC4S					
			MGAT4B					
			SQSTM1					
			C5orf45					
			TBC1D9B					
			RNF130					
			RASGEF1C					
			MAPK9					
			GFPT2					
			CNOT6					
			FLT4					
			MGAT1					
			BTNL3					
			BTNL9					
			TRIM7					
			TRIM41					
			GNB2L1					

			TRIM52					

Gain							Loss
**11q12-11q13**	**12q13**	**14q32**	**15q24**	**17q11-17q25**	**19p13-19p12**	**20q11-20q12**	**21q11-21q21**
CNTF	SFRS2IP	FOXN3	HEXA	WSB1	MIER2	DEFB118	LIPI
APLNR	SLC38A1	PRO1768	C15orf34	KSR1	THEG	DEFB119	RBM11
TNKS1BP1	SLC38A2	C14orf143	TMEM202	NOS2	SHC2	DEFB123	ABCC13
SSRP1	SLC38A4	TDP1	ARIH1	LOC201229	ODF3L2	REM1	HSPA13
P2RX3	AMIGO2	KCNK13	BBS4	NLK	CDC34	HM13	SAMSN1
PRG3	FAM113B	PSMC1	ADPGK	FLJ40504	HCN2	ID1	NRIP1
PRG2	RPAP3	C14orf102	NEO1	TMEM97	RNF126	COX4I2	USP25
SLC43A3	P11	CALM1	HCN4	IFT20	PRSSL1	BCL2L1	C21orf34
RTN4RL2	RAPGEF3	TTC7B	C15orf60	TNFAIP1	PALM	TPX2	CXADR
SLC43A1	SLC48A1	RPS6KA5	NPTN	POLDIP2	AZU1	MYLK2	BTG3
TIMM10	HDAC7	C14orf159	CD276	TMEM199	CFD	DUSP15	C21orf91
UBE2L6	VDR	GPR68	LOXL1	SEBOX	ARID3A	TTLL9	NCRNA00157
SERPING1	TMEM106C	CCDC88C	STOML1	VTN	C19orf6	PDRG1	CHODL
YPEL4	SENP1	SMEK1	PML	SARM1	ABCA7	C20orf160	PRSS7
CLP1	PFKM	CATSPERB	LOC283731	SLC46A1	HMHA1	HCK	C21orf131
ZDHHC5	ASB8	TC2N	ISLR	SLC13A2	POLR2E	TM9SF4	NCAM2
MED19	H1FNT	FBLN5	STRA6	FOXN1	GPX4	PLAGL2	NCRNA00158
TMX2	ZNF641	TRIP11	CCDC33	UNC119	SBNO2	POFUT1	MRPL39
C11orf31	C12orf54	ATXN3	CYP11A1	PIGS	STK11	KIF3B	JAM2
CTNND1	LALBA	NDUFB1	SEMA7A	SPAG5	C19orf26	ASXL1	ATP5J
OR9Q1	C12orf41	CPSF2	UBL7	FLJ25006	ATP5D	C20orf112	GABPA
OR10W1	CCNT1	SLC24A4	ARID3B	KIAA0100	MIDN	LOC284805	APP
LPXN	ADCY6	RIN3	CLK3	SDF2	CIRBP	COMMD7	CYYR1
ZFP91	CACNB3	LGMN	EDC3	SUPT6H	EFNA2	DNMT3B	ADAMTS1

ZFP91-CNTF	DDX23	GOLGA5	CYP1A1	PROCA1	MUM1	MAPRE1	ADAMTS5
GLYAT	RND1	CHGA	CSK	RAB34	GAMT	EFCAB8	C21orf94
GLYATL1	CCDC65	ITPK1	LMAN1L	RPL23A	DAZAP1	SPAG4L	NCRNA00161
FAM111B	FKBP11	MOAP1	ULK3	TLCD1	RPS15	BPIL3	N6AMT1
FAM111A	ARF3	C14orf142	MPI	NEK8	APC2	C20orf185	RNF160
DTX4	WNT10B	UBR7	C15orf17	TRAF4	C19orf25	C20orf186	RWDD2B
OSBP	WNT1	BTBD7	COX5A	C17orf63	REEP6	BASE	USP16
PATL1	PRKAG1	COX8C	RPP25	ERAL1	MBD3	C20orf71	CCT8
STX3	MLL2	KIAA1409	SCAMP5	FLOT2	UQCR	PLUNC	C21orf7
MRPL16	RHEBL1	PRIMA1	PPCDC	DHRS13	TCF3	C20orf114	C21orf109
GIF	DHH	FAM181A	C15orf39	PHF12	ONECUT3	CDK5RAP1	BACH1
TCN1	LMBR1L	ASB2	COMMD4	SEZ6	ATP8B3	SNTA1	C21orf41
PLAC1L	TUBA1B	C14orf48	NEIL1	PIPOX	REXO1	CBFA2T2	GRIK1
MS4A3	TUBA1A	OTUB2	MAN2C1	MYO18A	KLF16	E2F1	NCRNA00110
MS4A2	TUBA1C	DDX24	SIN3A	CRYBA1	FAM108A1	PXMP4	CLDN17
MS4A6A	PRPH	IFI27L1	PTPN9	NUFIP2	ADAT3	ZNF341	
MS4A7	TROAP	IFI27	SNUPN	TAOK1	SCAMP4	CHMP4B	
MS4A14	DNAJC22	IFI27L2	IMP3	ABHD15	CSNK1G2	RALY	
MS4A5	SPATS2	PPP4R4	SNX33	TP53I13	BTBD2	EIF2S2	
MS4A1	MCRS1	SERPINA10	CSPG4	GIT1	MKNK2	ASIP	
MS4A12	FAM186B	SERPINA6	ODF3L1	ANKRD13B	MOBKL2A	AHCY	
C11orf64	PRPF40B	SERPINA1	UBE2Q2	CORO6	AP3D1	ITCH	
MS4A8B	FMNL3	SERPINA9	FBXO22	SSH2	DOT1L	DYNLRB1	
MS4A15	TMBIM6	SERPINA12	NRG4	EFCAB5	SF3A2	MAP1LC3A	
MS4A10	KIAA1602	SERPINA5	C15orf27	CCDC55	OAZ1	PIGU	
CCDC86	BCDIN3D	SERPINA3	ETFA	SLC6A4	LSM7	TP53INP2	
GPR44	FAIM2	SERPINA13	ISL2	BLMH	SPPL2B	NCOA6	
PRPF19	AQP2	GSC	SCAPER	CPD	TMPRSS9	GGT7	
TMEM109	AQP5	DICER1	RCN2	GOSR1	TIMM13	ACSS2	

TMEM132A	AQP6	CLMN	PSTPIP1	CRLF3	LMNB2	GSS	
SLC15A3	RACGAP1	C14orf139	TSPAN3	ATAD5	GADD45B	MYH7B	
CD6	ACCN2	C14orf49	SGK269	C17orf42	GNG7	TRPC4AP	
CD5	SMARCD1	GLRX5	C15orf5	ADAP2	DIRAS1	EDEM2	
VPS37C	GPD1	TCL6	HMG20A	RNF135	SLC39A3	PROCR	
PGA5	C12orf62	TCL1B	LINGO1	NF1	SGTA	MMP24	
VWCE	LASS5	TCL1A	TBC1D2B	OMG	THOP1	EIF6	
DDB1	LIMA1	C14orf132		EVI2B	ZNF554	FAM83C	
DAK	LARP4	BDKRB2		EVI2A	ZNF555	UQCC	
CYBASC3	DIP2B	BDKRB1		RAB11FIP4	ZNF556	GDF5	
TMEM138	ATF1	ATG2B		C17orf79	ZNF57	CEP250	
TMEM216	TMPRSS12	C14orf129		UTP6	ZNF77	C20orf173	
FLJ12529	METTL7A	AK7		SUZ12	TLE6	ERGIC3	
C11orf79	SLC11A2	PAPOLA		RHOT1	TLE2	FER1L4	
SYT7	LETMD1	VRK1		RHBDL3	AES	SPAG4	
DAGLA	CSRNP2	C14orf177		C17orf75	GNA11	RBM12	
C11orf9	TFCP2	BCL11B		ZNF207	GNA15	NFS1	
C11orf10	POU6F1	SETD3		PSMD11	NCLN	ROMO1	
FEN1	DAZAP2	CCNK		CDK5R1	BRUNOL5	RBM39	
FADS1	SMAGP	CCDC85C		MYO1D	NFIC	PHF20	
FADS2	BIN2	CYP46A1		TMEM98	FZR1	C20orf152	
FADS3	ELA1	EML1		SPACA3	C19orf28	EPB41L1	
BEST1	GALNT6	EVL		ACCN1	HMG20B	C20orf4	
FTH1	SLC4A8	YY1		CCL7	GIPC3	DLGAP4	
INCENP	SCN8A	SLC25A29		CCL11	TBXA2R	MYL9	
SCGB1D1	ANKRD33	C14orf68		CCL8	PIP5K1C	TGIF2	
SCGB2A1	ACVRL1	WARS		CCL13	TJP3	C20orf24	
SCGB1D2	ACVR1B	WDR25		TMEM132E	APBA3	SLA2	
SCGB2A2	GRASP	BEGAIN		CCT6B	MRPL54	NDRG3	

ASRGL1	C12orf44	DLK1		ZNF830	MATK	DSN1	
SCGB1A1	LOC283404	MEG3		LIG3	ZFR2	C20orf117	
AHNAK	KRT7	MEG8		RFFL	ATCAY	SAMHD1	
EEF1G	KRT81	DIO3		RAD51L3	ITGB1BP3	RBL1	
TUT1	KRT86	PPP2R5C		FNDC8	DAPK3	C20orf132	
MTA2	KRT83	DYNC1H1		NLE1	EEF2	RPN2	
EML3	KRT85	HSP90AA1		UNC45B	PIAS4	GHRH	
ROM1	KRT84	WDR20		AMAC1	ZBTB7A	MANBAL	
B3GAT3	KRT82	RAGE		SLFN5	MAP2K2	SRC	
GANAB	KRT75	ZNF839		SLFN11	CREB3L3	NNAT	
C11orf48	KRT6B	CINP		SLFN12	SIRT6	BLCAP	
UBXN1	KRT6A	TECPR2		SLFN13	ANKRD24	CTNNBL1	
BSCL2	KRT5	ANKRD9		SLFN12L	CCDC94	VSTM2L	
GNG3	KRT71	RCOR1		SLFN14	FSD1	KIAA0406	
TTC9C	KRT74	TRAF3		PEX12	STAP2	RPRD1B	
ZBTB3	KRT72	AMN		AP2B1	MPND	TGM2	
POLR2G	KRT73	CDC42BPB		GAS2L2	SH3GL1	BPI	
TAF6L	KRT2	TNFAIP2		C17orf50	CHAF1A	LBP	
NXF1	KRT1	EIF5		MMP28	UBXN6	SNHG11	
STX5	KRT76	MARK3		TAF15	HDGF2	KIAA1219	
SLC3A2	KRT3			C17orf66	LRG1	ADIG	
CHRM1	KRT4			CCL5	SEMA6B	SLC32A1	
SLC22A6	KRT79			RDM1	TNFAIP8L1	ACTR5	
SLC22A24	KRT78			LYZL6	C19orf10	PPP1R16B	
SLC22A25	KRT8			CCL15	DPP9	FAM83D	
SLC22A10	KRT18			CCL16	TICAM1	DHX35	
SLC22A9	EIF4B			CCL14	M6PRBP1	LOC339568	
HRASLS5	TENC1			CCL23	ARRDC5	MAFB	
LGALS12	SPRYD3			CCL18	UHRF1	TOP1	

RARRES3	IGFBP6			CCL3	KDM4B	PRO0628	
HRASLS2	CSAD			ZNHIT3	PTPRS	PLCG1	
PLA2G16	ITGB7			MYO19	ZNRF4	ZHX3	
ATL3	MFSD5			PIGW	SAFB2	CHD6	
RTN3	ESPL1			GGNBP2	SAFB	PTPRT	
C11orf84	PFDN5			DHRS11	HSD11B1L		
MARK2	C12orf10			MRM1	LONP1		
NAT11	AAAS			LHX1	TMEM146		
COX8A	SP7			AATF	NRTN		
OTUB1	SP1			ACACA	FUT6		
MACROD1	AMHR2			C17orf78	FUT3		
FLRT1	PRR13			TADA2L	FUT5		
STIP1	PCBP2			DUSP14	NDUFA11		
FERMT3	MAP3K12			AP1GBP1	CAPS		
TRPT1	TARBP2			DDX52	RANBP3		
NUDT22	NPFF			HNF1B	RFX2		
DNAJC4	ATF7			MRPL45	ACSBG2		
VEGFB	ATP5G2			SOCS7	MLLT1		
FKBP2	CALCOCO1			ARHGAP23	ACER1		
PLCB3	HOXC13			SNIP	CLPP		
BAD	HOXC12			MLLT6	GTF2F1		
GPR137	HOXC11			CISD3	KHSRP		
C11orf20	HOXC10			PCGF2	SLC25A41		
ESRRA	HOXC9			PSMB3	SLC25A23		
PRDX5	HOXC8			PIP4K2B	CRB3		
RPS6KA4	HOXC6			CCDC49	DENND1C		
SLC22A11	HOXC5			RPL23	TNFSF9		
SLC22A12	HOXC4			LASP1	CD70		
NRXN2	SMUG1			FBXO47	TNFSF14		

RASGRP2	CBX5			PLXDC1	C3		
PYGM	HNRNPA1			CACNB1	TRIP10		
SF1	NFE2			RPL19	SH2D3A		
MAP4K2	COPZ1			LOC90110	VAV1		
MEN1	GPR84			FBXL20	EMR1		
EHD1	ITGA5			MED1	EMR4P		
ATG2A	GTSF1			CRKRS	ZNF557		
PPP2R5B	NCKAP1L			NEUROD2	INSR		
GPHA2	PDE1B			PPP1R1B	ARHGEF18		
BATF2	PPP1R1A			STARD3	PEX11G		
ARL2	LACRT			TCAP	MCOLN1		
SNX15	MUCL1			PNMT	PNPLA6		
SAC3D1	NEUROD4			PERLD1	XAB2		
NAALADL1	METTL7B			ERBB2	STXBP2		
CDCA5	ITGA7			C17orf37	C19orf59		
ZFPL1	BLOC1S1			IKZF3	FCER2		
C11orf2	CD63			ZPBP2	CD209		
FAU	GDF11			GSDMB	CLEC4M		
MRPL49	CIP29			ORMDL3	EVI5L		
SYVN1	ORMDL2			GSDMA	LRRC8E		
CAPN1	DNAJC14			PSMD3	MAP2K7		
SLC22A20	MMP19			CSF3	SNAPC2		
POLA2	WIBG			MED24	TIMM44		
CDC42EP2	DGKA			THRA	ELAVL1		
DPF2	SILV			NR1D1	CCL25		
TIGD3	CDK2			CASC3	FBN3		
SLC25A45	RAB5B			RAPGEFL1	LASS4		
FRMD8	IKZF4			WIPF2	NDUFA7		
SCYL1	RPS26			CDC6	RPS28		

LTBP3	ERBB3			RARA	ANGPTL4		
SSSCA1	PA2G4			TOP2A	RAB11B		
FAM89B	RPL41			IGFBP4	MARCH2		
KCNK7	ZC3H10			TNS4	HNRNPM		
MAP3K11	FAM62A			CCR7	PRAM1		
SIPA1	MYL6B			SMARCE1	ZNF414		
RELA	MYL6			KRT222P	MYO1F		
KAT5	SMARCC2			KRT24	ADAMTS10		
RNASEH2C	RNF41			KRT25	ZNF558		
DKFZp761E198	SLC39A5			KRT27	MBD3L1		
OVOL1	ANKRD52			KRT28	MUC16		
SNX32	COQ10A			KRT10	ZNF317		
MUS81	CS			TMEM99	ZNF559		
EFEMP2	CNPY2			KRT12	ZNF177		
CTSW	PAN2			KRT20	ZNF266		
FIBP	IL23A			KRT23	ZNF560		
FOSL1	STAT2			KRTAP4-5	ZNF426		
C11orf68	APOF			KRTAP4-4	ZNF121		
DRAP1	TIMELESS			KRTAP4-2	ZNF561		
TSGA10IP	MIP			KRTAP4-1	ZNF562		
SART1	GLS2			KRTAP9-9	FBXL12		
EIF1AD	RBMS2			KRTAP9-4	PIN1		
BANF1	BAZ2A			KRTAP17-1	OLFM2		
CST6	ATP5B			KRT33A	COL5A3		
CATSPER1	PTGES3			KRT33B	RDH8		
GAL3ST3	NACA			KRT34	C3P1		
SF3B2	HSD17B6			KRT31	C19orf66		
PACS1	SDR9C7			KRT37	ANGPTL6		
KLC2	RDH16			KRT38	PPAN		

RAB1B	GPR182			KRT32	EIF3G		
CNIH2	ZBTB39			KRT36	DNMT1		
YIF1A	TAC3			KRT13	S1PR2		
TMEM151A	MYO1A			KRT15	MRPL4		
RIN1	NAB2			KRT19	ICAM1		
BRMS1	STAT6			KRT9	ICAM4		
B3GNT1	LRP1			EIF1	ICAM5		
SLC29A2	SHMT2			GAST	FDX1L		
NPAS4	NDUFA4L2			HAP1	RAVER1		
MRPL11	STAC3			JUP	ICAM3		
PELI3	R3HDM2			SC65	TYK2		
DPP3	INHBC			FKBP10	CDC37		
BBS1	INHBE			NT5C3L	PDE4A		
ACTN3	GLI1			KLHL10	KEAP1		
CTSF	ARHGAP9			KLHL11	S1PR5		
CCDC87	MARS			ACLY	ATG4D		
CCS	DDIT3			TTC25	KRI1		
RBM14	DCTN2			CNP	CDKN2D		
RBM4	KIF5A			DNAJC7	SLC44A2		
RBM4B	PIP4K2C			NKIRAS2	ILF3		
SPTBN2	DTX3			DHX58	DNM2		
C11orf80	GEFT			KAT2A	CARM1		
RCE1				HSPB9	YIPF2		
LRFN4				RAB5C	C19orf52		
PC				KCNH4	SMARCA4		
SYT12				HCRT	LDLR		
RHOD				GHDC	SPC24		
KDM2A				STAT5B	KANK2		
ADRBK1				STAT5A	TSPAN16		

SSH3					STAT3	RAB3D		
POLD4					PTRF	LOC126075		
CLCF1					ATP6V0A1	LPPR2		
RAD9A					NAGLU	C19orf39		
PTPRCAP					COASY	EPOR		
CORO1B					MLX	RGL3		
CABP4					PSMC3IP	CCDC151		
TMEM134					FAM134C	PRKCSH		
AIP					TUBG1	ELAVL3		
PITPNM1					TUBG2	ZNF653		
CDK2AP2					PLEKHH3	ECSIT		
CABP2					CCR10	CNN1		
GSTP1					CNTNAP1	ACP5		
NDUFV1					EZH1	ZNF823		
TBX10					RAMP2	ZNF491		
ACY3					VPS25	ZNF439		
ALDH3B2					WNK4	ZNF700		
UNC93B1					CNTD1	ZNF433		
ALDH3B1					BECN1	ZNF20		
NDUFS8					PSME3	ZNF625		
TCIRG1					AOC2	ZNF136		
CHKA					AOC3	ZNF44		
SUV420H1					LOC388387	ZNF563		
C11orf24					G6PC	ZNF442		
LRP5					AARSD1	ZNF709		
SAPS3					RUNDC1	ZNF564		
GAL					RPL27	ZNF490		
MTL5					IFI35	ZNF791		
CPT1A					VAT1	MAN2B1		

IGHMBP2				RND2	C19orf56		
MRGPRF				BRCA1	MORG1		
TPCN2				NBR1	DHPS		
MYEOV				ARL4D	TNPO2		
CCND1				DHX8	C19orf43		
ORAOV1				ETV4	ASNA1		
FGF4				MEOX1	BEST2		
FGF3				SOST	HOOK2		
ANO1				DUSP3	JUNB		
FADD				MPP3	PRDX2		
PPFIA1				CD300LG	RNASEH2A		
CTTN				MPP2	RTBDN		
SHANK2				NAGS	DNASE2		
DHCR7				TMEM101	KLF1		
NADSYN1				LSM12	GCDH		
KRTAP5-9				G6PC3	FARSA		
RNF121				HDAC5	CALR		
NUMA1				C17orf53	RAD23A		
LRTOMT				ASB16	GADD45GIP1		
C11orf51				TMUB2	DAND5		
FOLR3				UBTF	NFIX		
FOLR1				RUNDC3A	LYL1		
FOLR2				SLC25A39	TRMT1		
INPPL1				GRN	NACC1		
PHOX2A				ITGA2B	STX10		
CLPB				GPATCH8	CACNA1A		
PDE2A				FLJ35848	CCDC130		
ARAP1				CCDC43	MRI1		
STARD10				DBF4B	C19orf53		

FCHSD2				ADAM11	ZSWIM4		
P2RY2				GJC1	C19orf57		
P2RY6				HIGD1B	CC2D1A		
ARHGEF17				EFTUD2	PODNL1		
RELT				CCDC103	C19orf72		
FAM168A				GFAP	RFX1		
PLEKHB1				C1QL1	RLN3		
RAB6A				DCAKD	IL27RA		
MRPL48				NMT1	PRKACA		
CHCHD8				PLCD3	ASF1B		
PAAF1				ACBD4	LPHN1		
UCP2				HEXIM1	DDX39		
UCP3				HEXIM2	PKN1		
C2CD3				FMNL1	PTGER1		
PPME1				C17orf46	GIPC1		
P4HA3				MAP3K14	DNAJB1		
PGM2L1				PLEKHM1	GPSN2		
KCNE3				C17orf69	EMR3		
POLD3				CRHR1	ZNF333		
CHRDL2				IMP5	EMR2		
RNF169				LOC100128977	OR7A5		
XRRA1				MAPT	OR7C2		
SPCS2				KIAA1267	SLC1A6		
NEU3				NSF	CCDC105		
SLCO2B1				WNT3	CASP14		
ARRB1				WNT9B	SYDE1		
RPS3				GOSR2	ILVBL		
KLHL35				CDC27	NOTCH3		
GDPD5				MYL4	EPHX3		

SERPINH1				ITGB3	BRD4		
MAP6				C17orf57	AKAP8		
MOGAT2				NPEPPS	AKAP8L		
DGAT2				KPNB1	WIZ		
UVRAG				TBKBP1	PGLYRP2		
WNT11				TBX21	CYP4F22		
PRKRIR				OSBPL7	CYP4F8		
C11orf30				MRPL10	CYP4F3		
LRRC32				LRRC46	CYP4F12		
TSKU				SCRN2	OR10H1		
ACER3				SP2	CYP4F2		
B3GNT6				PNPO	CYP4F11		
CAPN5				ATAD4	FLJ25328		
MYO7A				CDK5RAP3	TPM4		
GDPD4				COPZ2	RAB8A		
				NFE2L1	HSH2D		
				CBX1	CIB3		
				SNX11	FAM32A		
				SKAP1	AP1M1		
				HOXB2	KLF2		
				HOXB3	EPS15L1		
				HOXB4	CALR3		
				HOXB5	C19orf44		
				HOXB6	CHERP		
				HOXB7	SLC35E1		
				HOXB8	MED26		
				HOXB9	C19orf42		
				PRAC	TMEM38A		
				HOXB13	NWD1		

				TTLL6	CPAMD8		
				CALCOCO2	HAUS8		
				ATP5G1	MYO9B		
				UBE2Z	USE1		
				SNF8	NR2F6		
				GIP	C19orf62		
				IGF2BP1	ANKLE1		
				B4GALNT2	ABHD8		
				GNGT2	MRPL34		
				ABI3	GTPBP3		
				PHOSPHO1	PLVAP		
				ZNF652	BST2		
				PHB	FAM125A		
				NGFR	NXNL1		
				NXPH3	PGLS		
				SPOP	FAM129C		
				SLC35B1	GLT25D1		
				FAM117A	UNC13A		
				MYST2	MAP1S		
				TAC4	FCHO1		
				DLX4	B3GNT3		
				DLX3	INSL3		
				ITGA3	JAK3		
				PDK2	RPL18A		
				SAMD14	SLC5A5		
				PPP1R9B	CCDC124		
				SGCA	KCNN1		
				COL1A1	ARRDC2		
				TMEM92	IL12RB1		

				XYLT2	MAST3		
				MRPL27	PIK3R2		
				EME1	IFI30		
				LRRC59	MPV17L2		
				ACSF2	RAB3A		
				CHAD	PDE4C		
				MYCBPAP	JUND		
				EPN3	LSM4		
				SPATA20	PGPEP1		
				CACNA1G	LRRC25		
				ABCC3	SSBP4		
				ANKRD40	ELL		
				CROP	FKBP8		
				C17orf73	C19orf50		
				WFIKKN2	UBA52		
				TOB1	CRLF1		
				SPAG9	TMEM59L		
				NME1	KLHL26		
				NME1-NME2	CRTC1		
				MBTD1	UPF1		
				CA10	GDF1		
				KIF2B	COPE		
				TOM1L1	DDX49		
				COX11	HOMER3		
				STXBP4	SFRS14		
				HLF	ARMC6		
				MMD	SLC25A42		
				TMEM100	TMEM161A		
				PCTP	MEF2B		

				ANKFN1	LOC729991-MEF2B		
				NOG	RFXANK		
				DGKE	NCAN		
				TRIM25	TM6SF2		
				COIL	KIAA0892		
				SCPEP1	GATAD2A		
				AKAP1	NDUFA13		
				MSI2	CILP2		
				MRPS23	PBX4		
				CUEDC1	GMIP		
				VEZF1	ATP13A1		
				SFRS1	ZNF101		
				DYNLL2	ZNF14		
				EPX	LOC284440		
				MKS1	ZNF253		
				LPO	ZNF93		
				MPO	ZNF682		
				BZRAP1	ZNF90		
				RNF43	ZNF486		
				HSF5	ZNF626		
				MTMR4	ZNF85		
				Sep 04	ZNF714		
				C17orf47	ZNF431		
				TEX14	ZNF708		
				LOC645545	ZNF493		
				RAD51C	ZNF429		
				PPM1E	ZNF100		
				TRIM37	ZNF43		
				FAM33A	ZNF257		

				PRR11	ZNF98		
				C17orf71	ZNF492		
				GDPD1	ZNF91		
				YPEL2	ZNF675		
				DHX40			
				CLTC			
				PTRH2			
				TMEM49			
				TUBD1			
				RPS6KB1			
				HEATR6			
				CA4			
				USP32			
				C17orf64			
				APPBP2			
				PPM1D			
				BCAS3			
				TBX2			
				TBX4			
				BRIP1			
				INTS2			
				MED13			
				EFCAB3			
				TLK2			
				MRC2			
				MARCH10			
				TANC2			
				CYB561			
				ACE			

				KCNH6			
				WDR68			
				MAP3K3			
				STRADA			
				CCDC47			
				DDX42			
				FTSJ3			
				PSMC5			
				SMARCD2			
				CD79B			
				SCN4A			
				ICAM2			
				ERN1			
				TEX2			
				PECAM1			
				C17orf60			
				POLG2			
				DDX5			
				CCDC45			
				SMURF2			
				LOC146880			
				PLEKHM1P			
				FLJ32065			
				GNA13			
				RGS9			
				AXIN2			
				CCDC46			
				APOH			
				PRKCA			

				CACNG5				
				CACNG4				
				CACNG1				
				HELZ				
				PSMD12				
				PITPNC1				
				NOL11				
				BPTF				
				C17orf58				
				SLC16A6				
				ARSG				
				WIPI1				
				PRKAR1A				
				FAM20A				
				ABCA8				
				ABCA9				
				ABCA6				
				ABCA10				
				ABCA5				
				MAP2K6				
				KCNJ16				
				KCNJ2				
				SOX9				
				SLC39A11				
				SSTR2				
				COG1				
				FAM104A				
				C17orf80				
				CDC42EP4				

				SDK2				
				C17orf54				
				RPL38				
				TTYH2				
				DNAI2				
				KIF19				
				GPR142				
				GPRC5C				
				CD300A				
				CD300LB				
				CD300LD				
				CD300E				
				RAB37				
				CD300LF				
				SLC9A3R1				
				NAT9				
				TMEM104				
				GRIN2C				
				FADS6				
				OTOP2				
				OTOP3				
				C17orf28				
				CDR2L				
				ICT1				
				ATP5H				
				KCTD2				
				SLC16A5				
				ARMC7				
				NT5C				

				HN1				
				SUMO2				
				NUP85				
				MRPS7				
				MIF4GD				
				SLC25A19				
				GRB2				
				KIAA0195				
				CASKIN2				
				TSEN54				
				LLGL2				
				MYO15B				
				RECQL5				
				SAP30BP				
				ITGB4				
				GALK1				
				UNK				
				UNC13D				
				WBP2				
				TRIM47				
				TRIM65				
				MRPL38				
				FBF1				
				ACOX1				
				LOC100134934				
				CDK3				
				EVPL				
				SRP68				
				GALR2				

				EXOC7				
				FOXJ1				
				RNF157				
				FAM100B				
				QRICH2				
				PRPSAP1				
				SPHK1				
				UBE2O				
				AANAT				
				RHBDF2				
				CYGB				
				ST6GALNAC2				
				ST6GALNAC1				
				MXRA7				
				JMJD6				
				SFRS2				
				MFSD11				
				MGAT5B				
				SEC14L1				
				Sep 09				
				TNRC6C				
				TMC6				
				TMC8				
				SYNGR2				
				TK1				
				AFMID				
				BIRC5				
				PGS1				
				CYTH1				

				USP36			
				TIMP2			
				LGALS3BP			
				CANT1			
				C1QTNF1			
				ENGASE			
				HRNBP3			
				CBX8			
				CBX4			
				TBC1D16			
				CCDC40			
				GAA			
				EIF4A3			
				CARD14			
				SGSH			
				SLC26A11			
				KIAA1618			
				RNF213			
				FLJ35220			
				NPTX1			
				KIAA1303			
				CHMP6			
				BAIAP2			
				AATK			
				AZI1			
				C17orf56			
				SLC38A10			
				C17orf55			
				TMEM105			

				BAHCC1			
				C17orf70			
				NPLOC4			
				PDE6G			
				HGS			
				MRPL12			
				SLC25A10			
				ASPSCR1			
				RAC3			
				DUS1L			
				CCDC57			
				SLC16A3			
				CSNK1D			
				CD7			
				SECTM1			
				TEX19			
				C17orf101			
				HEXDC			
				C17orf62			
				NARF			
				FOXK2			
				WDR45L			
				RAB40B			
				FN3KRP			
				FN3K			
				TBCD			
				B3GNTL1			

Suppl. Table 7. Pathways in N1 primary tumors and LNM. Unique alterations in N1 primary tumors and LNMs with representative genes that play important roles in cancer associated pathways.

**Cancer associated pathways of N1 tumors**	
**Gene Symbol**	**Gene Name**
CASP3	caspase 3, apoptosis-related cysteine peptidase
FGF10	fibroblast growth factor 10
COL4A2	collagen, type IV, alpha 2
FOXO1	forkhead box O1
RAF1	v-raf-1 murine leukemia viral oncogene homolog 1
EGF	epidermal growth factor
TP53	tumor protein p53
RASSF1	Ras association (RalGDS/AF-6) domain family member 1
MLH1	mutL homolog 1, colon cancer, nonpolyposis type 2 (E. coli)
FGF5	fibroblast growth factor 5
FLT3	fms-related tyrosine kinase 3
PDGFRA	platelet-derived growth factor receptor, alpha polypeptide
TGFBR2	transforming growth factor, beta receptor II (70/80kDa)
WNT5A	wingless-type MMTV integration site family, member 5A
BMP2	bone morphogenetic protein 2
RB1	retinoblastoma 1
CTNNB1	catenin (cadherin-associated protein), beta 1, 88kDa
PIK3R5	phosphoinositide-3-kinase, regulatory subunit 5
NFKB1	nuclear factor of kappa light polypeptide gene enhancer in B-cells 1
MITF	microphthalmia-associated transcription factor
LAMB2	laminin, beta 2 (laminin S)
COL4A1	collagen, type IV, alpha 1
KIT	v-kit Hardy-Zuckerman 4 feline sarcoma viral oncogene homolog
FGF11	fibroblast growth factor 11
DVL2	dishevelled, dsh homolog 2 (Drosophila)
VEGFC	vascular endothelial growth factor C
RARB	retinoic acid receptor, beta
FGF9	fibroblast growth factor 9 (glia-activating factor)
RHOA	ras homolog family member A
FGF2	fibroblast growth factor 2 (basic)
PPARG	peroxisome proliferator-activated receptor gamma
VHL	von Hippel-Lindau tumor suppressor, E3 ubiquitin protein ligase
WNT7A	wingless-type MMTV integration site family, member 7A
SKP2	S-phase kinase-associated protein 2, E3 ubiquitin protein ligase
APPL1	adaptor protein, phosphotyrosine interaction, PH domain and leucine zipper containing 1
HHIP	hedgehog interacting protein
LEF1	lymphoid enhancer-binding factor 1
FGF14	fibroblast growth factor 14
BRCA2	breast cancer 2, early onset
CRK	v-crk sarcoma virus CT10 oncogene homolog (avian)
IL8	interleukin 8
CCNA1	cyclin A1
MAPK10	mitogen-activated protein kinase 10

Chemokine-signaling pathway in LNMs	
**Gene Symbol**	**Gene Name**
VAV3	vav 3 guanine nucleotide exchange factor
CCL24	chemokine (C-C motif) ligand 24
CCL26	chemokine (C-C motif) ligand 26
NRAS	neuroblastoma RAS viral (v-ras) oncogene homolog
RAP1A	RAP1A, member of RAS oncogene family
ADCY4	adenylate cyclase 4
GNG3	guanine nucleotide binding protein (G protein), gamma 3
RAC1	ras-related C3 botulinum toxin substrate 1 (rho family, small GTP binding protein Rac1)
ADCY3	adenylate cyclase 3
GNAI3	guanine nucleotide binding protein (G protein), alpha inhibiting activity polypeptide 3

Suppl. Table 8. Genes of significant alterations of cluster B with activation in cancer associated pathways.

Loss	Loss	Loss	Loss	Gain	Loss	Gain	Gain	Gain	Gain	Gain	Gain
**2q11-2q35**	**4q12-4q35**	**5q11-5q35**	**9p24-9p13**	**9q33-9q34**	**10q11-10q23**	**11p11**	**11q12-11q13**	**12q13**	**17q11-17q25**	**19p13-19p12**	**20q11-20q12**
IL1R1	CXCL9	IL3	IFNE	LAMC3	MAPK8	DDB2	POLA2	WNT1	CCL7	PIAS4	GDF5
CXCR4	CXCL10	IL6ST	IFNA8	TRAF1	NCOA4	CD82	POLD3	DHH	CCR10	IL12RB1	ID1
ACVR1	TLR2	TSLP	IFNA2	TRAF2	RET		RNASEH2	WNT10B	CCL16	EPOR	RBL1
BMPR2	SPP1	PIK3R1	IL11RA	RXRA	NCOA4		POLD4	GLI1	CCL15	JAK3	BCL2L1
EDAR	CXCL11	IL13	IFNB1				FEN1	AMHR2	CCL23	TYK2	E2F1
INHBB	TLR3	IL4	IFNK				BAD	INHBC	CCR7	PIK3R2	
IL18RAP	IL8	IL9	CNTFR				CCND1	INHBE	STAT5B	MAP2K7	
IL1R2	NFKB1	SPRY4	IFNW1				FGF4	SP1	CCL5	CACNA1A	
ACVR2A	MAPK10	CSF2	JAK2				WNT11		GRB2	GADD45B	
IL1A	CXCL9	IL5					RELA		CCL8	ECSIT	
IL1B	CXCL5	IL12B					GSTP1		GNGT2	MKNK2	
IL18R1	CXCL13						FADD		CCL14	PRKACA	
GLI2	CXCL3						FGF3		CCL13	MAP2K2	
WNT6	CXCL6						VEGFB		CCL3	JUND	
FN1	CXCL2						MAP4K2		STAT3	VAV1	
PAX8	PF4						MAP3K11		CCL18	PRKACA	
RALB	PPBP						ARRB1		CCL11	SHC2	
WNT10A							RPS6KA4		MAP3K3	GNG7	
ITGA6							RASGRP2		CACNG4	PIK3R2	
STAT1							CCND1		CACNA1G	CCL25	
ITGAV							LRP5		NF1		
FZD5							WNT11		TAOK1		
CASP8							PPP2R5B		NLK		
FZD7							FOSL1		PRKCA		
							PLCB3		RAC3		
									DUSP14		
									GRB2		
									CACNG5		
									CACNB1		
									CACNG1		

									DUSP3<br>MAP2K6<br>MAP3K14<br>MAPT<br>RARA<br>JUP<br>ERBB2<br>STAT5B<br>PRKCA<br>RAC3<br>ITGA2B<br>GRB2<br>ITGA3<br>BIRC5<br>AXIN2<br>STAT5A<br>WNT3<br>TRAF4<br>NOS2<br>WNT9B		

This genes listed, showed a significant activation in the JAK-STAT-, SCLC-, Thyroid cancer-, p53-, DNA replication-, MAPK-, Wnt-, TGF-beta-, Hedgehog- and Chemokin signaling pathways in the KEGG-pathway analysis.

Suppl. Table 9. Genes that play an important role in cancer associated pathways from unique gains (3p, 4q, 5p, 13q) and losses (17p, 20p) of N1 primary tumors matched against the Therapeutic Targets Database.

Cytoband	Gene symbol	Drug name	Drug status	Drug type	Indication
3p14	WNT5A	Box-5	Investigative	Antibody	Melanoma
3p21	RHOA	ES-285	Phase 1	Inhibitor	Solid tumors
3p22	MLH1	HG-1068	Investigative	Antibody	Cancer
3p24	TGFBR2	TGF-BR2 mAb	Phase 1	Antibody	Cancer
3p25	VHL	STF-62247	Investigative	Antibody	Renal cell carcinoma
3p25	PPARG	IDR-105	**Approved**	Modulator	Diabetes mellitus
3p25	PPARG	FARGLITAZAR (+5 Inhibitors)	Phase 3	Inhibitor	Diabetes mellitus
3p25	WNT7A	FT-301	Investigative	Inhibitor	Muscle wasting disease
4q21	FGF5	AS-902330	Phase 2	Modulator	Arthropathy
4q24	NFKB1	P54	Phase 2	Modulator	Cancer
4q34	VEGFC	VGX-100	Phase 1	Modulator	Solid tumours
5p12	FGF10	Repifermin	Phase 2	Modulator	Oral mucositis
5p13	LIFR	Emfilermin	Phase 1/2	Inhibitor	Infertility
5p13	GHR	Sermorelin (+1 Binder)	**Approved**	(Binder)	Growth hormone deficiency
13q12	FLT3	Ponatinib (+1 Modulator)	**Approved**	Modulator	ALL
13q12	FLT3	PLX-3397 (+ 2 Inhibitors)	Phase 3	Inhibitor	Hematological malignancies
13q12	FLT3	BMS-690514 (+ 5 Inhibitors)	Phase 2	Inhibitor	Cancer
13q12	FLT3	4SC-203 (+3 Inhibitors)	Phase 1	Inhibitor	Solid tumors
13q14	FOXO1	AS-1708727	Investigative	Modulator	Hypertriglyceridemia
13q33	FGF14	Immunotoxin conjugate	Investigative	Inhibitor	Solid tumors
13q12	FLT1	VATALANIB	Phase 2	Inhibitor	Pancreatic cancer
17p13	CRK	ARRY-614	Phase 1	Modulator	Myelodysplastic syndromes
17p13	TP53	Cenersen	Phase 2	Antisense	ALL
20p12	BMP2	DWP-431	Phase 3	Modulator	Bone development disorder

Suppl. Table 10. Genes from isolated lymph node metastasis gains matched against the Therapeutic Targets Database.

Cytoband	Gene symbol	Drug Name	Drug status	Drug Type	Indication
1p13	SARS	Indinavir	**Approved**	Inhibitor	HIV Infection
1p13	CSF1	Filgrastim	**Approved**	Modulator	Neutropenia
1p13	CSF1	GTP-14564	Investigative	Inhibitor	Discovery Agent
1p13	CHIA	Ecopipam (+ 82 Inhibitors)	**Approved**	Inhibitor	Parkinson's Disease
1p13	CHIA	Odapipam (+ 11 Antagonists)	**Approved**	Antagonist	Parkinson's Disease
1p13	KCND3	CDE-5498	Investigative	Antagonist	Heart arrhythmia
1p13	TSPAN2	BI-836826 (+ 1 Antibody)	Phase 1	Antibody	CLL, Cancer
1p13	TSPAN2	Anti-CD9 mAb	Investigative	Antibody	Gastric cancer
1p13	NGF	Lestaurtinib	**Approved**	Inhibitor	Leukemia
1p13	NGF	PF-4383119	Phase 3	Antagonist	HCC
1p13	NGF	MEDI-578	Phase 1	Antibody	Pain
1p13	NGF	NT-3	Phase 2	Modulator	Constipation
1p13	CD58	PDL-241	Investigative	Antibody	Immune disorder
1p13	NOTCH2	Tarextumab	Phase 2	Antibody	Pancreatic cancer
2p23	POMC	(Dihydromorphine)	Investigative	*(Agonist)*	Discovery Agent
2p23	OTOF	SAR228810	Phase 1	Antibody	Alzheimer's disease
2p23	KHK	JNJ-28165722	Investigative	Inhibitor	Diabetes mellitus
2p23	PREB	Daporinad	Phase 2	Inhibitor	Leukemia
2p23	CAD	N-(Phosphonacetyl)-L-Aspartic Acid	Investigative	Inhibitor	Discovery Agent
7p22	PDGFA	CNVN-202	Phase 1/2	Suppressor	Non-small cell lung cancer
7p22	GPER	G15 (+ 1 Antagonist)	Investigative	Antagonist	Discovery Agent
7p22	SDK1	4-HYDROXYTAMOXIFEN	Phase 2	Inhibitor	Discovery Agent
7p22	SDK1	KAI-9803	Phase 1/2	Inhibitor	Acute myocardial infarction
7p22	PMS2	HG-1077	Investigative	Inhibitor	Cancer
7p22	RAC1	EHT-1864	Investigative	Inhibitor	Alzheimer's disease
7p22	RAC1	Selective Rac1/1b GTPase nucleotide binding inhibitors	Investigative	Inhibitor	Cancer
7q11	HIP1	(AQX-1125)	Phase 2	*(Agonist)*	COPD
7q11	POR	Encapsulated cells	Phase 2	Modulator	Breast cancer
7q11	POR	DuP-630	Investigative	Modulator	Dermatitis
7q11	POR	DuP-983	Investigative	Modulator	Pruritus
7q11	HSPB1	SB-242235	Phase 2	Inhibitor	Arthritic
7q11	HSPB1	OGX-427	Phase 2	Antisense	Breast cancer
11q12	CNTF	NT-501 CNTF	Phase 1/2	Modulator	Ovarian cancer
11q12	GIF	ISO-1	Phase 1	Inhibitor	Discovery Agent
11q12	GIF	Anti-MIF antibodies	Phase 4	Inhibitor	Autoimmune disease

11q12	GIF	ISIS 112690 (+ 17 Inhibitors)	Investigative	Inhibitor	Discovery Agent
11q12	CD6	Itolizumab	Phase 3	Antibody	Diabetes mellitus type 1
11q12	CD5	MAT-304	Investigative	Antibody	Autoimmune disease
11q12	SCGB1D2	HG-1182	Investigative	Inhibitor	Cancer
11q12	ASRGL1	GRASPA	Phase 2/3	Modulator	Leukemia
11q12	ASRGL1	Calaspargase pegol	Phase 3	Modulator	Leukemia
11q12	SLC3A2	IGN523	Phase 1	Antibody	Leukemia
11q12	CHRM1	(AM-831)	Phase 1	*(Agonist)*	Schizophrenia
11q12	SLC22A6	Probenecid	**Approved**	Blocker	Hyperuricemia; Gout
11q12	SLC22A6	FLUORESCEIN	**Approved**	Inhibitor	Ocular disease
11q12	SLC22A6	Buproprion+zonisamide	Phase 2	Blocker	Obesity
11q12	SLC22A6	ADIPATE (+ 4 Inhibitors)	Investigative	Inhibitor	Discovery Agent
12q14	CDK4	Capridine-beta	**Approved**	Inhibitor	Psoriasis
12q14	CDK4	LEE011	Phase 3	Inhibitor	Cancer
12q14	CDK4	LY2835219	Phase 3	Modulator	Cancer
12q14	CDK4	Palbociclib	Phase 3	Regulator	Cancer
12q14	CDK4	BAY 10-00394 (+ 4 Inhibitors)	Phase 2	Inhibitor	Cancer
12q14	CDK4	AG-024322 (+ 5 Inhibitors)	Phase 1	Inhibitor	Cancer
12q14	CDK4	R547 (+ 1 Inhibitor)	Phase 1	Inhibitor	Solid tumors
12q14	MDM2	CGM097 (+ 2 Inhibitors)	Phase 1	Inhibitor	Solid tumors
12q14	MDM2	JNJ-26854165 (+ 7 Modulators)	Phase 1	Modulator	NSCLC + Prostate cancer
12q14	MDM2	AMG 232 (+ 1 Modulator)	Phase 1/2	Modulator	Cancer
12q14	MDM2	NU-8231 (+ 3 Inhibitors)	Investigative	Inhibitor	Discovery Agent
13q13	POSTN	FT-201	Investigative	Inhibitor	Insulin dependent diabetes
14q12	NEDD8	MLN4924	Phase 1	Inhibitor	Advance malignancies
14q12	CTSG	Aloxistatin	Phase 3	Inhibitor	Neurological disease
14q12	CTSG	compound 1 (+ 2 Inhibitors)	Investigative	Inhibitor	Discovery Agent
14q12	CTSG	CEP-18770	Phase 1/2	Inhibitor	Discovery Agent
14q12	CTSG	Dermolastin (+1 Inhibitor)	Phase 2	Inhibitor	Atopic Dermatitis

Suppl. Table 11. Genes that play an important role in cancer associated pathways from CNAs of cluster B matched against the Therapeutic Targets Database

Cytoband	Gene symbol	Drug name	Drug status	Drug type	Indication
2q11-2q35	CXCR4	SURADISTA (+6 Modulators)	Investigative	Modulator	Cancer
2q11-2q35	FN1	AS1409	Phase 1	Binder	Melanoma
2q11-2q35	CASP8	Glionitrin A	Investigative	Modulator	Cancer
4q12-4q35	TLR3	HspE7 (TLR3 agonist adjuvant)	Phase1/2	Agonist	Anal intraepithelial neoplasia
4q12-4q35	CXCL2	Laquinamod	Phase 3	Modulator	Multiple Sclerosis
4q12-4q35	IL4	PRX-321	Phase 2	Agonist	Brain Cancer, Kidney Cancer, Lung Cancer
9p24-9p13	IL11RA	Oprelvekin	**Approved**	Binder	Severe thrombocytopenia
9p24-9p13	CNTFR	PegCNTF	Phase 1	Agonist	Obesity
9p24-9p13	CNTFR	CNTF	Phase 3	Agonist	Obesity
9q33-9q34	RXRA	(5BETA)-PREGNANE-3,20-DIONE (+3 Inhibitors)	Experimental	Inhibitor	-
17q11-17q25	STAT3	Acitretin	**Approved**	Inhibitor	Psoriasis
17q11-17q25	STAT3	Atiprimod (+3 Inhibitors)	Phase 2	Inhibitor	Multiple myeloma, Cancer, Solid Tumors
17q11-17q25	ERBB2	Trastuzumab (+4 Antibodys)	**Approved** (rest Phase 2)	Antibody	Breast Cancer
17q11-17q25	ERBB2	Lapatinib (+ 18 Inhibitors	**Approved** (rest Phase 2)	Inhibitor	Breast Cancer
19p13-19p12	JAK3	Tofacitinib (+3 Inhibitors)	**Approved** (rest Phase 2)	Inhibitor	Rheumatoid Arthritis
19p13-19p12	TYK2	BI-853520	Phase 1	Inhibitor	Solid Tumors
20q11-20q12	E2F1	ARQ 761	Phase 1	Modulator	Solid Tumors
